# A Pediatric- and Adolescent-Focused Medication Abortion Curriculum for Multidisciplinary Trainees

**DOI:** 10.15766/mep_2374-8265.11553

**Published:** 2025-11-13

**Authors:** Julia H. Raney, Chelsea A. Garnett, Amanda E. Bryson, Lisa Mihaly, Sara M. Buckelew, Marissa Raymond-Flesch

**Affiliations:** 1 Assistant Professor of Pediatrics, Division of Adolescent and Young Adult Medicine, Stanford University; 2 Assistant Professor of Pediatrics and Director of Adolescent Medicine Pediatric Resident Rotation, Division of Adolescent and Young Adult Medicine, University of California San Francisco; 3 Assistant Professor of Pediatrics, Division of Adolescent and Young Adult Medicine, University of California San Francsico; 4 Associate Clinical Professor and Co-Director of Family Nurse Practitioner Program, Department of Family Health Care Nursing, University of California, San Francisco; 5 Professor of Pediatrics, Chief of Division of Adolescent and Young Adult Medicine, University of California San Francisco; 6 Professor of Pediatrics and Associate Fellowship Program Director, Division of Adolescent and Young Adult Medicine, University of California San Francisco; † Co-primary author

**Keywords:** Adolescent Medicine, Pediatrics, Abortion, Health Equity, Interprofessional Education, Women's Health, Case-Based Learning, Flipped Classroom

## Abstract

**Introduction:**

Post-Roe v. Wade, 22 states now ban or heavily restrict abortion, decreasing safe and timely access for adolescents and young adults (AYAs) and limiting the number of abortion providers being trained. Further, no AYA-focused medication abortion (MAB) curriculum exists. To fill this gap, we developed an AYA-focused MAB curriculum for pediatric trainees.

**Methods:**

Using Kern's Six Steps, we designed a blended curriculum of online modules (30-40 minutes) and a workshop (120 minutes). Using pre/post surveys, we assessed differences in multiple-choice knowledge questions and Likert scales evaluating values and intentions; open-ended responses were analyzed using directed qualitative content analysis.

**Results:**

Nine workshops were held over the 2023-2024 academic year, with a total of 52 learners completing the curriculum. Pre/post data are available from 29 learners, including 15 pediatric residents, 13 adolescent-focused nurse practitioner students, and one medical student. Learners demonstrated a significant increase in knowledge score after curriculum completion (60% vs. 90%; *p* < .01). Intentions to provide, refer, and advocate for MAB care did not change significantly (average of three questions on a 5-point Likert scale: 4.3 vs. 4.3; *p* = .92). Eighty-five percent of learners rated the overall curriculum as *excellent* or *outstanding*. Major themes included appreciating the opportunity to explore and anticipate challenging cases and finding case-based learning and role-play helpful.

**Discussion:**

Our curriculum improved trainees’ knowledge of MAB provision for AYAs, instilled confidence, and helped learners anticipate challenging abortion cases. The generalizability of skills learned may vary by local political climate, legal restrictions, and/or institutional support.

## Educational Objectives

By the end of this activity, learners will be able to:

1. Describe the unique health equity, developmental, and confidentiality considerations related to abortion care for adolescents and young adults (AYAs).
2. Discuss harm-reduction strategies to mitigate legal and medical complications for AYAs in abortion-restrictive states.
3. Examine values and beliefs related to pregnancy options and abortion care grounded in a reproductive justice framework.
4. Describe elements of a no-test medication abortion protocol including pregnancy options counseling, eligibility assessment, anticipatory guidance, and postabortion care.
5. Apply a no-test medication abortion protocol to an adolescent patient case.

## Introduction

Since the *Dobbs v. Jackson Women's Health Organization* Supreme Court decision in June 2022, the post-Roe reality has limited bodily autonomy for millions of patients, creating an urgent need to expand abortion training and access. Since the fall of Roe, 22 states have banned or significantly limited abortion access,^1^ and now only 35% of obstetrics and gynecology (OB/GYN) training programs offer routine abortion training,^2^ limiting the pipeline of future providers. The restrictions on reproductive freedom disproportionately impact adolescents and young adults (AYAs), a population that accounts for 36% of all US abortions.^3^ These legal changes compound the well-documented challenges that many AYAs already face in accessing safe abortion care, including cost and transportation limitations, longer distances from health care institutions, health insurance barriers, parental involvement requirements, and confidentiality concerns.^4-6^ These challenges may be particularly relevant for AYAs from marginalized groups including Black, Latine, low-income, disabled, and other minoritized identities who also experience inequitable abortion care access.^7,8^ Thus, there is an urgent need to increase pediatric provider education in abortion care for AYAs, who have unique confidentiality concerns and developmental considerations.

Pediatric clinicians are well positioned to expand their training in first-trimester abortion care to fill this urgent need for several reasons. First, a new opportunity for the provision of medication abortions (MABs) opened after no-test MAB protocols were developed during the COVID-19 pandemic.^9^ These new protocols support the provision of MABs without an examination, ultrasound, or blood tests for gestational age of up to 77 days.^9-11^ MABs are common, accounting for more than one half of abortions in the US.^12^ Given that 60% to 80% of AYA abortions occur before 10 weeks’ gestation when no-test MABs are an option,^3^ the majority of AYA abortions can be safely managed by primary care providers, who have the most experience caring for a pediatric population. Second, although no formal evaluation of patient or family preferences for abortion services in pediatric primary care settings exist, evaluations of adult perspectives are promising. Several studies have found that many adult patients prefer to access abortion care in primary care settings, where they have longstanding relationships and feel comfortable with providers or staff.^12,13^ Third, pediatric clinicians may be particularly well positioned to provide AYA MABs, given their expertise in the legal and developmental considerations related to treating AYAs. Fourth, pediatric health care providers often diagnose pregnancy, so they may be best able to provide timely MAB services, which is critical in some restrictive practice settings. Experts in adolescent medicine and OB/GYN have called upon pediatric providers as critical extenders of abortion care and have advocated for expanded training opportunities.^14-16^ With shifts in MAB protocols (such as no universal need for dating ultrasound), pediatric clinicians can obtain the necessary skills to offer and manage MAB, building on their expertise in providing developmentally appropriate sexual and reproductive health care.

As it stands, MAB training is not a standard component of pediatric training, and a review of *MedEdPORTAL* and the pediatric, family medicine, and OB/GYN literature demonstrates that there is currently no available MAB curriculum designed specifically for serving the AYA population. Such a curriculum must include explorations of confidentiality barriers and protections, including parental involvement laws; developmental stage-specific pregnancy options counseling framework; and psychosocial supports. To help fill this gap and promote pediatric health care learner competence in MABs, a relatively rare clinical experience, we designed the curriculum to optimally promote self-efficacy. Self-efficacy, a key construct of Bandura's Social Cognitive Theory, is facilitated through practice and observational learning.^17^ Bandura emphasized that modeling and observing the behaviors, attitudes, and emotional reactions of peers and role models contribute to developing self-efficacy. Vicarious learning-based curricula have been shown to improve knowledge, skills, and self-efficacy.^18,19^ Thus, the curriculum emphasizes case-based learning by using a flipped classroom approach and uses role-play followed by group discussions debriefing the cases.^20,21^

## Methods

### Facilitators

This work was designed by medical fellows, nurse practitioner faculty, and attending physician faculty of the University of California, San Francisco's (UCSF) Adolescent and Young Adult Medicine Division. The number of facilitators varied by number of learners (range of one to four). Each facilitator was an adolescent and young adult clinician with significant experience in adolescent development. Facilitators reviewed articles in key learning areas including health equity, MAB protocols, and harm reduction strategies (see articles in Appendix A). All facilitators were familiar with the entire workshop and able to lead any part of it depending on facilitator availability.

### Target Learners

Target learners included medical students, pediatric residents, and nurse practitioner students completing their adolescent and young adult medicine rotation or a member of UCSF's Leadership in Education in Adolescent Health Program.

### Curriculum

We used Kern's Six Steps to design, implement, and evaluate our AYA-focused MAB curriculum.^22,23^ For step one (problem identification), we reviewed the *MedEdPORTAL* and medical literature for existing AYA-focused MAB curricula and sent a national email request to adolescent medicine program directors to inquire about abortion training at their respective institutions. Through this review and outreach, we determined that no AYA-focused abortion curriculum nationally was available at that time. For step two (targeted needs assessment), we discussed this identified problem with UCSF and Children's Oakland pediatric residency leadership, who identified the lack of abortion curriculum as a deficit and opportunity for growth in pediatric residency education. For step three, our goals and objectives were modeled after those of existing curricula found in the internal medicine, family medicine, OB/GYN, and nurse practitioner education literature with additional adolescent-focused components added to address the gap in pediatric curricula. Step four, selection of educational strategies, was informed by Bandura's Social Cognitive Theory.^17,19^ Specifically, we decided to create a flipped classroom curriculum of four brief online modules (total time 30-40 minutes) and one workshop (total time 120 minutes). We chose this format to emphasize case-based learning and role play to enhance self-efficacy, a key component of social cognitive theory.^17^ The facilitator's guide (Appendix A) provides a detailed agenda. Step five involved presenting the workshop monthly for rotating learners. The final step, step six, included a pre- and postsurvey evaluating key components according to Social Cognitive Theory including knowledge, values, intentions, and self-competence.^17,21^

We initially developed a 90-minute in-person and synchronous virtual workshop. However, we lengthened the workshop to 120 minutes to allow for sufficient time to review the cases and made several changes on the basis of postsurveys. Major adaptations included (1) increasing didactic review of no-test MAB guidelines, (2) decreasing discussion of the shifting policy landscape (instead now refer to key resources, e.g., The Guttmacher Institute for up-to-date policy information), and (3) adapting cases to increase focus on role-play and MAB guidelines.

In the flipped classroom model, learners were first encouraged to complete the presurvey and four brief online modules before attending the synchronous 120-minute workshop. The four online modules consisted of brief videos with readings on adolescent pregnancy options counseling, MAB provision, postabortion care, and harm-reduction strategies (Appendices B–E). Adolescent themes included AYA-specific barriers and considerations regarding health equity, psychosocial development, and confidentiality. We presented the workshop via PowerPoint (Appendix F), with additional facilitator instructions provided in the notes section of the PowerPoint and via a practice case (Appendix G) with additional instructions in the case facilitator guide (Appendix H). Each workshop included six components. (1) We began with introductions, sharing the educational objectives and discussing the policy and AYA abortion access landscape that motivated us to develop this curriculum. (2) Next, we defined reproductive justice and reviewed how this framework informs our approach to AYA abortion care. (3) We then led a values clarification exercise, in which learners and facilitators explored values and beliefs related to pregnancy options and abortion care grounded in a reproductive justice framework. (4) Next, we briefly reviewed module content, and (5) split into smaller groups to analyze a clinical case. (6) Afterward, we came together as a larger group and encouraged learners to reflect on their experience with the workshop and ask any follow-up questions.

### Evaluation and Analysis

Pre- and postsurveys assessed key components of behavior change according to Social Cognitive Theory.^19^ Pre- and postsurveys assessed knowledge (eight multiple-choice knowledge questions); intentions (one multiple-choice and three Likert scale questions on future intentions to provide abortion, refer, and advocate for abortion access); and values (two open-ended questions, five Likert scale questions assessing moral acceptability of abortion in different scenarios; and three Likert scale questions assessing moral considerations regarding abortion and birth control intentions). Postsurveys only additionally assessed learner satisfaction (five Likert scale questions and two qualitative questions assessing satisfaction with curricular components) and self-efficacy (five Likert scale questions on provider confidence). Presurveys were sent out 1 week before the workshop and again the day before the workshop. Learners were asked to complete the postsurvey after the workshop, and another reminder was sent one week after the workshop. Pre- and postsurveys are shown in Appendices I and J. Descriptive statistics evaluated satisfaction and self-efficacy; the McNemar test and Wilcoxon signed-rank test assessed differences in knowledge and Likert-style intentions and values questions; we analyzed open-ended responses on curricular strengths, growth areas, and potential clinical challenges using a directed qualitative content analysis, noting how themes related to core educational objectives when applicable.

### Institutional Review Board

This project was deemed exempt by the University of California Institutional Review Board (Protocol No. 23-38335).

## Results

We held nine in-person workshops over the 2023-2024 academic year (October 2023-June 2024). Twenty-nine of 52 participants completed the pre- and postsurveys (56%); the majority of learners were pediatric residents (*n* = 15, 52%) or adolescent-focused nurse practitioner students (*n* = 13, 45%), with one medical student also participating (3%). Our learner sample was predominantly female (76% female, 21% male, 3% nonbinary; Table 1).

**Table 1.**
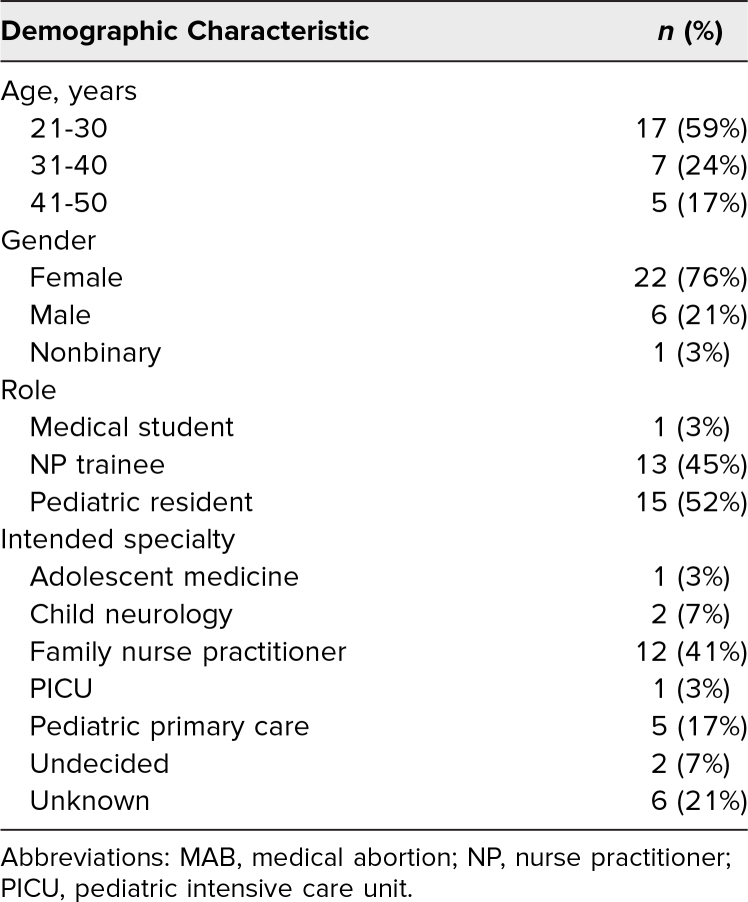
Sociodemographic Characteristics of the Adolescent MAB Curriculum Participants (*N* = 29)

### Quantitative Results

Learners demonstrated a significant increase in cumulative knowledge score after curriculum completion (60% vs. 90%; *p* < .01; Table 2), with significant improvements in the following knowledge areas: MAB no-test protocol (57% vs. 90%, *p* < .001); and post-MAB care (64% vs. 95%, *p* < .001). Overall learners’ feelings around caring for patients seeking abortions did not change significantly (average of eight questions on a 5-point Likert scale: 4.4 vs. 4.4, *p* = .76). Further, individually, none of the five Likert scale questions assessing moral acceptability of abortion in different scenarios or the three Likert scale questions assessing moral considerations regarding abortion and birth control intentions showed significant changes from pre- to postcurriculum completion (all *p* > .05). Similarly, intentions to provide, refer, and advocate for MAB care did not change significantly (average of three questions on a 5-point Likert scale: 4.3 vs. 4.3; *p* = .92). Among those with low intentions to provide MABs in the future, learners cited the following reasons: plan to subspecialize (nine), lack of training experiences (three), fear of community retaliation (one), and fear of personal safety (one).

**Table 2.**
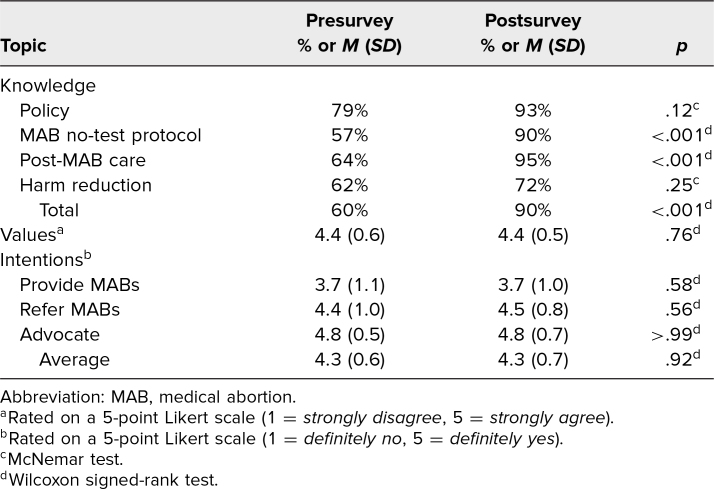
Learner Knowledge, Values, and Intentions Pre/Postcurriculum (*N* = 29)

After curriculum completion, learners felt *fairly* or *completely confident* in counseling on pregnancy (72%) and abortion options (66%) and in determining medical eligibility (88%), providing anticipatory guidance (89%), and managing common complications (55%) for MABs (Figure). Eighty-five percent of learners rated the overall curriculum excellent or outstanding.

**Figure.**
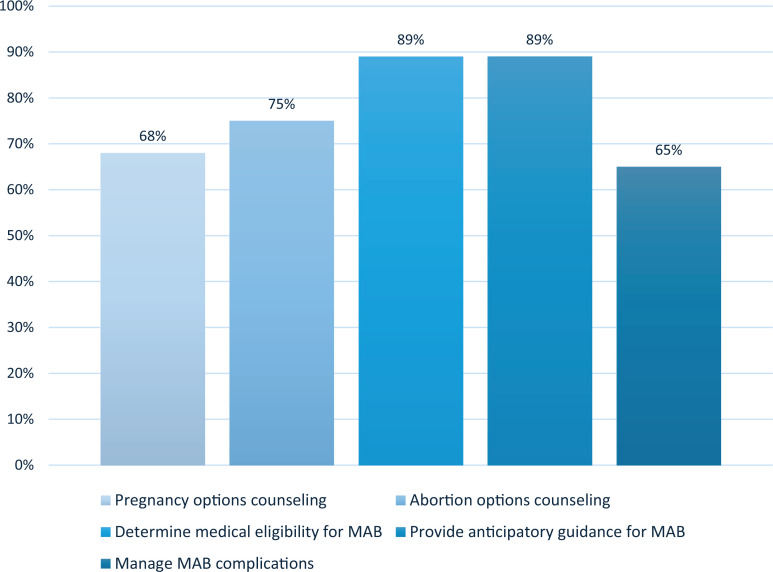
Percentage of learners who reported feeling fairly or completely confident after completion of the adolescents and young adults medication abortion (AYA MAB) curriculum (*N* = 29).

### Qualitative Results

In open-ended responses, learners described the curriculum as highly valuable and expressed excitement that this topic was covered at all in pediatric training (Table 3). Learners noted that the workshops gave them space to consider and anticipate potentially challenging abortion cases, resulting in increased confidence to be able to address these challenges in future clinical encounters (educational objective 3). The most valuable aspects of the curriculum included the inclusion of an MAB curriculum in a pediatric setting, case-based learning (including role-play; educational objective 4), the values clarification exercise (educational objective 3), and the inclusion of harm reduction strategies (educational objective 2). Suggestions for improvement were to include more time to answer cases (educational objective 4), shortening the values clarification exercise (educational objective 3), and ensuring a safe virtual space for the values clarification exercise (educational objective 3).

**Table 3.**
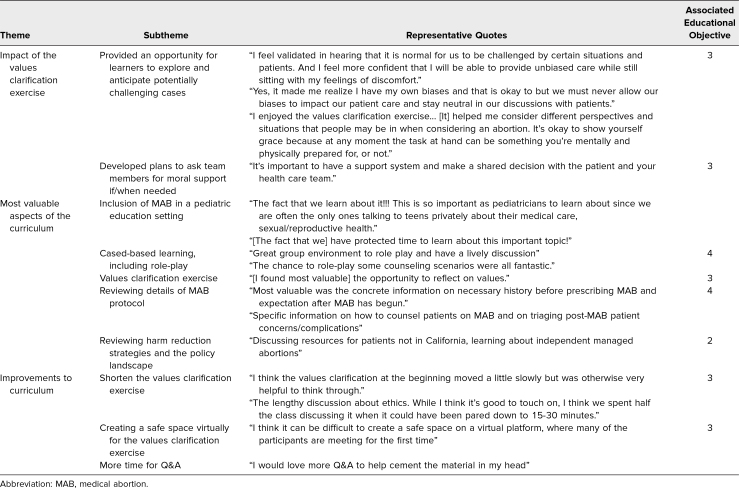
Learner Reflections on the Impact of Values Exploration, Curricular Strengths, and Growth Areas

## Discussion

Our curriculum improved trainee's knowledge of MAB provision for AYAs and instilled learner confidence in performing key aspects of MAB care, particularly in determining medical eligibility and providing anticipatory guidance for MABs. Importantly, our curriculum provided learners an opportunity to engage in a topic not typically covered in pediatric education and a space to consider and anticipate potentially challenging abortion cases. Our curriculum addresses the lack of available AYA-focused MAB materials and answers the call from experts in OB/GYN and pediatrics to expand MAB training opportunities for pediatric health care learners and thereby increase AYA access to abortion care.^14-16,24^

An additional strength of our curriculum includes the involvement of multidisciplinary facilitators (physicians and nurse practitioners) and learners (medical and nurse practitioner trainees). Learners had varying personal and professional experiences with abortion (e.g., some had worked as medical assistants or nurses in abortion settings), which facilitated rich discussions regarding abortion access and moral challenges. In addition to enhancing the learning experience, engaging multidisciplinary learners rather than focusing only on medical trainees expands our reach in educating future providers who will care for adolescents who may need abortion care.^24,25^ In addition, the curriculum was given to learners at varying time points (e.g., medical students, nurse practitioner students, residents) and training programs (Children's Oakland Pediatric Residency, UCSF Pediatric Residency, UCSF School of Nursing), which strengthens the reliability of our results.

Our curriculum evolved as we incorporated learners’ feedback. As discussed previously, we lengthened the workshop from 90 to 120 minutes on the basis of feedback requesting more time for practical and case-based learning. In lengthening this session, we added more content on patient counseling and included a brief review of topics covered in modules to solidify understanding. We also removed some discussion of harm reduction and policy information and instead focused on providing more in-depth discussion of patient counseling and MAB guidelines. Although we did not formally solicit feedback on the change in length and content of the workshop, we received no further free-response comments requesting more practice time. We additionally developed a practice of sending reminder emails 5 days and 1 day before the workshop to remind learners to view asynchronous modules in advance, which helped ensure that learners had some foundational knowledge prior to the workshop. Although we did not formally assess completion of premodules, through informal discussion during workshops, most learners shared they completed the prework modules. We therefore believe that learners primarily requested additional review of didactic materials to reinforce key educational points. Some of our early sessions were held virtually (on Zoom) to allow for easier participation of learners from other campuses; however, we found that participants were less engaged, and discussions were less rich than in sessions held in person, thus we subsequently held all workshops in person. If in-person workshops are not feasible at other institutions, we recommend reviewing participation expectations at the beginning of the workshop. Facilitators at other institutions should also consider intermittent evaluations and may choose to adjust this curriculum to meet local learner, institutional, and policy needs.

A potential limitation to the dissemination and generalizability of our curriculum is that some facilitators and learners may be unable to practice MABs depending on their state policies and subspecialty. This curriculum was developed in a state that permits abortion until fetal viability, does not require parental involvement for minors to access abortion, allows advanced practice practitioners and physicians to provide abortions, and has a shield law to protect abortion providers.^26^ In addition, using the skills learned in our curriculum does require local buy-in and planning from key personnel and stakeholders to start providing MABs; however, the proven safety and efficacy of no-test MABs have significantly lowered implementation barriers, and we have demonstrated elsewhere that offering MABs to primary care patients can be successfully done in pediatric settings.^10,27,28^ We acknowledge that the legality of abortion in other states and countries is highly variable and rapidly shifting. However, to our knowledge it is legal in every state to discuss abortions in an educational curriculum, and we contend that abortion-related education remains invaluable for trainees who may ultimately practice in or refer patients to other locations. To address this limitation, we have included a module on harm-reduction strategies that are applicable regardless of state policies on abortion, provided references to national resources to assist providers in helping their patients access abortion care, and included a prompting slide in the workshop as well as an optional minor consent case question for facilitators to incorporate a review of state, local, and institutional guidelines and policies. An additional limitation may be lack of comfort or experience in MAB provision and/or facilitating a values clarification exercise. To address this limitation, we have included additional resources in the curriculum facilitator guide and encourage facilitators to practice this values clarification exercise with colleagues before facilitating with learners to enhance comfort and preparedness. We have also made supplemental resources available to learners via the addition of a learner resource sheet (Appendix K).

In regard to evaluation, our survey did not demonstrate any measurable change in participants’ values or intentions regarding MAB. We suspect this may partially be the result of a high level of support for providing abortion care at baseline, likely reflecting the local political climate of an institution located in San Francisco, California. In addition, our surveys did not evaluate confidence in MAB provision before curriculum completion as a result of unintentional omission, so we were not able to compare the change in confidence; however, baseline confidence would likely be low given the lack of training opportunities previously available. In addition, our postsurvey data collected information immediately after to 1 week after completing the workshop, so we are unable to comment on long-term knowledge retention. We recommend that future studies assess how MAB knowledge is retained by pediatric learners over longer time points. Further, our response rate was relatively low at 54%, a known challenge within graduate medical education research, which may limit the interpretation of feedback provided.^29^ To increase response rates, we began to send follow-up emails 1 week after completing the workshop encouraging postsurvey completions.

Our curriculum also did not include a practical component in which learners could be involved in real-life MAB provision, which was attributable to the low volume of patients seeking medical abortion in our clinical setting during this time. As noted, we hope that the additional time added to the workshop provides trainees with vicarious learning opportunities to promote confidence in MAB provision while recognizing that confidence (particularly in more advanced topics such as postabortion care) would likely be enhanced with real-life clinical experience.

Future directions include implementation and further evaluation of our curriculum as part of the pediatric residency academic half-day program (which would allow us to target a greater number of learners in one session), training of learners in other specialties who might provide abortion in a primary care setting (e.g., internal medicine), and expansion of trainings to target existing practicing pediatric providers. As our clinical volume of MABs increases, we also hope to incorporate real-life practical experience to reinforce knowledge and skills learned during the modules and workshop. Additionally, we strongly recommend that future investigations assess patient and family preferences for receiving abortion care in pediatric primary care settings. Although this approach is supported by the adult literature,^12,13^ it is critical that we center patient and family voices on this change in clinical practice.

## Appendices


Curriculum Facilitator Guide.docxModule 1 - Pregnancy Options.mp4Module 2 - Medication Abortion Management.mp4Module 3 - Postabortion Care.mp4Module 4 - Harm Reduction Strategies.mp4Workshop Slides.pptxCase.docxCase Facilitator Guide.docxPresurvey.docxPostsurvey.docxMAB Learner Resource Sheet.docx
*All appendices are peer reviewed as integral parts of the Original Publication.*
