# Supplementary material for: A Pediatric- and Adolescent-Focused Medication Abortion Curriculum for Multidisciplinary Trainees
Source: MedEdPORTAL. 2025 Nov 13;21:11553. doi: 10.15766/mep_2374-8265.11553 (PMC12612281; doi:10.15766/mep_2374-8265.11553)
Supplement: Supplementary file 1 — Curriculum Facilitator Guide.docxModule 1 - Pregnancy Options.mp4Module 2 - Medication Abortion Management.mp4Module 3 - Postabortion Care.mp4Module 4 - Harm Reduction Strategies.mp4Workshop Slides.pptxCase.docxCase Facilitator Guide.docxPresurvey.docxPostsurvey.docxMAB Learner Resource Sheet.docx [file mep_2374-8265.11553-s001.zip › H. Case Facilitator Guide.docx]

Appendix H: Medication Abortion Case Facilitator Guide

*This document should be used by facilitators to guide case-based discussion (appendix G)*

**Learning Objectives:**

1. Practice counseling on medication and procedural abortions
2. Describe key concepts in anticipatory guidance for MABs
3. Explain complications of MABs that need further evaluation and intervention.

**Clinical Case:**

One of your patients, 18 yo Daniela (she/her), just found out she is pregnant and would like to have an abortion. Her LMP was 6 weeks ago (confirmed by her period tracking app). ​

- How would you counsel her choosing between MAB and procedural abortion? ​
  - *Encourage learners to practice the actual language they would use*
  - *Start by assessing the patient’s knowledge and/or asking permission to provide more information e.g. “what do you know about the different abortion options?” or “is it okay if I share some information on the different types of abortions?”*
  - *Compare and contrast medication vs procedural abortion*


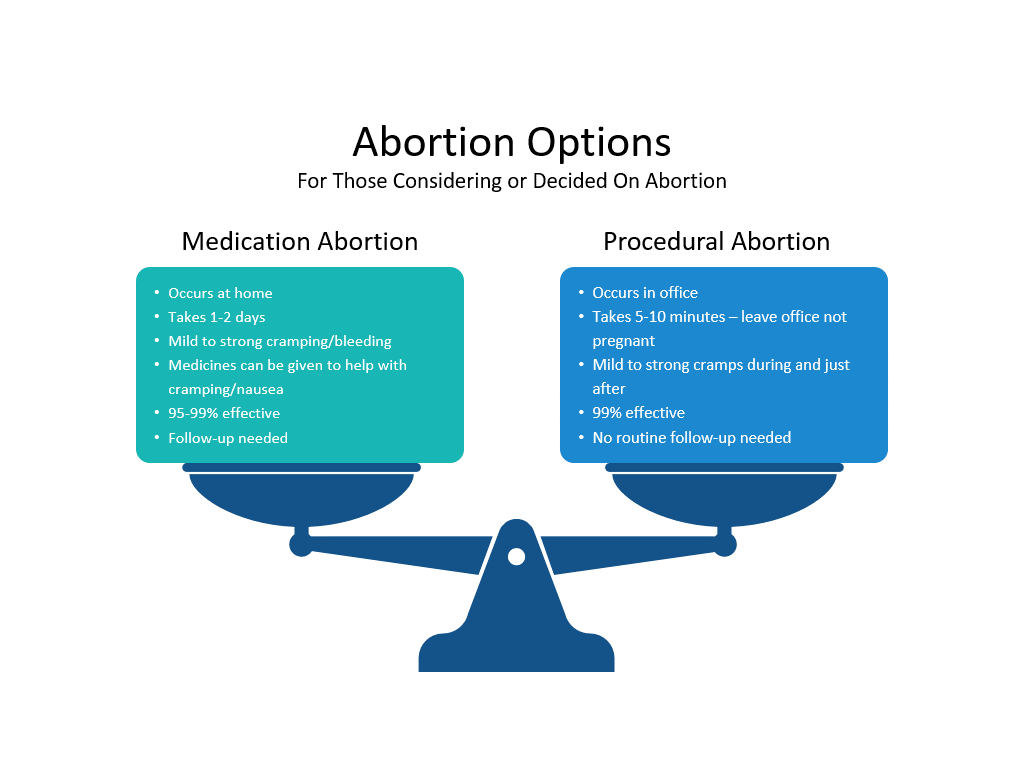


- - *If the patient is unsure, elicit patient preferences around preferred experiences to help guide choice:*
    - *Start with an open-ended question like “what is most important to you about how you experience the abortion?”*
    - *Alleviate fears/worries by eliciting and answering questions*
    - *Assess privacy/confidentiality concerns*
    - *Explore external factors such as school/work schedule and living situation (i.e., access to bathroom)*

She decides to have a medication abortion.​

- What criteria should she meet to be eligible for a no-test medication abortion?


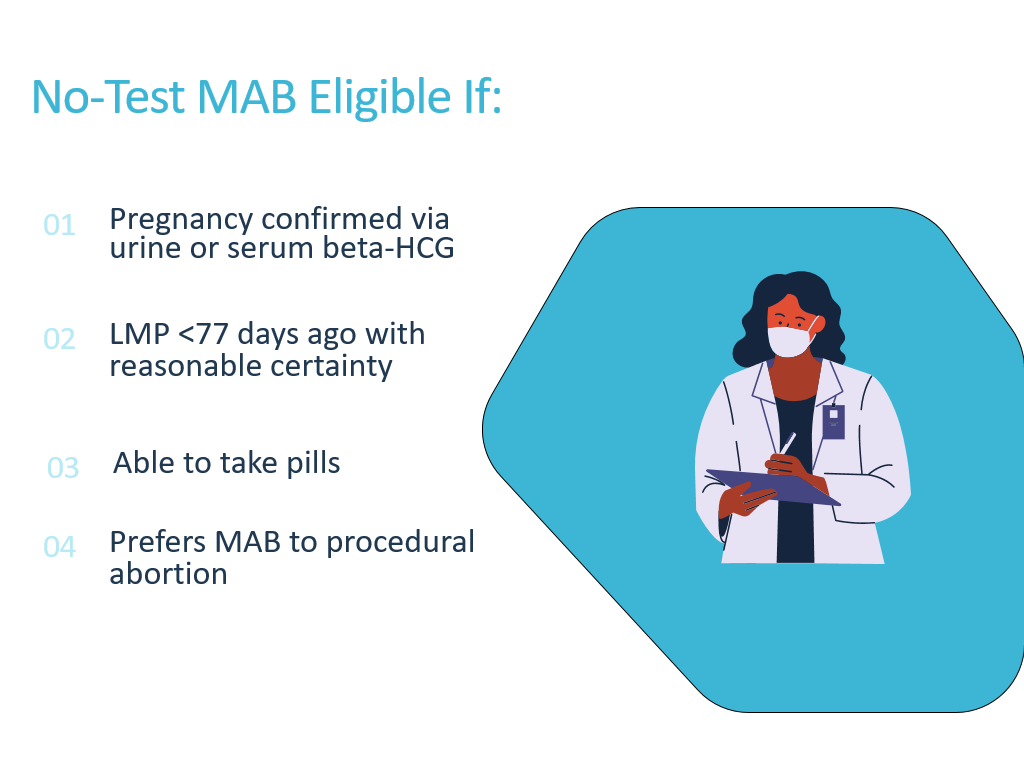


- What are contraindications for no-test MABs?


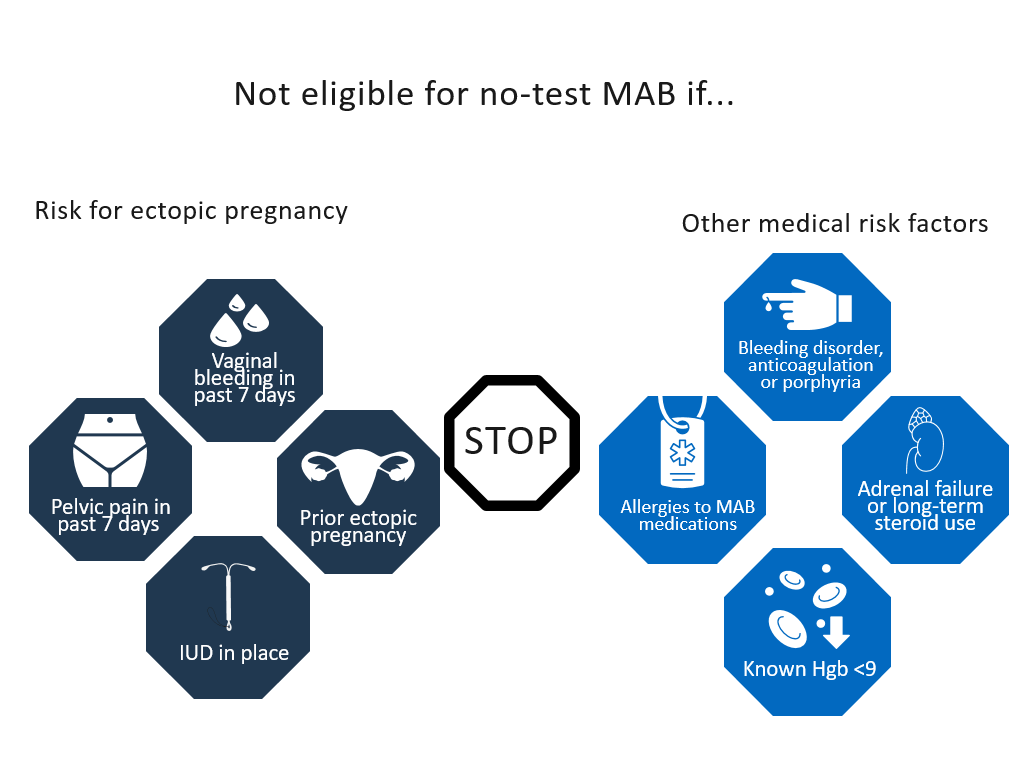


She decides to have a no-test medication abortion. You can give her the medications today. ​

- How would you instruct her to take the medications?​
  - *Again, encourage learners to practice language. Something like this:*
  - *“For the medication abortion, you are going to take two medicines. The first one you’ll take is called mifepristone. The second medicine is called misoprostol - which you’ll take 24-48 hours after the first medicine. The second medicine can be taken by mouth (letting it dissolve in the cheek) or vaginally (you can use your fingers or a tampon applicator to help place the pills in the vagina). We’ll also give you some medicines to help with side effects, including pain and anti-nausea medicines.”*
- How would you counsel her on what to expect after taking the medications? ​
  - *“In the first 24-48 hours after taking the second medication, you will experience heavy bleeding and cramping (like a really heavy period). You may also experience nausea/vomiting/diarrhea, fevers/chills, or headache. The cramping, nausea, and vomiting can be pretty severe, which is why it is important to take the pain and anti-nausea medicines. We recommend you start taking them at the same time as the misoprostol, so you are as comfortable as possible.”*
  - *“In the week following the medications, you will likely continue to experience bleeding and cramping, though this should get better gradually. You may also feel unusually tired. You might have lighter bleeding on and off for up to 4 weeks.”*
  - *“People also sometimes feel a range of emotions after taking the abortion medications including relief, sadness, stress - any emotion that you have is valid and okay to feel. Please let us know if you need any additional support.”*
  - *Note: we recommend sharing a patient handout with learners such as this* [*pictorial handout by RHAP.*](https://www.reproductiveaccess.org/resource/mabfactsheet/)^1^

She calls back with concerns about significant nausea and heavy bleeding 12 hours after taking the misoprostol at home. ​

- What else would you like to know to determine next steps?​
  - *Is she taking the anti-nausea medication every 8 hours? What dose?*
  - *How much bleeding is she having?*
  - *Other symptoms? E.g. severe unilateral abdominal pain? pain that radiates to the shoulder? foul-smelling vaginal discharge?*
- When should she seek emergency care?
  - *Consider consultation with obstetrics/gynecology provider, if available, and/or having her present to emergency care if*
    - *soaking through more than 2 pads per hour for 2 hours in a row*
    - *concerns for ectopic pregnancy*
    - *signs/symptoms of severe dehydration due to nausea/vomiting*

Optional Question (for deeper exploration): ​

- How might this case be different if the patient were 16 (instead of 18) years old in the context of our state, local, and institutional policies?
  - *Can utilize the following resources to guide learners in discussing changes in counseling based on patient age and local laws and policies around confidentiality, consent, and parental notification*
    - [Guttmacher Interactive State Map](https://states.guttmacher.org/policies?)^2^
    - [Guttmacher "Parental Involvement in Minors’ Abortions”](https://www.guttmacher.org/state-policy/explore/parental-involvement-minors-abortions)^3^
    - [AAP Policy Statement](https://publications.aap.org/pediatrics/article/150/3/e2022058780/188339/The-Adolescent-s-Right-to-Confidential-Care-When)^4^
    - [Teen’s Guide to Accessing Abortion](https://www.ineedana.com/blog/a-teen-s-guide-to-accessing-abortion)^5^
  - *Also recommend discussing further about confidentiality piece even in states where parental involvement is not required, for example:*
    - *How do you ensure/support confidentiality if needed/desired? Ideas include getting good contact information, identifying non-parent support person, planning for when/where they will proceed with the MAB*
    - *What can they say to parents if they are bleeding/in pain/need to miss school? Bleeding and cramping from MAB can be similar to a very heavy period so can suggest this as an explanation to parents*
    - *Ensure any relevant documentation is marked confidential*
    - *Identify any insurance concerns (e.g. disclosure via explanation of benefits)*

References:

1. Project RHA. How To Use Abortion Pills Fact Sheet - RHAP. Reproductive Health Access Project. Accessed July 22, 2025. https://www.reproductiveaccess.org/resource/mabfactsheet/

2. Institute G. Interactive Map: US Abortion Policies and Access After Roe. Accessed July 19, 2025. https://states.guttmacher.org/policies/

3. Parental Involvement in Minors’ Abortions | Guttmacher Institute. March 14, 2016. Accessed July 22, 2025. https://www.guttmacher.org/state-policy/explore/parental-involvement-minors-abortions

4. AMERICAN ACADEMY OF PEDIATRICS, COMMITTEE ON ADOLESCENCE. The Adolescent’s Right to Confidential Care When Considering Abortion. *Pediatrics*. 2022;150(3):e2022058780. doi:10.1542/peds.2022-058780

5. A Teen’s Guide to Accessing Abortion | ineedana.com. Accessed July 22, 2025. https://www.ineedana.com/blog/a-teen-s-guide-to-accessing-abortion
