# Supplementary material for: A Pediatric- and Adolescent-Focused Medication Abortion Curriculum for Multidisciplinary Trainees
Source: MedEdPORTAL. 2025 Nov 13;21:11553. doi: 10.15766/mep_2374-8265.11553 (PMC12612281; doi:10.15766/mep_2374-8265.11553)
Supplement: Supplementary file 1 — Curriculum Facilitator Guide.docxModule 1 - Pregnancy Options.mp4Module 2 - Medication Abortion Management.mp4Module 3 - Postabortion Care.mp4Module 4 - Harm Reduction Strategies.mp4Workshop Slides.pptxCase.docxCase Facilitator Guide.docxPresurvey.docxPostsurvey.docxMAB Learner Resource Sheet.docx [file mep_2374-8265.11553-s001.zip › I. Presurvey.docx]

Appendix I: Adolescent Medication Abortion Curriculum Pre-Survey

*This survey can be administered (via paper or online) to learners prior to curriculum completion.*

To create a code to keep your information anonymous, please write the last two digits of your cell phone and your two-day birthday (example: cell phone of xxx-xxx-xx64 and February 13^th^ birthday would be 6413):

**Sociodemographic**

Age

- 21-30
- 31-40
- 41-50
- 51-60
- 60 and above

To which gender identity do you most identify?

- Female
- Male
- Transfemale
- Transmale
- Non-binary/nonconforming
- Prefer to self-describe
- Prefer not to answer

Role

- Pediatric resident
- Psychiatry resident
- NP trainee
- Medical student

If resident or NP trainee: Intended specialty_________________

**Knowledge**

*Policy*

In California, clinicians must inform parents about adolescents’ decision to obtain an abortion

- 1. true **b) false** c) depends on the case

*Eligibility*

Which of the following is NOT a contraindication to medication abortion?

- 1. adrenal insufficiency b) severe anemia (Hg < 10) **c) prior abortion**

d) inherited porphyria

*No-test medication abortion protocol*

Medication abortion (abortion with pills) is safe and routinely performed in the United States until what gestational age?

- 1. 5-6 weeks **b) 10 -11 weeks** c) 15-16 weeks d) 20-21 weeks

All patients should be tested for Rh antigen to prevent the possibility of isoimmunization.

- 1. Yes **b) No**

*Post-abortion care*

Patients should be counseled to call their clinician if they soak through how many pads per hour?

1. **2 maxi-pads per hour for two hours** b) three maxi pads in one hour c) any bleeding after seven days d) three maxi pads for two hours

In the 24 hours after taking misoprostol, which of the clinical scenarios warrants a phone call to a clinician?

1. **no bleeding for 24 hours** b) fever to 100.4 c) nausea and vomiting d) diarrhea

You can start most birth control methods within a few days of your medication abortion

1. **true** b) false

*Harm reduction*

Which of the following is true: self-managed abortions (abortions obtained outside of clinics, most commonly with mifepristone and misoprostol purchased online).

1. Have SIGNFICANT and equal legal and medical risks b) have greater medical than legal risks **c) have greater legal than medical risks**

**Values**

| **The following is a list of reasons that a patient may ask for an abortion. Please indicate whether you agree or disagree (1= Strongly Disagree, 5= Strongly Agree) that the stated reason is morally acceptable.** | | | | | |
| --- | --- | --- | --- | --- | --- |
|  | Strongly disagree | Somewhat disagree | Neutral | Somewhat agree | Strongly agree |
| The patient is financially unable to support the child | 1 | 2 | 3 | 4 | 5 |
| The patient's career/education would be disrupted | 1 | 2 | 3 | 4 | 5 |
| The patient already has too many children | 1 | 2 | 3 | 4 | 5 |
| The pregnancy is a result of rape or incest | 1 | 2 | 3 | 4 | 5 |
| The pregnancy is a threat to the patient's health | 1 | 2 | 3 | 4 | 5 |

| **Please read the following scenario and then indicate your level of agreement with the statement below:**  **A patient who just underwent an abortion and is not interested in starting a birth control method.** | | | | | |
| --- | --- | --- | --- | --- | --- |
|  | Strongly disagree | Somewhat disagree | Neutral | Somewhat agree | Strongly agree |
| I can think of justifiable reasons that would explain why the patient is in this circumstance and makes this decision. | 1 | 2 | 3 | 4 | 5 |
| This case makes me feel frustrated. | 1 | 2 | 3 | 4 | 5 |
| My reaction in this case would make it hard for me to care for the patient. | 1 | 2 | 3 | 4 | 5 |
|  | 1 | 2 | 3 | 4 | 5 |
|  | 1 | 2 | 3 | 4 | 5 |

**Intentions**

|  | Definitely no | Probably no | Unsure | Probably yes | Definitely yes |
| --- | --- | --- | --- | --- | --- |
| I intend to provide medical abortions in my future practice | 1 | 2 | 3 | 4 | 5 |
| I intend to refer future patients medical or surgical abortion | 1 | 2 | 3 | 4 | 5 |
| I intend to advocate for increased access safe, comprehensive abortion services | 1 | 2 | 3 | 4 | 5 |

If you definitely or probably do not plan to offer medication abortions (mark all that apply):

| plan to subspecialize |  |
| --- | --- |
| religious objection |  |
| social stigma |  |
| personal values |  |
| fear of community retaliation |  |
| fear of political climate |  |
| fear of arrest |  |
| fear of personal safety |  |
| fear of litigation |  |
| fear of family safety |  |
| lack of training experiences |  |
| other (free text response). |  |
