# Supplementary material for: A Pediatric- and Adolescent-Focused Medication Abortion Curriculum for Multidisciplinary Trainees
Source: MedEdPORTAL. 2025 Nov 13;21:11553. doi: 10.15766/mep_2374-8265.11553 (PMC12612281; doi:10.15766/mep_2374-8265.11553)
Supplement: Supplementary file 1 — Curriculum Facilitator Guide.docxModule 1 - Pregnancy Options.mp4Module 2 - Medication Abortion Management.mp4Module 3 - Postabortion Care.mp4Module 4 - Harm Reduction Strategies.mp4Workshop Slides.pptxCase.docxCase Facilitator Guide.docxPresurvey.docxPostsurvey.docxMAB Learner Resource Sheet.docx [file mep_2374-8265.11553-s001.zip › K. MAB Learner Resource Sheet.docx]

Appendix K: Medication Abortion Resource Sheet for Learners

*For learners to reference post-curriculum for additional resources and suggested readings*

- Pregnancy options counseling
  - [Options Counseling for the Pregnant Adolescent Patient](https://publications.aap.org/pediatrics/article/150/3/e2022058781/188340/Options-Counseling-for-the-Pregnant-Adolescent?autologincheck=redirected) (article)^1^
  - [Early Abortion Options](https://www.reproductiveaccess.org/wp-content/uploads/2014/12/2022-03-Early-Abortion-Options-1.pdf): created by RHAP (Reproductive Health Access Project)^2^
  - [Pregnancy Options Workbook](https://static1.squarespace.com/static/5fd543bf605f16050e94ab23/t/5fed146a0db4f45ccb4d312b/1609372782775/pregnancy-options-english.pdf)^3^
- MAB provision and post-abortion care
  - [Clear Patient Handout](https://urldefense.com/v3/__https:/www.reproductiveaccess.org/wp-content/uploads/2022/11/2022-09-How-to-Use-Abortion-Pills-Mife-and-Miso_final.pdf__;!!LQC6Cpwp!oqcf3PO3nxYAXf_UK3NmyQSh_taBToUve1l-1fKKlVOqho4gsOEvrd15BAeBaU19VVkCs99K15bZrzBgyjBOEh8nNtA$): created by RHAP^4^
  - [Danco Mifepristone Medication Guide](https://urldefense.com/v3/__https:/www.earlyoptionpill.com/wp-content/uploads/2016/01/DAN_MedGuideEng_FINAL.pdf__;!!LQC6Cpwp!oqcf3PO3nxYAXf_UK3NmyQSh_taBToUve1l-1fKKlVOqho4gsOEvrd15BAeBaU19VVkCs99K15bZrzBgyjBOKiE3K9M$): required to give to patients^5^
  - [No-Test Medication Abortion Protocol](https://urldefense.com/v3/__https:/www.ncbi.nlm.nih.gov/pmc/articles/PMC7161512/__;!!LQC6Cpwp!oqcf3PO3nxYAXf_UK3NmyQSh_taBToUve1l-1fKKlVOqho4gsOEvrd15BAeBaU19VVkCs99K15bZrzBgyjBOICwONCA$) (article)^6^
- Harm reduction & abortion access
  - [Miscarriage and Abortion Hotline](https://urldefense.com/v3/__https:/mahotline.org/__;!!LQC6Cpwp!oqcf3PO3nxYAXf_UK3NmyQSh_taBToUve1l-1fKKlVOqho4gsOEvrd15BAeBaU19VVkCs99K15bZrzBgyjBOAl4Htas$): resource for self-managed abortions^7^
  - [Plan C](https://urldefense.com/v3/__https:/www.plancpills.org/__;!!LQC6Cpwp!oqcf3PO3nxYAXf_UK3NmyQSh_taBToUve1l-1fKKlVOqho4gsOEvrd15BAeBaU19VVkCs99K15bZrzBgyjBOIsZoV_A$): abortion pills by mail in every state^8^
  - [I Need An A](https://www.ineedana.com/): resource to find abortion services^9^
  - [Aid Access](https://urldefense.com/v3/__https:/aidaccess.org/en/__;!!LQC6Cpwp!oqcf3PO3nxYAXf_UK3NmyQSh_taBToUve1l-1fKKlVOqho4gsOEvrd15BAeBaU19VVkCs99K15bZrzBgyjBOjyCws1Y$): legal support to help protect abortion access across all 50 states^10^
  - [Self-Managed Abortions in the US](https://urldefense.com/v3/__https:/jamanetwork.com/journals/jama/article-abstract/2797861__;!!LQC6Cpwp!oqcf3PO3nxYAXf_UK3NmyQSh_taBToUve1l-1fKKlVOqho4gsOEvrd15BAeBaU19VVkCs99K15bZrzBgyjBOJjI9zJE$) (article)^11^
- Legal resources
  - Guttmacher Institute [Interactive State Map](https://states.guttmacher.org/policies?)
  - [Abortion Laws by State](https://reproductiverights.org/maps/abortion-laws-by-state/) (Center for Reproductive Rights)
  - [Lawyering for Reproductive Justice](https://ifwhenhow.org/)
  - [Repro Legal Helpline](https://reprolegalhelpline.org/)
