# Supplementary material for: A Pediatric- and Adolescent-Focused Medication Abortion Curriculum for Multidisciplinary Trainees
Source: MedEdPORTAL. 2025 Nov 13;21:11553. doi: 10.15766/mep_2374-8265.11553 (PMC12612281; doi:10.15766/mep_2374-8265.11553)
Supplement: Supplementary file 1 — Curriculum Facilitator Guide.docxModule 1 - Pregnancy Options.mp4Module 2 - Medication Abortion Management.mp4Module 3 - Postabortion Care.mp4Module 4 - Harm Reduction Strategies.mp4Workshop Slides.pptxCase.docxCase Facilitator Guide.docxPresurvey.docxPostsurvey.docxMAB Learner Resource Sheet.docx [file mep_2374-8265.11553-s001.zip › A. Curriculum Facilitator Guide.docx]

Appendix A: Medication Abortion Curriculum Facilitator Guide

*This document provides an overview and timing of the entire curriculum including supporting references.*

Educational Objectives:

By the end of this activity, learners will be able to:

1. Describe the unique health equity, developmental, and confidentiality considerations related to abortion care for adolescents and young adults (AYAs)
2. Discuss harm reduction strategies to mitigate legal and medical complications for AYAs in abortion-restrictive states
3. Examine values and beliefs related to pregnancy options and abortion care grounded in a reproductive justice framework
4. Apply a no-test medication abortion protocol to a patient case including pregnancy options counseling, eligibility assessment, anticipatory guidance, and post-abortion care

Supplies Needed:

1. Curriculum Facilitator Guide
2. Video Modules
   1. Module One_Pregnacy Options.mp4
   2. Module Two_Medication Abortion Provision.mp4
   3. Module Three_Post-Abortion Care.mp4
   4. Module Four_Harm Reduction Strategies.mp4
3. Workshop Slides
4. Case
5. Case Facilitator Guide
6. Pre & Post-Test

*Unless otherwise noted, all images are licensed under CANVA.

Curriculum Outline:

**Part I: Asynchronous Modules** (~30-40 minutes)

Learners will view four 8-10 minute video modules (appendix B) before participating in the live workshop

**Part II: Live Workshop** (~120 minutes including a 10-minute break) utilizing workshop slides (Appendix C) - can be done in person or via Zoom

1. Introduction (~10 minutes):
   - Brief review of the abortion landscape in the context of reproductive justice and health equity
2. Values Clarification Exercise (~30 minutes):
   - Facilitation of a values clarification exercise intended to explore and clarify learners' values related to pregnancy options and abortion
3. Practical Review of Medication Abortion Provision (~30 minutes):
   - Review of practical content in modules including determination of eligibility, anticipatory guidance, and post-abortion care for medication abortions
4. Case-Based Learning (~30 minutes):
   - Application of learned content to an adolescent patient scenario via case
5. Discussion/Reflection/Wrap-Up (~10 minutes)

Additional Resources/Suggested Readings:

- Pregnancy options counseling
  - [Options Counseling for the Pregnant Adolescent Patient](https://publications.aap.org/pediatrics/article/150/3/e2022058781/188340/Options-Counseling-for-the-Pregnant-Adolescent?autologincheck=redirected) (article)^1^
  - [Early Abortion Options](https://www.reproductiveaccess.org/wp-content/uploads/2014/12/2022-03-Early-Abortion-Options-1.pdf): created by RHAP (Reproductive Health Access Project)^2^
  - [Pregnancy Options Workbook](https://static1.squarespace.com/static/5fd543bf605f16050e94ab23/t/5fed146a0db4f45ccb4d312b/1609372782775/pregnancy-options-english.pdf)^3^
- MAB provision and post-abortion care
  - [Clear Patient Handout](https://urldefense.com/v3/__https:/www.reproductiveaccess.org/wp-content/uploads/2022/11/2022-09-How-to-Use-Abortion-Pills-Mife-and-Miso_final.pdf__;!!LQC6Cpwp!oqcf3PO3nxYAXf_UK3NmyQSh_taBToUve1l-1fKKlVOqho4gsOEvrd15BAeBaU19VVkCs99K15bZrzBgyjBOEh8nNtA$): created by RHAP^4^
  - [Danco Mifepristone Medication Guide](https://urldefense.com/v3/__https:/www.earlyoptionpill.com/wp-content/uploads/2016/01/DAN_MedGuideEng_FINAL.pdf__;!!LQC6Cpwp!oqcf3PO3nxYAXf_UK3NmyQSh_taBToUve1l-1fKKlVOqho4gsOEvrd15BAeBaU19VVkCs99K15bZrzBgyjBOKiE3K9M$): required to give to patients^5^
  - [No-Test Medication Abortion Protocol](https://urldefense.com/v3/__https:/www.ncbi.nlm.nih.gov/pmc/articles/PMC7161512/__;!!LQC6Cpwp!oqcf3PO3nxYAXf_UK3NmyQSh_taBToUve1l-1fKKlVOqho4gsOEvrd15BAeBaU19VVkCs99K15bZrzBgyjBOICwONCA$) (article)^6^
- Harm reduction & abortion access
  - [Miscarriage and Abortion Hotline](https://urldefense.com/v3/__https:/mahotline.org/__;!!LQC6Cpwp!oqcf3PO3nxYAXf_UK3NmyQSh_taBToUve1l-1fKKlVOqho4gsOEvrd15BAeBaU19VVkCs99K15bZrzBgyjBOAl4Htas$): resource for self-managed abortions^7^
  - [Plan C](https://urldefense.com/v3/__https:/www.plancpills.org/__;!!LQC6Cpwp!oqcf3PO3nxYAXf_UK3NmyQSh_taBToUve1l-1fKKlVOqho4gsOEvrd15BAeBaU19VVkCs99K15bZrzBgyjBOIsZoV_A$): abortion pills by mail in every state^8^
  - [I Need An A](https://www.ineedana.com): resource to find abortion services^9^
  - [Aid Access](https://urldefense.com/v3/__https:/aidaccess.org/en/__;!!LQC6Cpwp!oqcf3PO3nxYAXf_UK3NmyQSh_taBToUve1l-1fKKlVOqho4gsOEvrd15BAeBaU19VVkCs99K15bZrzBgyjBOjyCws1Y$): legal support to help protect abortion access across all 50 states^10^
- [Self-Managed Abortions in the US](https://urldefense.com/v3/__https:/jamanetwork.com/journals/jama/article-abstract/2797861__;!!LQC6Cpwp!oqcf3PO3nxYAXf_UK3NmyQSh_taBToUve1l-1fKKlVOqho4gsOEvrd15BAeBaU19VVkCs99K15bZrzBgyjBOJjI9zJE$) (article)^11^
- Legal resources
  - Guttmacher Institute [Interactive State Map](https://states.guttmacher.org/policies?)^12^
  - [Abortion Laws by State](https://reproductiverights.org/maps/abortion-laws-by-state/) (Center for Reproductive Rights)^13^
  - [Lawyering for Reproductive Justice](https://ifwhenhow.org/)^14^
  - [Repro Legal Helpline](https://reprolegalhelpline.org)^15^
- Values clarification resources
  - <https://www.reproductiveaccess.org/resource/values-clarification-workshop/>^16^
  - <https://www.ipas.org/resource/abortion-attitude-transformation-a-values-clarification-toolkit-for-global-audiences/>^17^
  - <https://www.innovating-education.org/2016/03/teaching-professionalism-through-a-case-based-values-clarification/>^18^

1. Hornberger LL, Adolescence CO. Options Counseling for the Pregnant Adolescent Patient. *Pediatrics*. 2017;140(3). doi:10.1542/peds.2017-2274

2. Early Abortion Options. Accessed January 17, 2025. https://www.reproductiveaccess.org/wp-content/uploads/2014/12/2022-03-Early-Abortion-Options-1.pdf

3. Pregnancy Options Workbook. Accessed January 17, 2025. https://static1.squarespace.com/static/5fd543bf605f16050e94ab23/t/5fed146a0db4f45ccb4d312b/1609372782775/pregnancy-options-english.pdf

4. 2022-09-How-to-Use-Abortion-Pills-Mife-and-Miso_final.pdf. Accessed February 4, 2025. https://www.reproductiveaccess.org/wp-content/uploads/2022/11/2022-09-How-to-Use-Abortion-Pills-Mife-and-Miso_final.pdf

5. DAN_MedGuideEng_FINAL.pdf. Accessed February 4, 2025. https://www.earlyoptionpill.com/wp-content/uploads/2016/01/DAN_MedGuideEng_FINAL.pdf

6. Raymond EG, Grossman D, Mark A, et al. Commentary: No-test medication abortion: A sample protocol for increasing access during a pandemic and beyond. *Contraception*. 2020;101(6):361-366. doi:10.1016/j.contraception.2020.04.005

7. Miscarriage + Abortion Hotline. Accessed February 4, 2025. https://mahotline.org/

8. PLAN C: Abortion pills by mail in every state. Accessed February 4, 2025. https://www.plancpills.org/

9. I Need an Abortion | ineedana.com. Accessed February 4, 2025. https://www.ineedana.com

10. Access A. Aid Access. Aid Access. Accessed February 4, 2025. https://aidaccess.org/en/

11. Verma N, Grossman D. Self-Managed Abortion in the United States. *Curr Obstet Gynecol Rep*. 2023;12(2):70-75. doi:10.1007/s13669-023-00354-x

12. Institute G. Interactive Map: US Abortion Policies and Access After Roe. Accessed July 19, 2025. https://states.guttmacher.org/policies/

13. Abortion Laws by State. Center for Reproductive Rights. Accessed July 19, 2025. https://reproductiverights.org/maps/abortion-laws-by-state/

14. If/When/How: Lawyers for Reproductive Justice. https://ifwhenhow.org/. Accessed July 19, 2025. https://ifwhenhow.org/

15. Repro Legal Helpline. https://reprolegalhelpline.org/. Accessed July 19, 2025. https://reprolegalhelpline.org/

16. Values Clarification Workshop. Reproductive Health Access Project. Accessed February 4, 2025. https://www.reproductiveaccess.org/resource/values-clarification-workshop/

17. Abortion attitude transformation: A values clarification toolkit for global audiences - Ipas. Accessed February 4, 2025. https://www.ipas.org/resource/abortion-attitude-transformation-a-values-clarification-toolkit-for-global-audiences/

18. innovating_education. Teaching Professionalism through a Case-based Values Clarification. Innovating Education in Reproductive Health. March 9, 2016. Accessed February 4, 2025. https://www.innovating-education.org/2016/03/teaching-professionalism-through-a-case-based-values-clarification/
