# Supplementary material for: A Pediatric- and Adolescent-Focused Medication Abortion Curriculum for Multidisciplinary Trainees
Source: MedEdPORTAL. 2025 Nov 13;21:11553. doi: 10.15766/mep_2374-8265.11553 (PMC12612281; doi:10.15766/mep_2374-8265.11553)
Supplement: Supplementary file 1 — Curriculum Facilitator Guide.docxModule 1 - Pregnancy Options.mp4Module 2 - Medication Abortion Management.mp4Module 3 - Postabortion Care.mp4Module 4 - Harm Reduction Strategies.mp4Workshop Slides.pptxCase.docxCase Facilitator Guide.docxPresurvey.docxPostsurvey.docxMAB Learner Resource Sheet.docx [file mep_2374-8265.11553-s001.zip › F. Workshop Slides.pptx]

## Slide 1
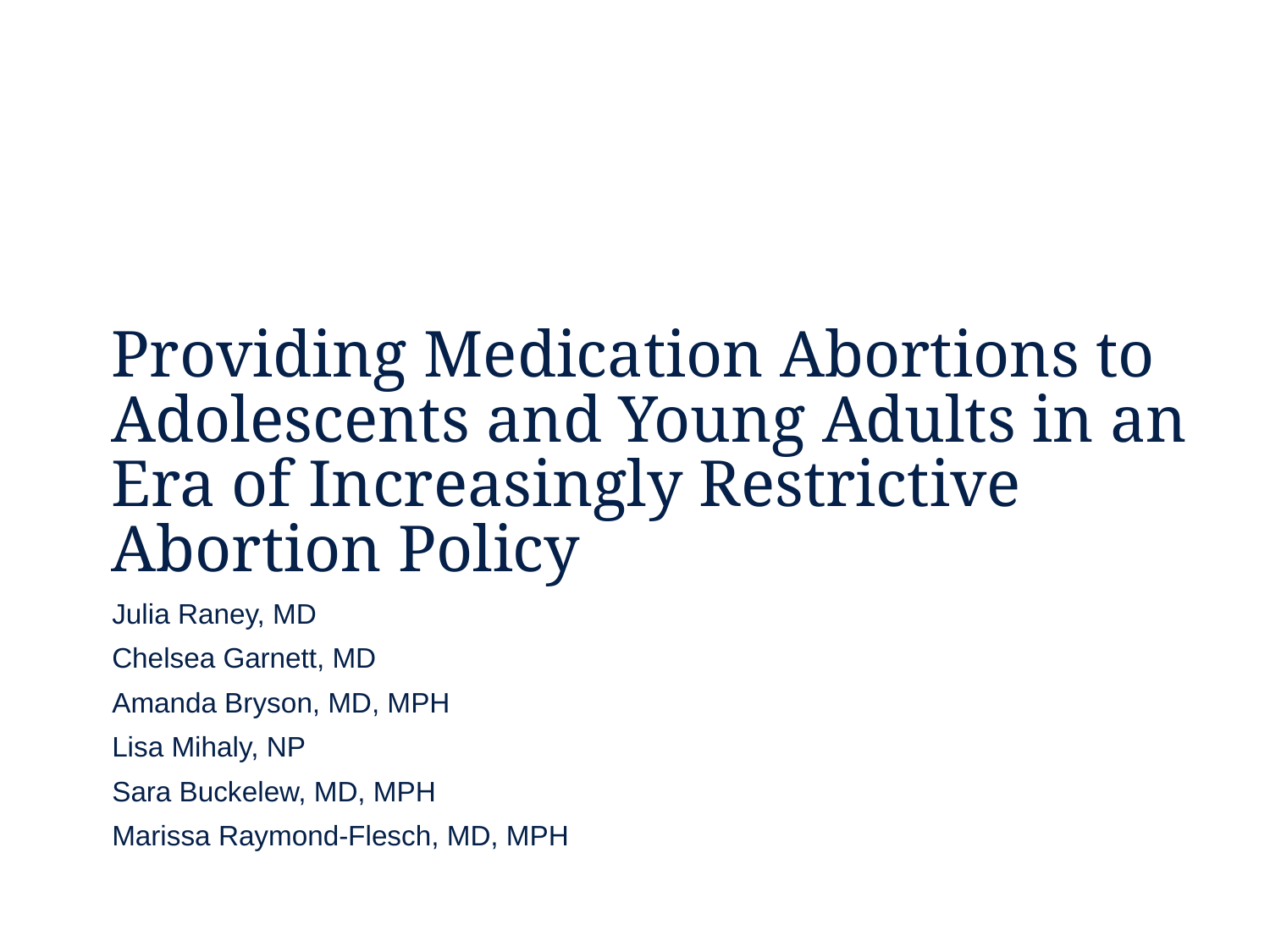

# Providing Medication Abortions to Adolescents and Young Adults in an Era of Increasingly Restrictive Abortion Policy
Julia Raney, MD
Chelsea Garnett, MD
Amanda Bryson, MD, MPH
Lisa Mihaly, NP
Sara Buckelew, MD, MPH
Marissa Raymond-Flesch, MD, MPH

## Slide 2
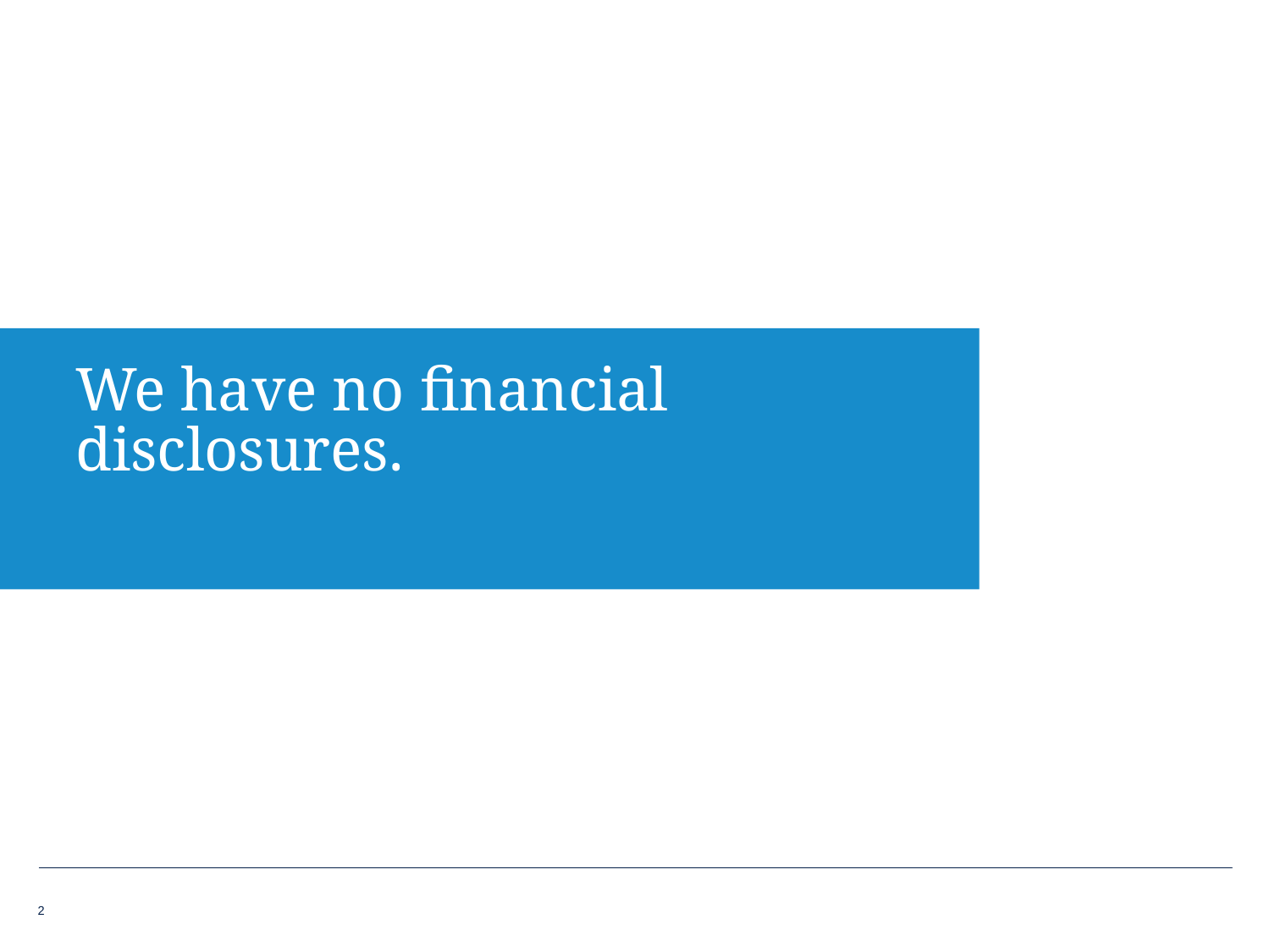

# We have no financial disclosures.
2

## Slide 3
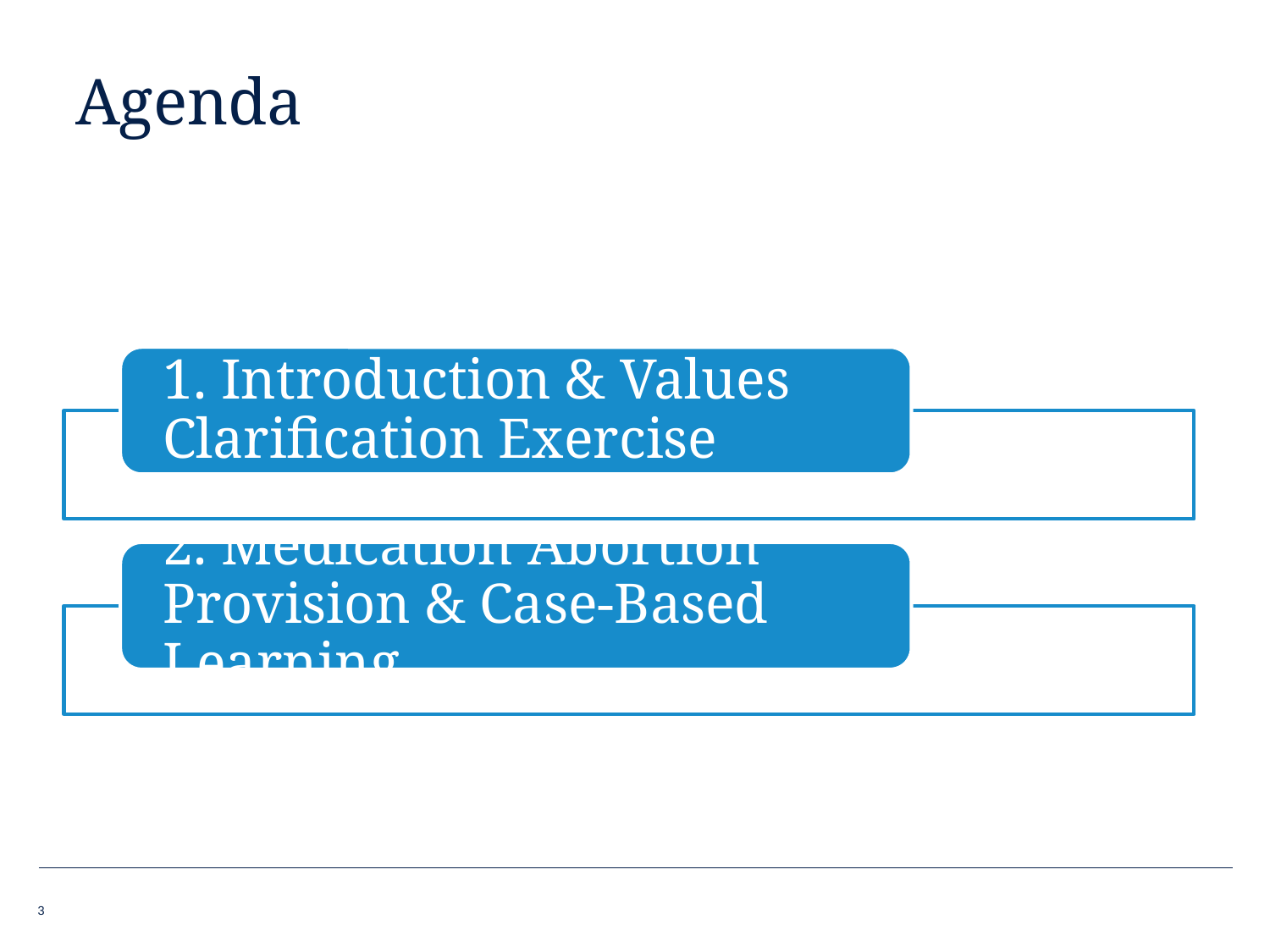

# Agenda
3

## Slide 4
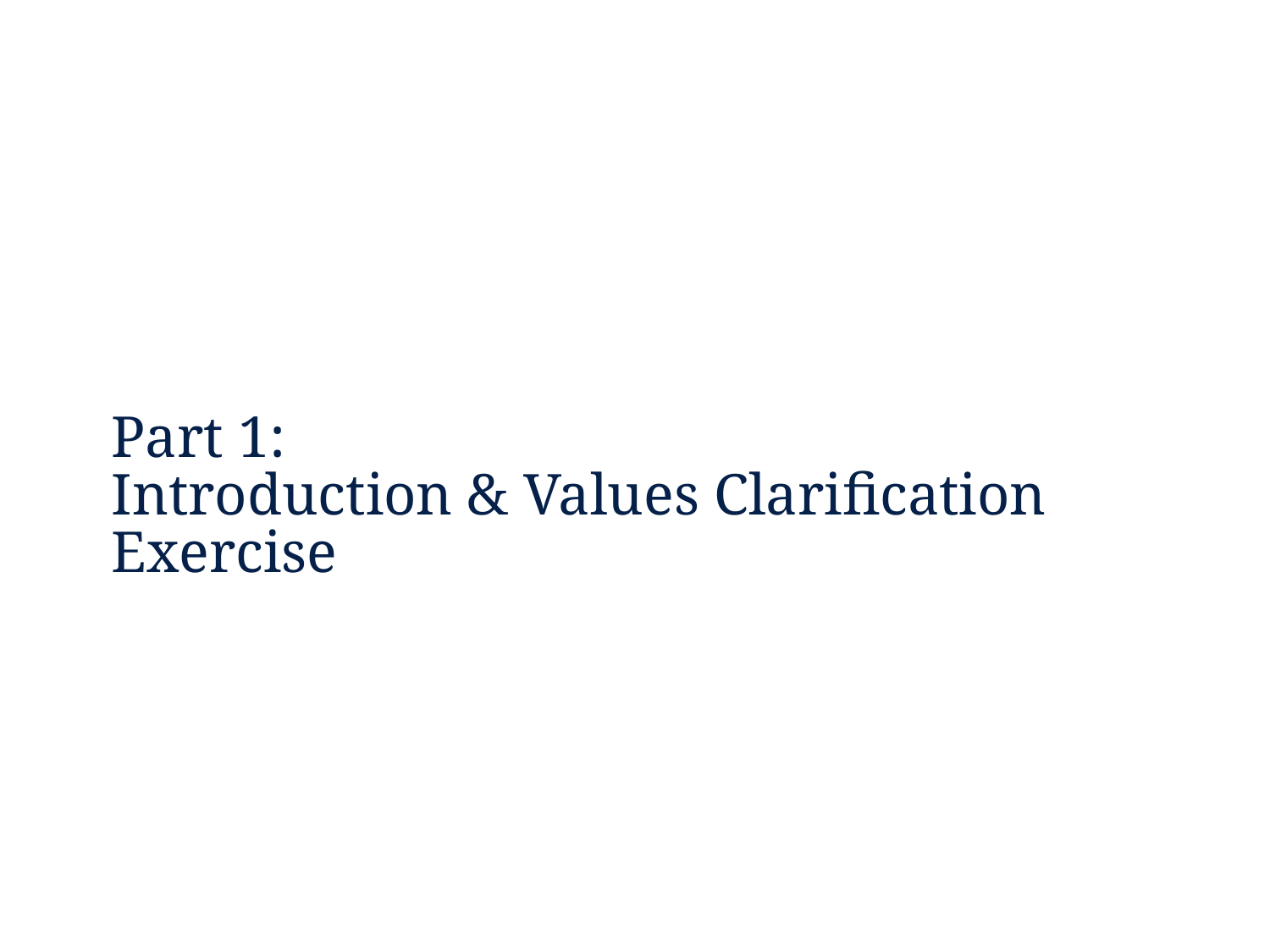

# Part 1: Introduction & Values Clarification Exercise

## Slide 5
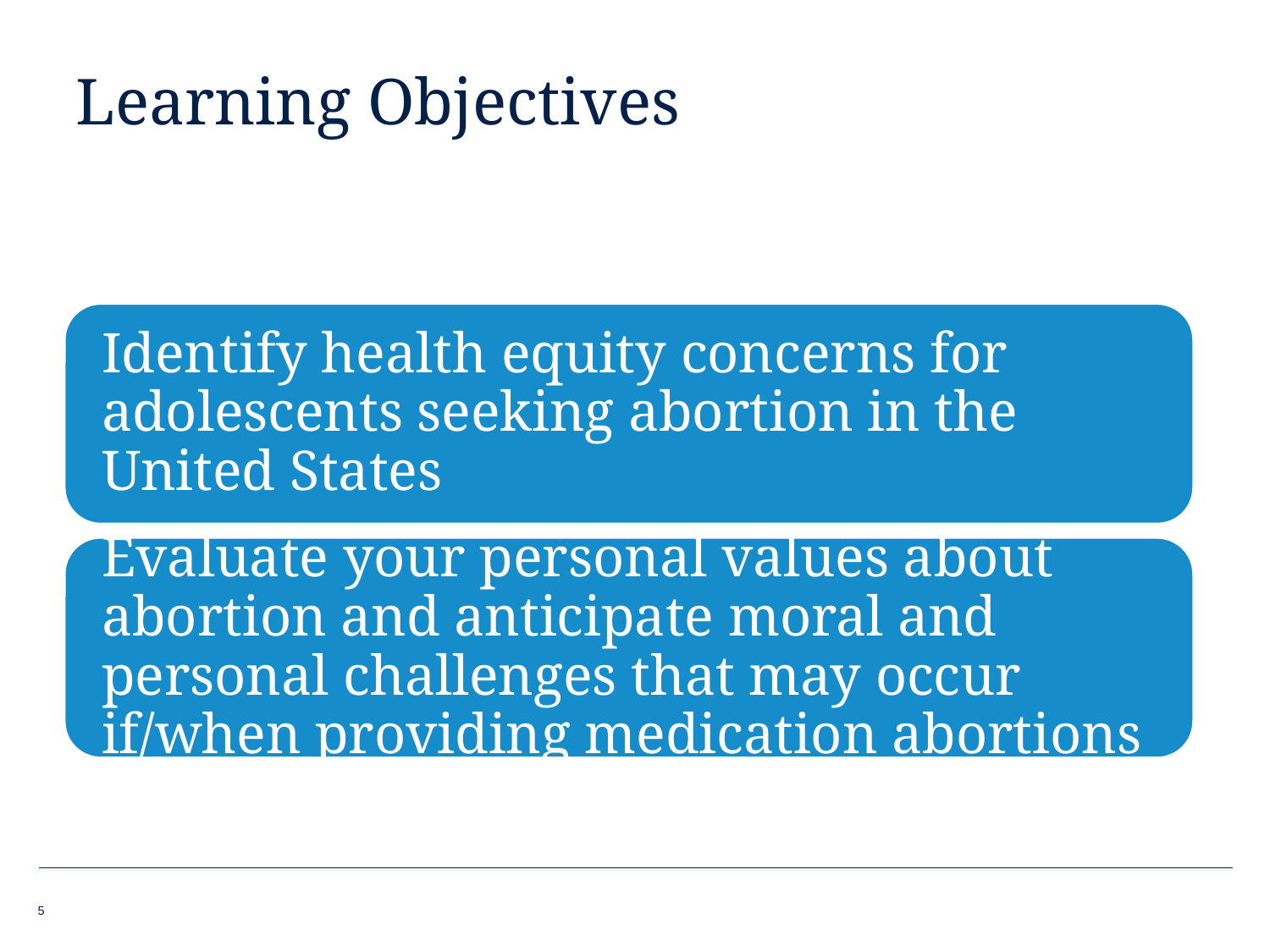

# Learning Objectives
5

## Slide 6
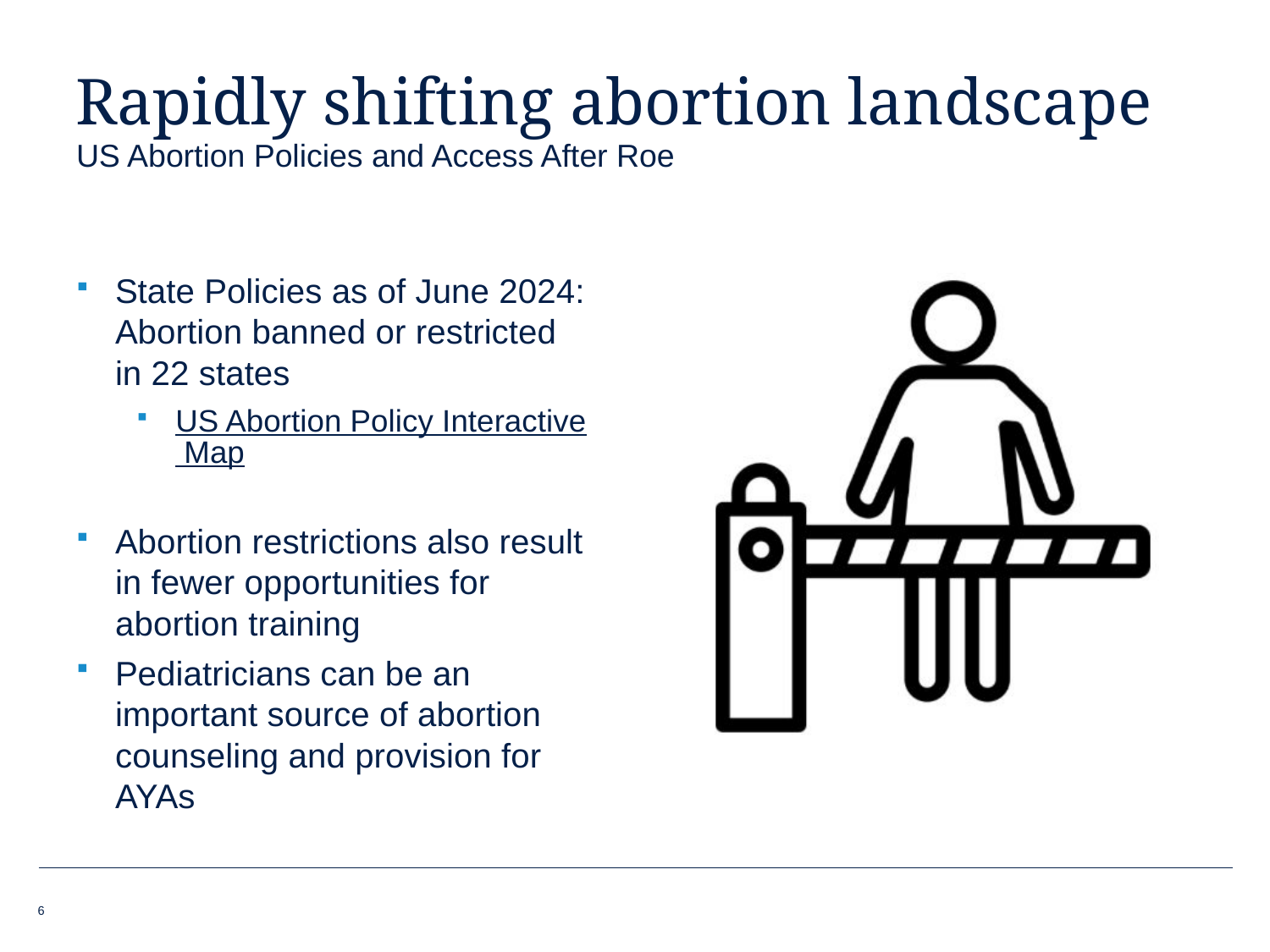

# Rapidly shifting abortion landscape
US Abortion Policies and Access After Roe
State Policies as of June 2024: Abortion banned or restricted in 22 states
US Abortion Policy Interactive Map
Abortion restrictions also result in fewer opportunities for abortion training
Pediatricians can be an important source of abortion counseling and provision for AYAs
6

## Slide 7
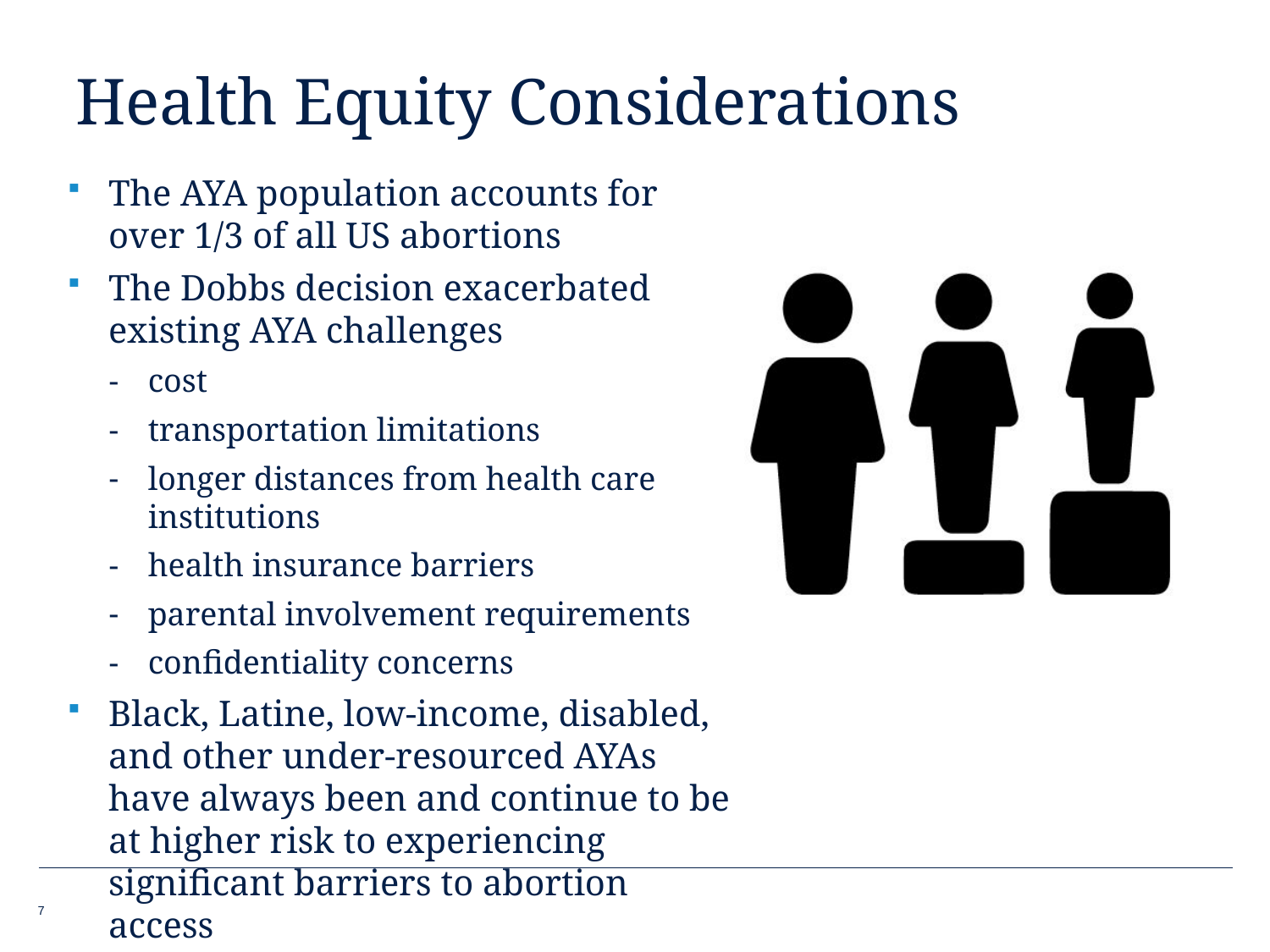

# Health Equity Considerations
The AYA population accounts for over 1/3 of all US abortions
The Dobbs decision exacerbated existing AYA challenges
cost
transportation limitations
longer distances from health care institutions
health insurance barriers
parental involvement requirements
confidentiality concerns
Black, Latine, low-income, disabled, and other under-resourced AYAs have always been and continue to be at higher risk to experiencing significant barriers to abortion access
7

## Slide 8
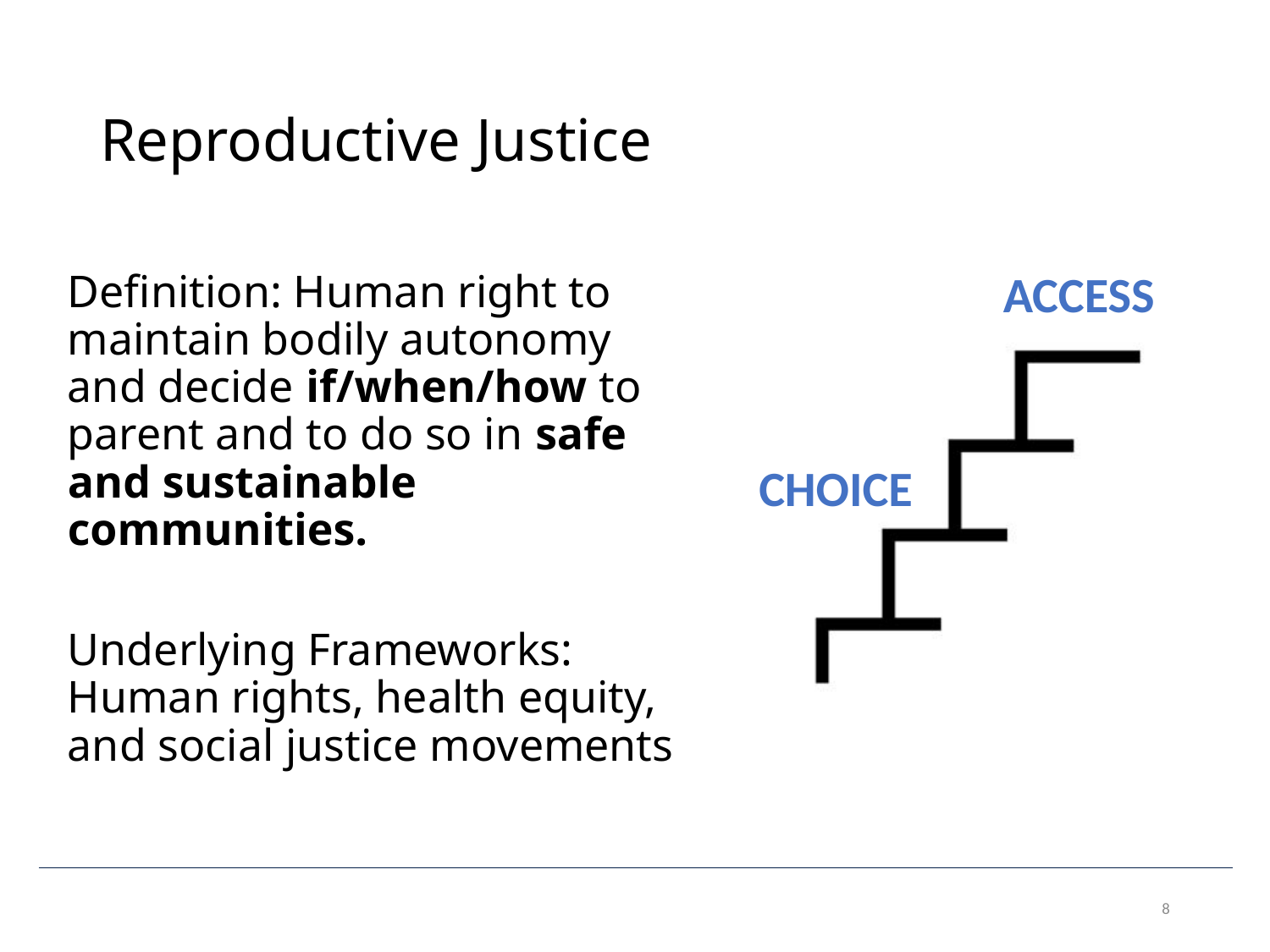

# Reproductive Justice
Definition: Human right to maintain bodily autonomy and decide if/when/how to parent and to do so in safe and sustainable communities.
Underlying Frameworks: Human rights, health equity, and social justice movements
ACCESS
CHOICE
8

## Slide 9
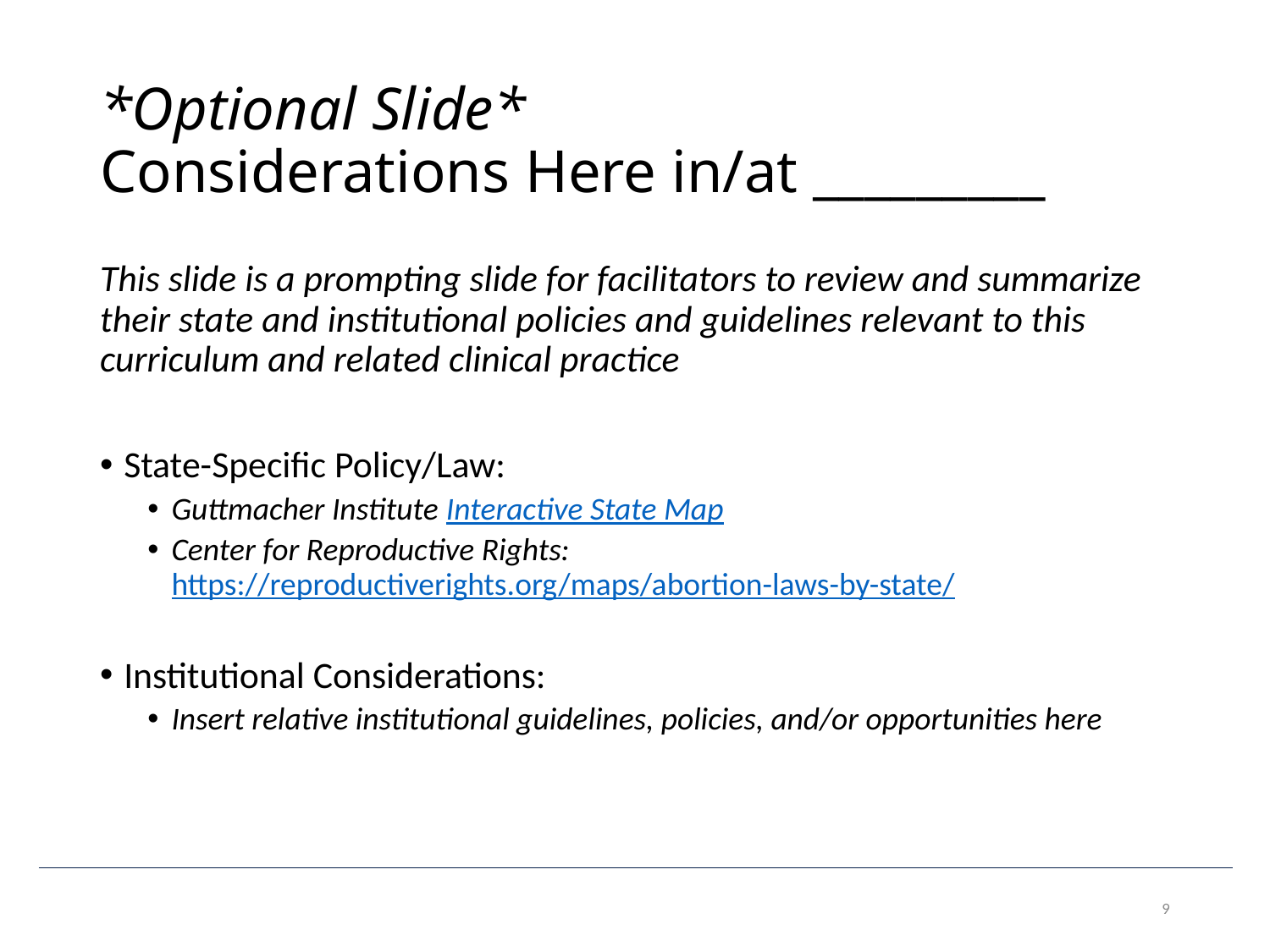

# *Optional Slide* Considerations Here in/at _________
This slide is a prompting slide for facilitators to review and summarize their state and institutional policies and guidelines relevant to this curriculum and related clinical practice
State-Specific Policy/Law:
Guttmacher Institute Interactive State Map
Center for Reproductive Rights: https://reproductiverights.org/maps/abortion-laws-by-state/
Institutional Considerations:
Insert relative institutional guidelines, policies, and/or opportunities here
9

## Slide 10
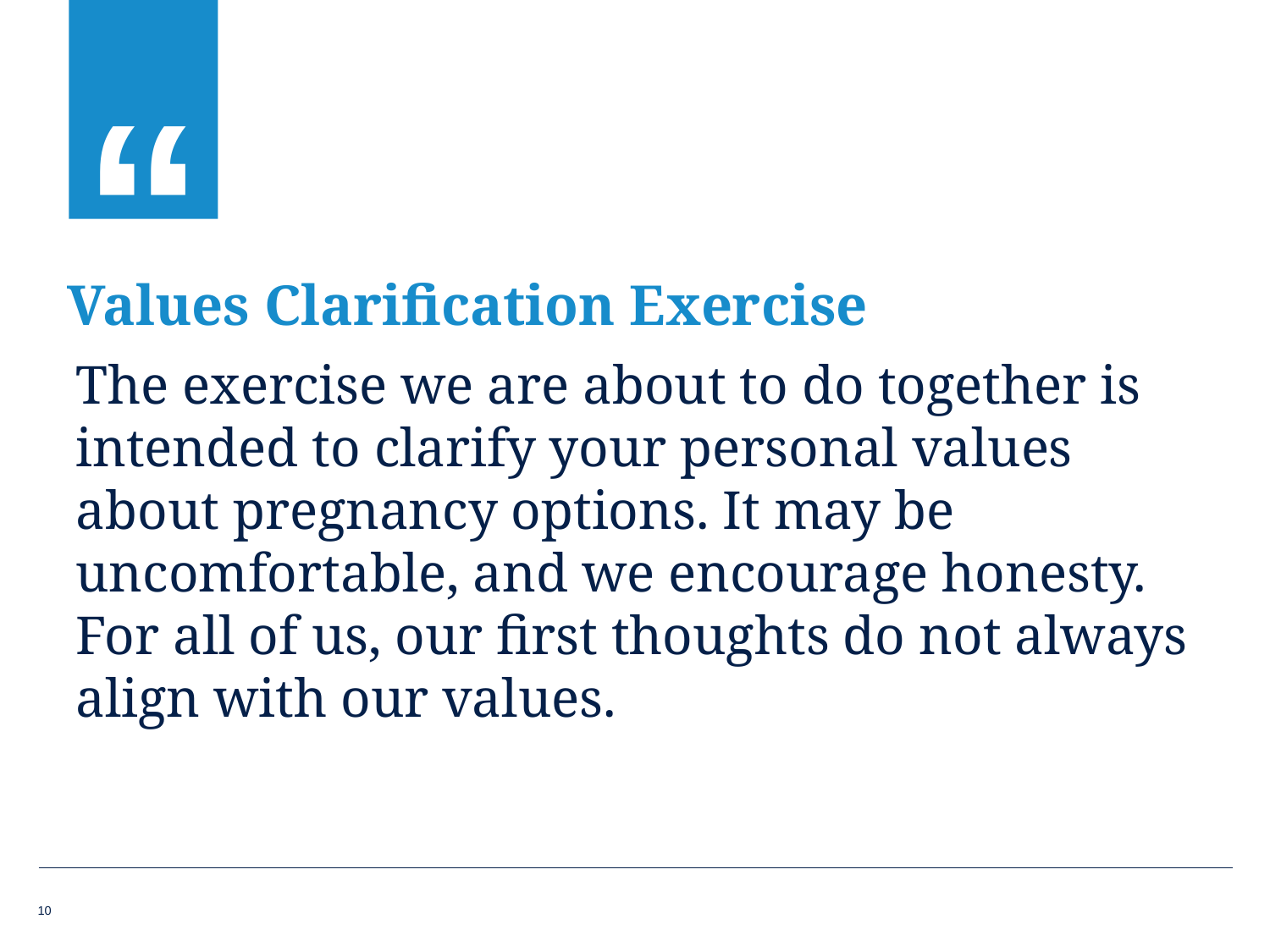

Values Clarification Exercise
The exercise we are about to do together is intended to clarify your personal values about pregnancy options. It may be uncomfortable, and we encourage honesty. For all of us, our first thoughts do not always align with our values.
10

## Slide 11
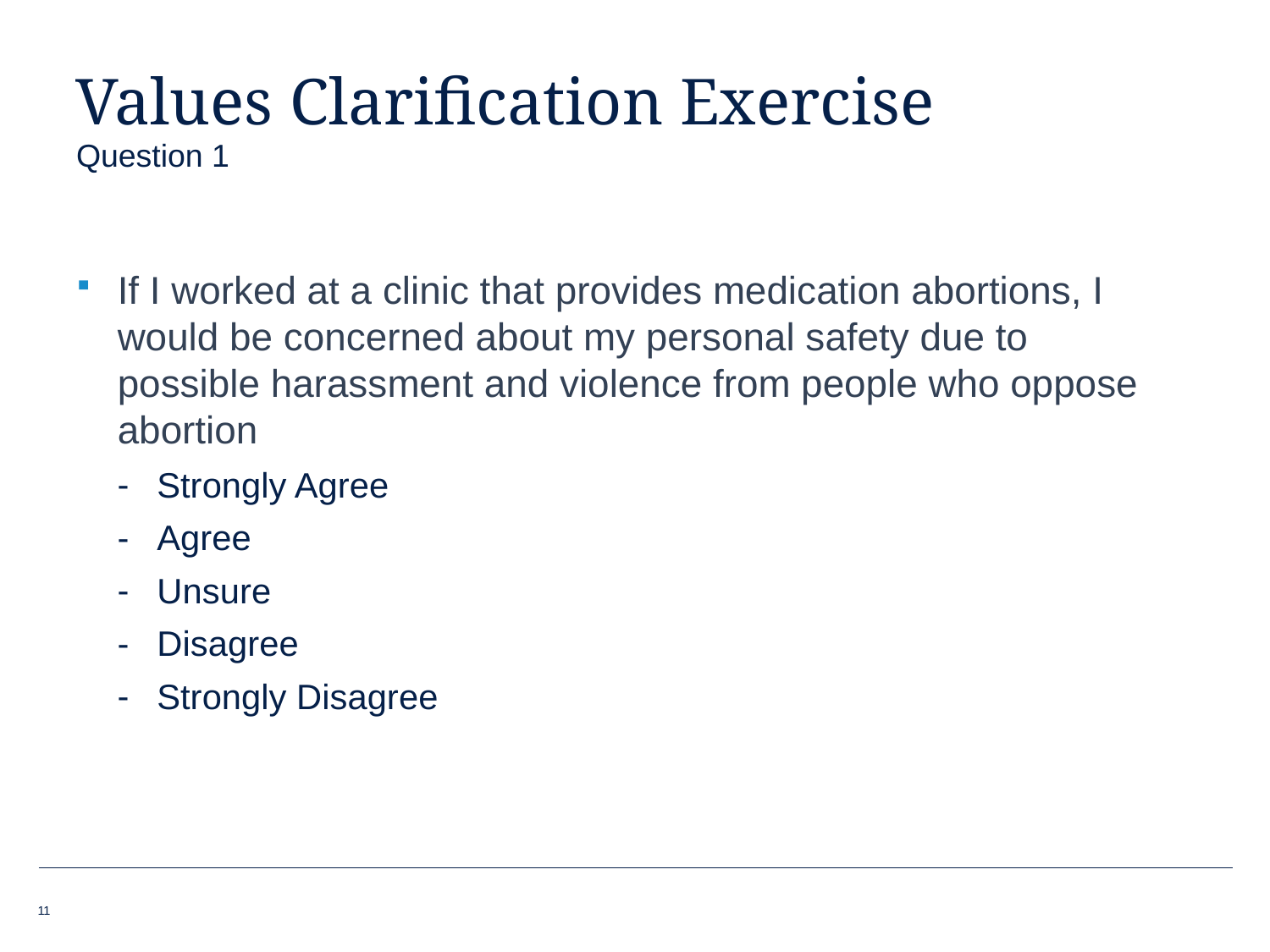

# Values Clarification Exercise
Question 1
If I worked at a clinic that provides medication abortions, I would be concerned about my personal safety due to possible harassment and violence from people who oppose abortion
Strongly Agree
Agree
Unsure
Disagree
Strongly Disagree
11

## Slide 12
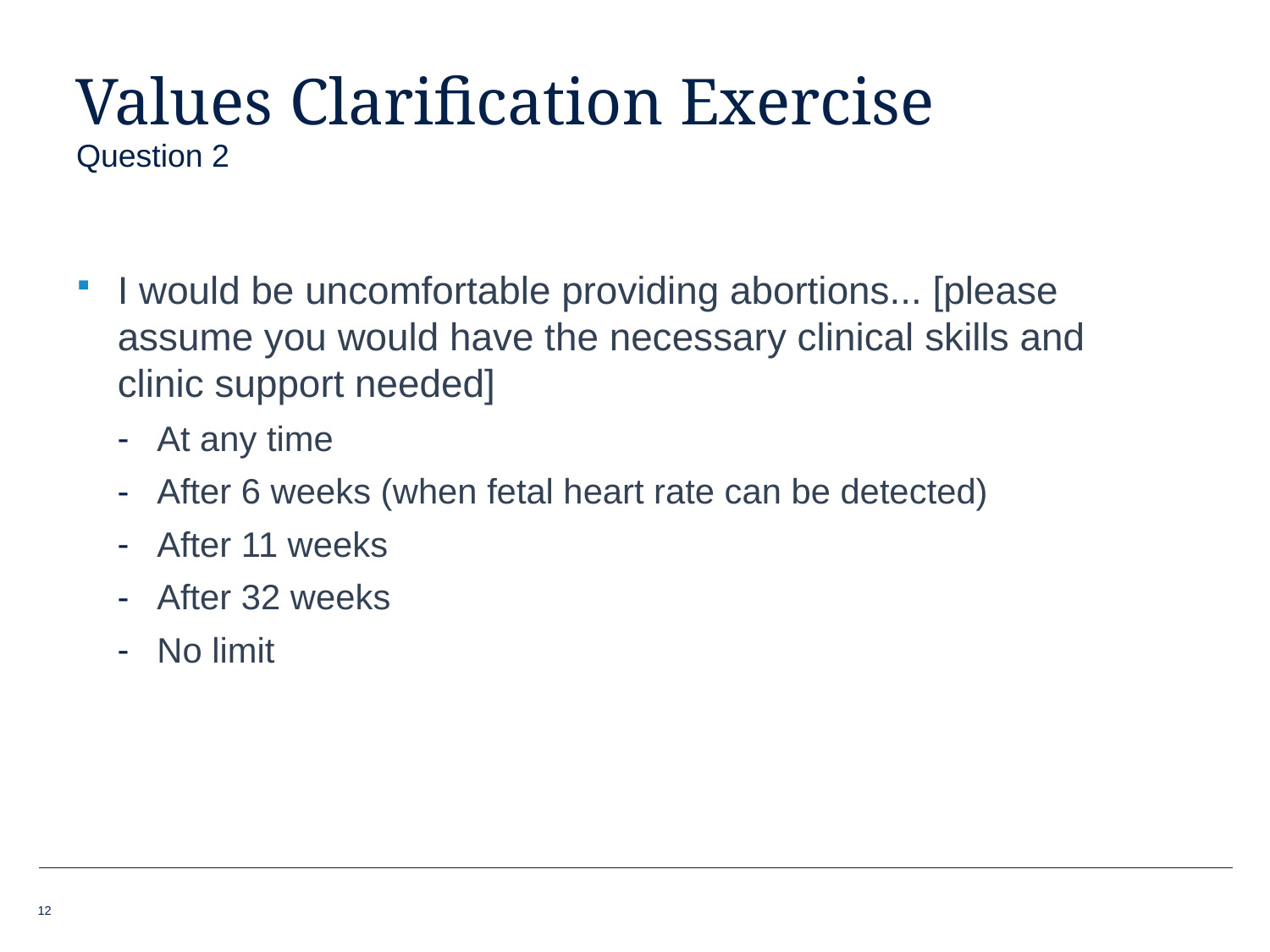

# Values Clarification Exercise
Question 2
I would be uncomfortable providing abortions... [please assume you would have the necessary clinical skills and clinic support needed]
At any time
After 6 weeks (when fetal heart rate can be detected)
After 11 weeks
After 32 weeks
No limit
12

## Slide 13
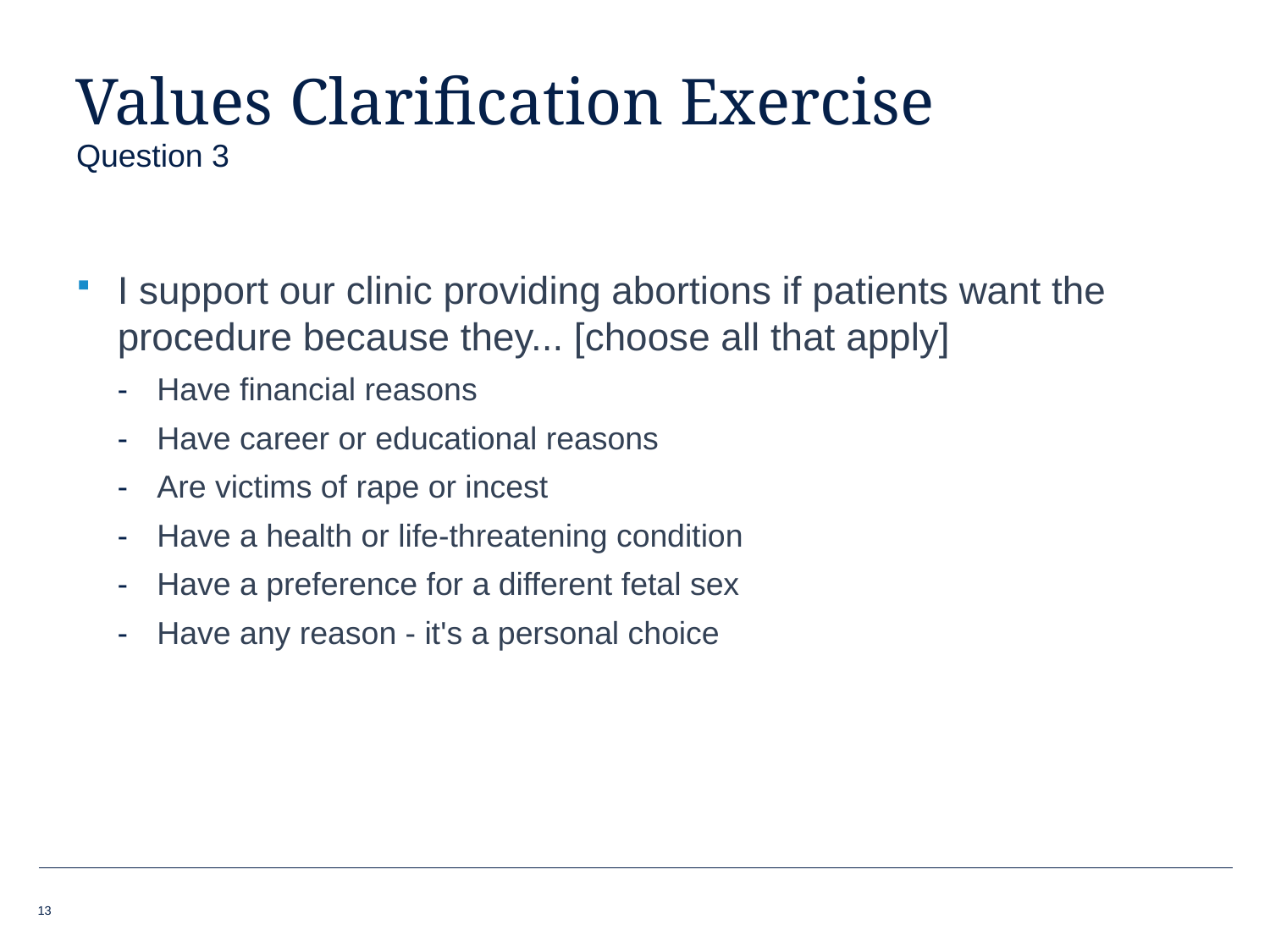

# Values Clarification Exercise
Question 3
I support our clinic providing abortions if patients want the procedure because they... [choose all that apply]
Have financial reasons
Have career or educational reasons
Are victims of rape or incest
Have a health or life-threatening condition
Have a preference for a different fetal sex
Have any reason - it's a personal choice
13

## Slide 14
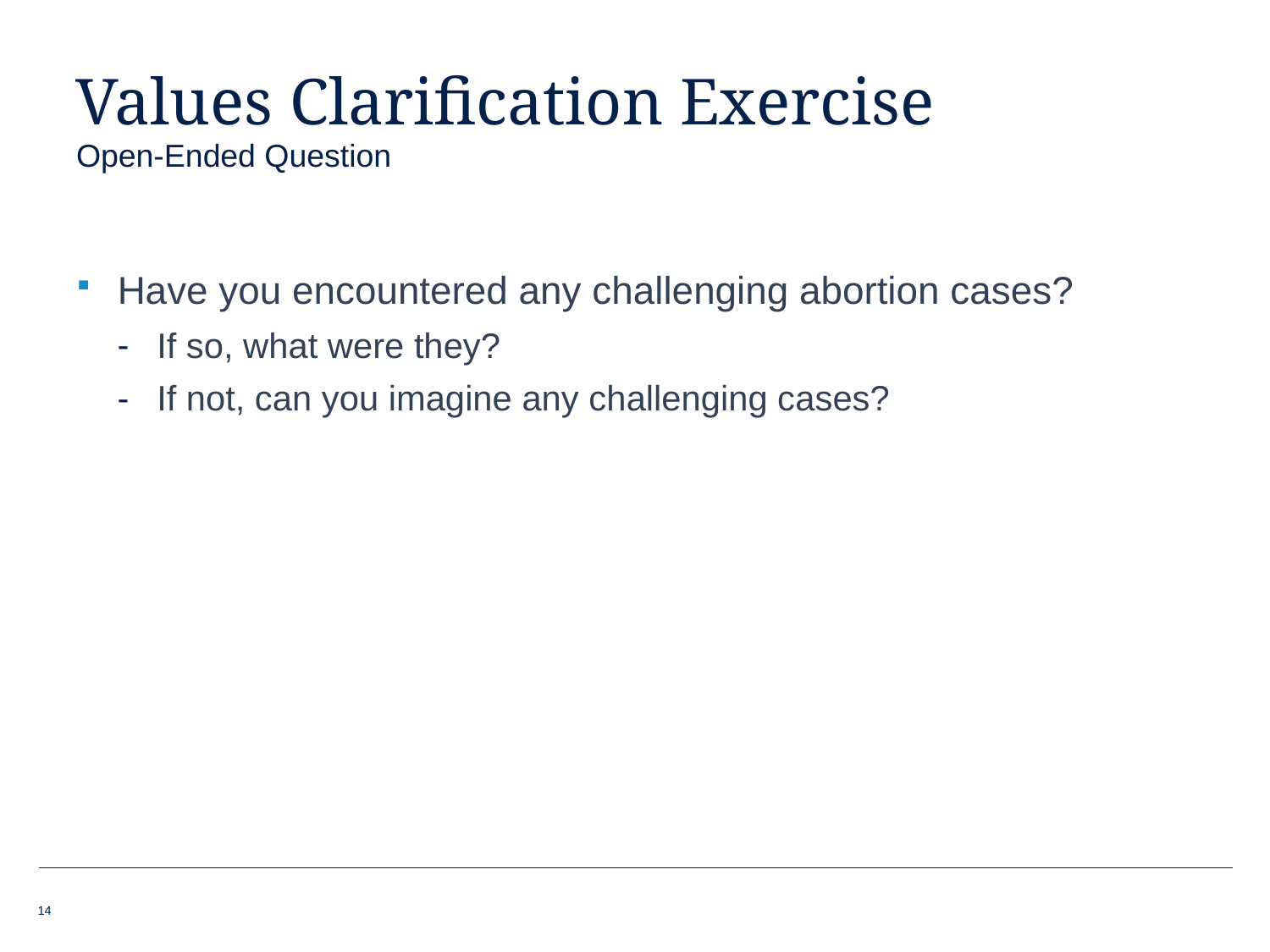

# Values Clarification Exercise
Open-Ended Question
Have you encountered any challenging abortion cases?
If so, what were they?
If not, can you imagine any challenging cases?
14

## Slide 15
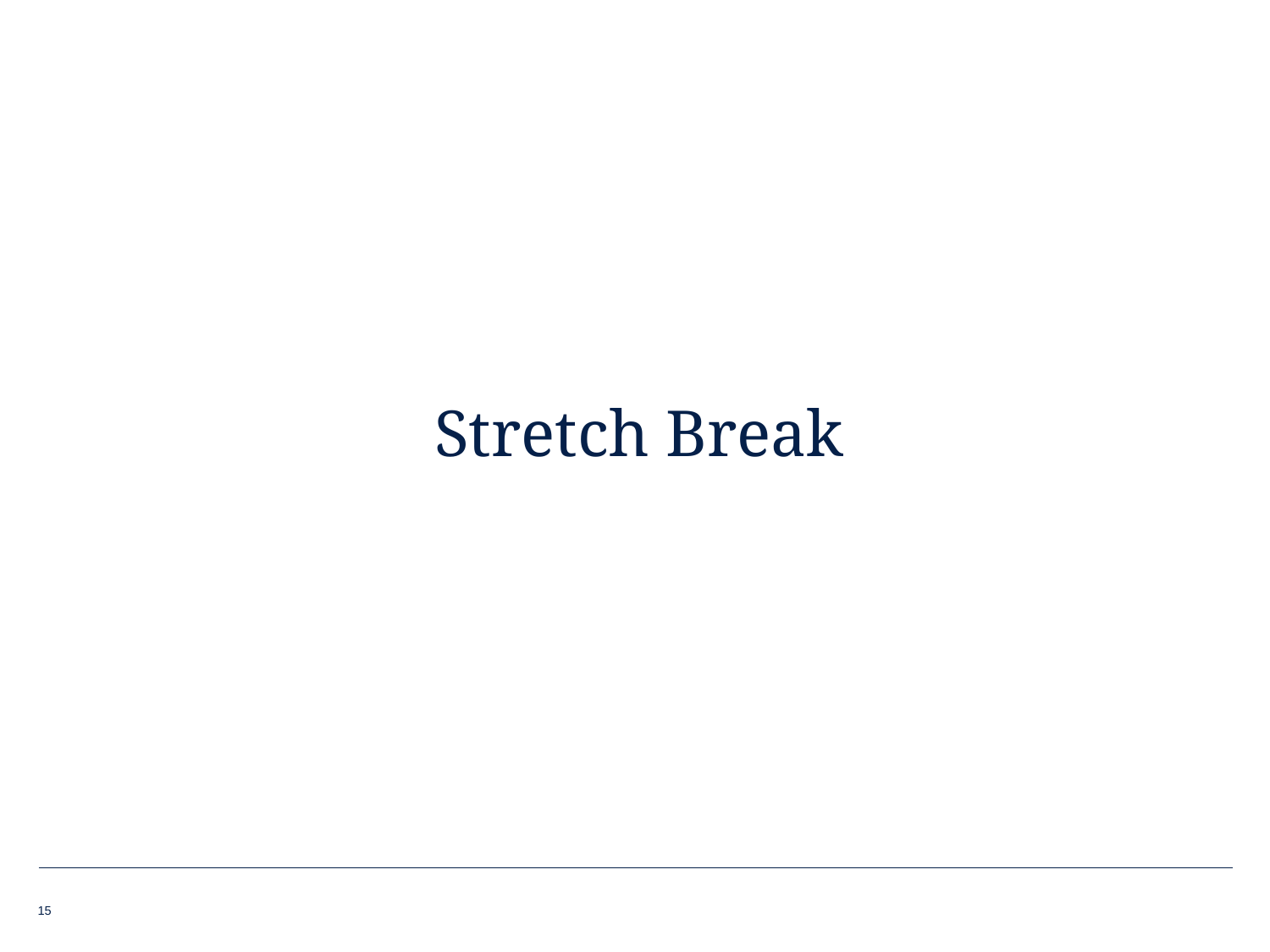

# Stretch Break
15

## Slide 16
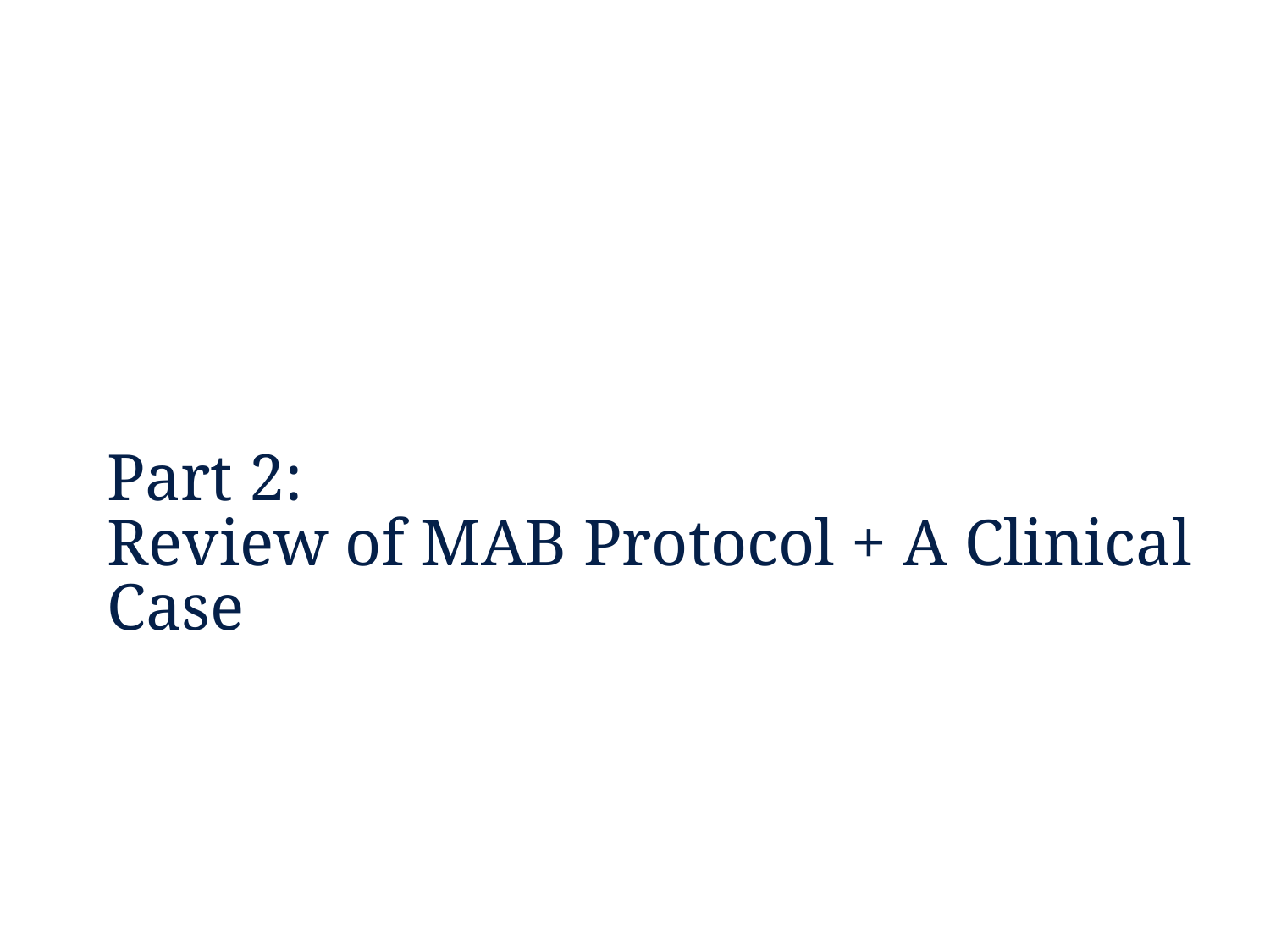

# Part 2: Review of MAB Protocol + A Clinical Case

## Slide 17
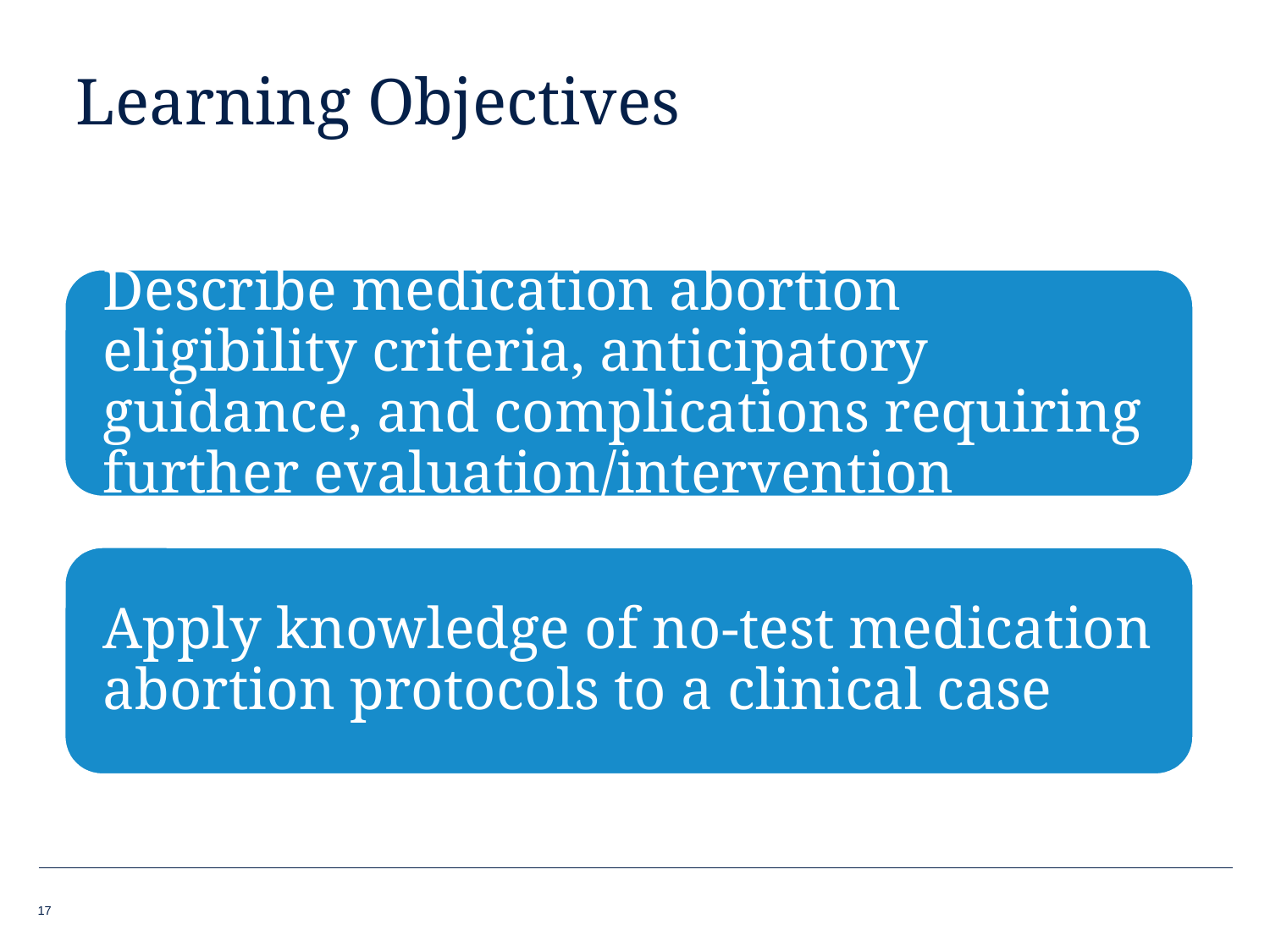

# Learning Objectives
17

## Slide 18
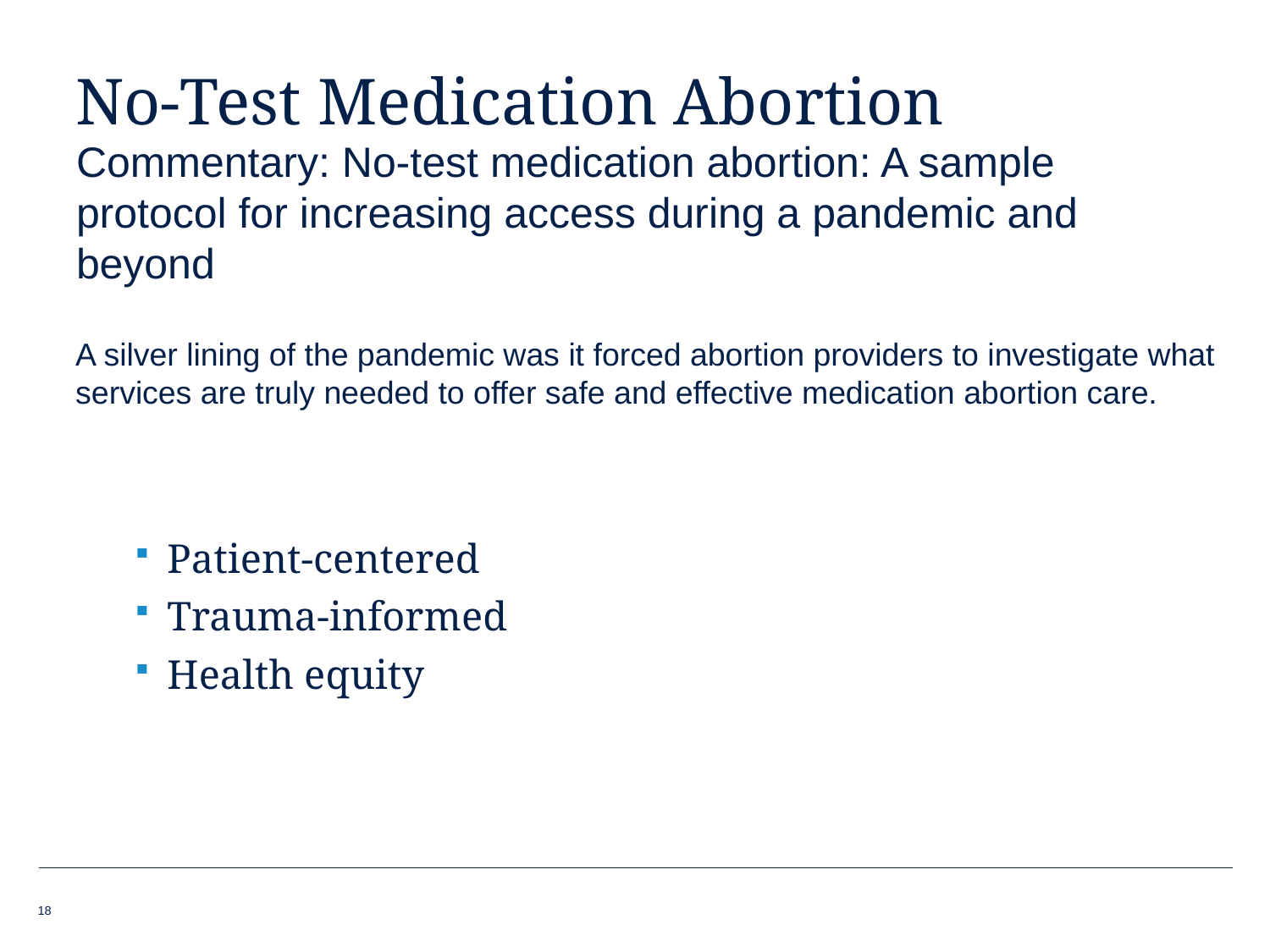

# No-Test Medication Abortion
Commentary: No-test medication abortion: A sample protocol for increasing access during a pandemic and beyond
A silver lining of the pandemic was it forced abortion providers to investigate what services are truly needed to offer safe and effective medication abortion care.
Patient-centered
Trauma-informed
Health equity
18

## Slide 19
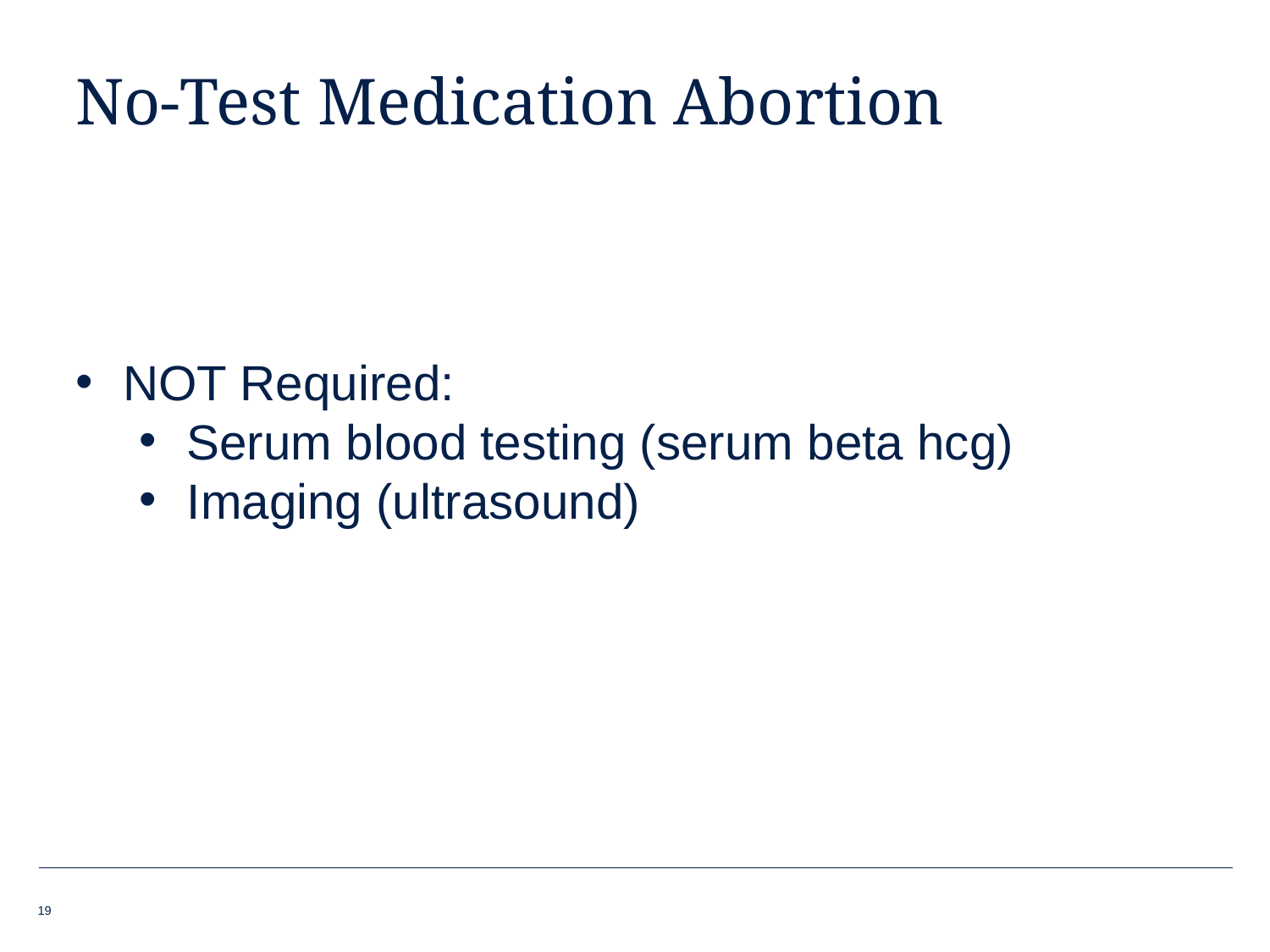

# No-Test Medication Abortion
NOT Required:
Serum blood testing (serum beta hcg)
Imaging (ultrasound)
19

## Slide 20
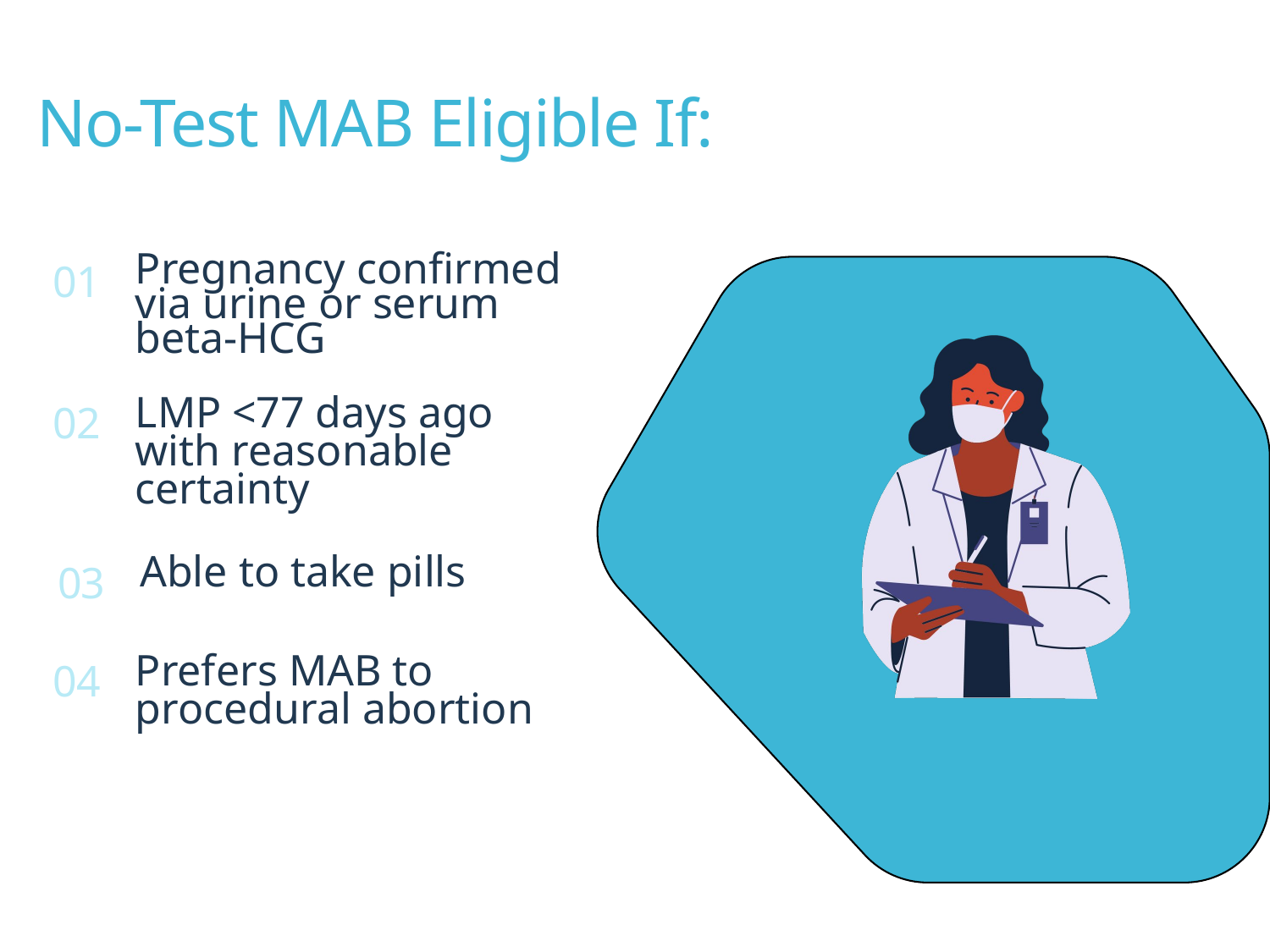

No-Test MAB Eligible If:
Back to Agenda Page
01
Pregnancy confirmed via urine or serum beta-HCG
02
LMP <77 days ago with reasonable certainty
03
Able to take pills
04
Prefers MAB to procedural abortion

## Slide 21
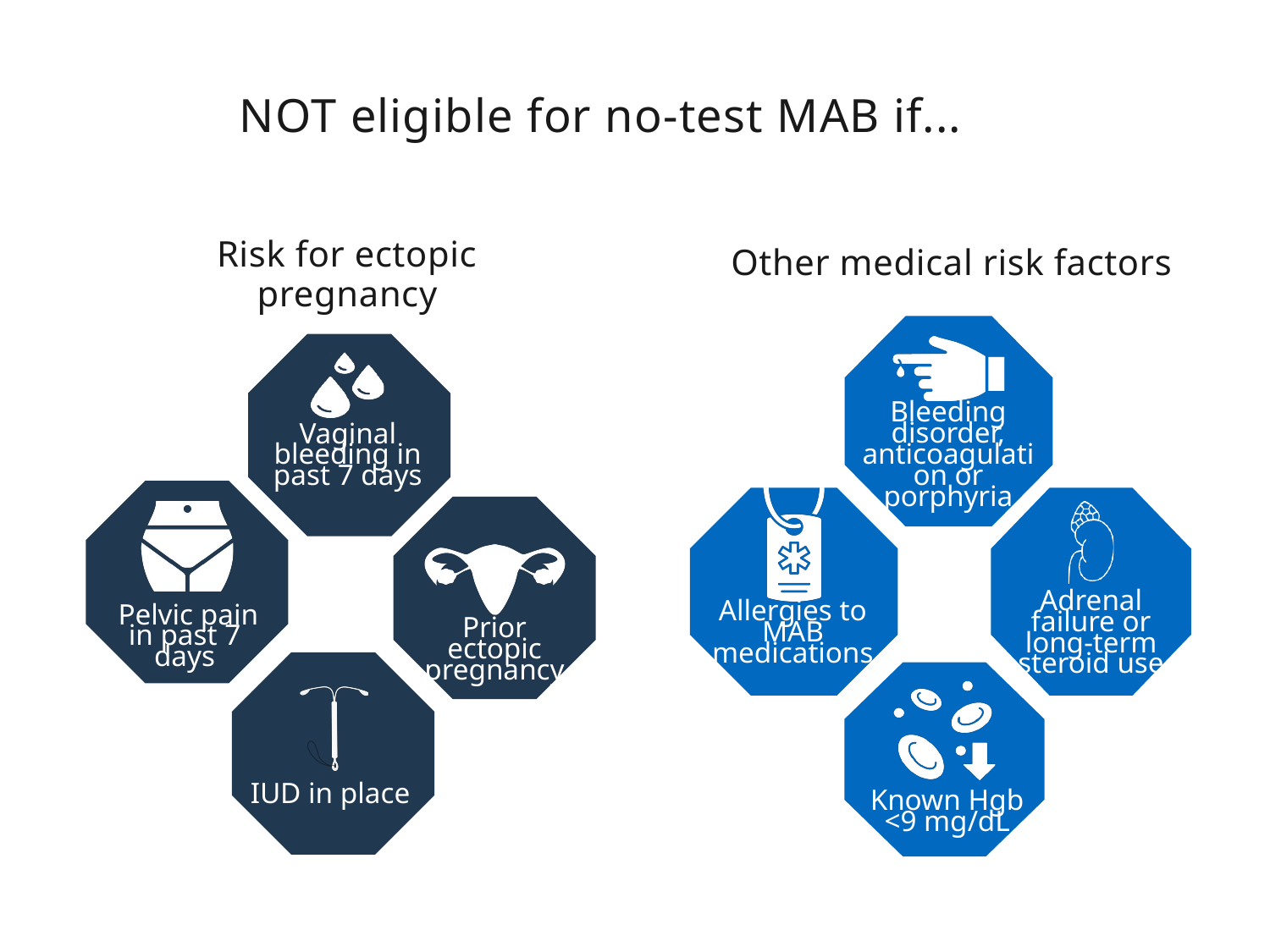

NOT eligible for no-test MAB if...
Risk for ectopic pregnancy
Other medical risk factors
Bleeding disorder,
anticoagulation or porphyria
Vaginal bleeding in past 7 days
 Pelvic pain in past 7 days
Allergies to MAB medications
Adrenal failure or long-term steroid use
Prior ectopic pregnancy
IUD in place
Known Hgb <9 mg/dL

## Slide 22
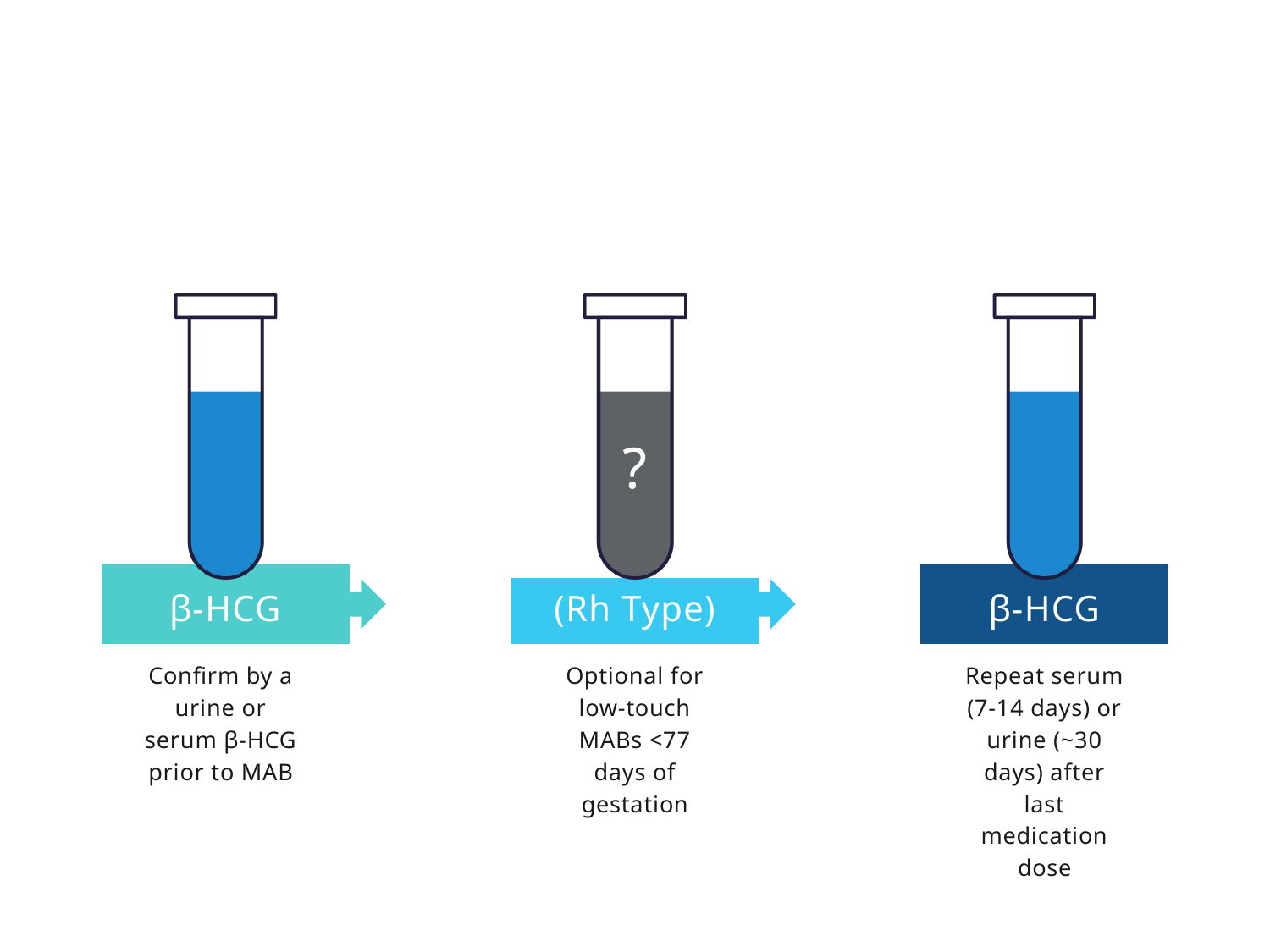

?
β-HCG
(Rh Type)
β-HCG
Confirm by a urine or serum β-HCG prior to MAB
Optional for low-touch MABs <77 days of gestation
Repeat serum (7-14 days) or urine (~30 days) after last medication dose

## Slide 23
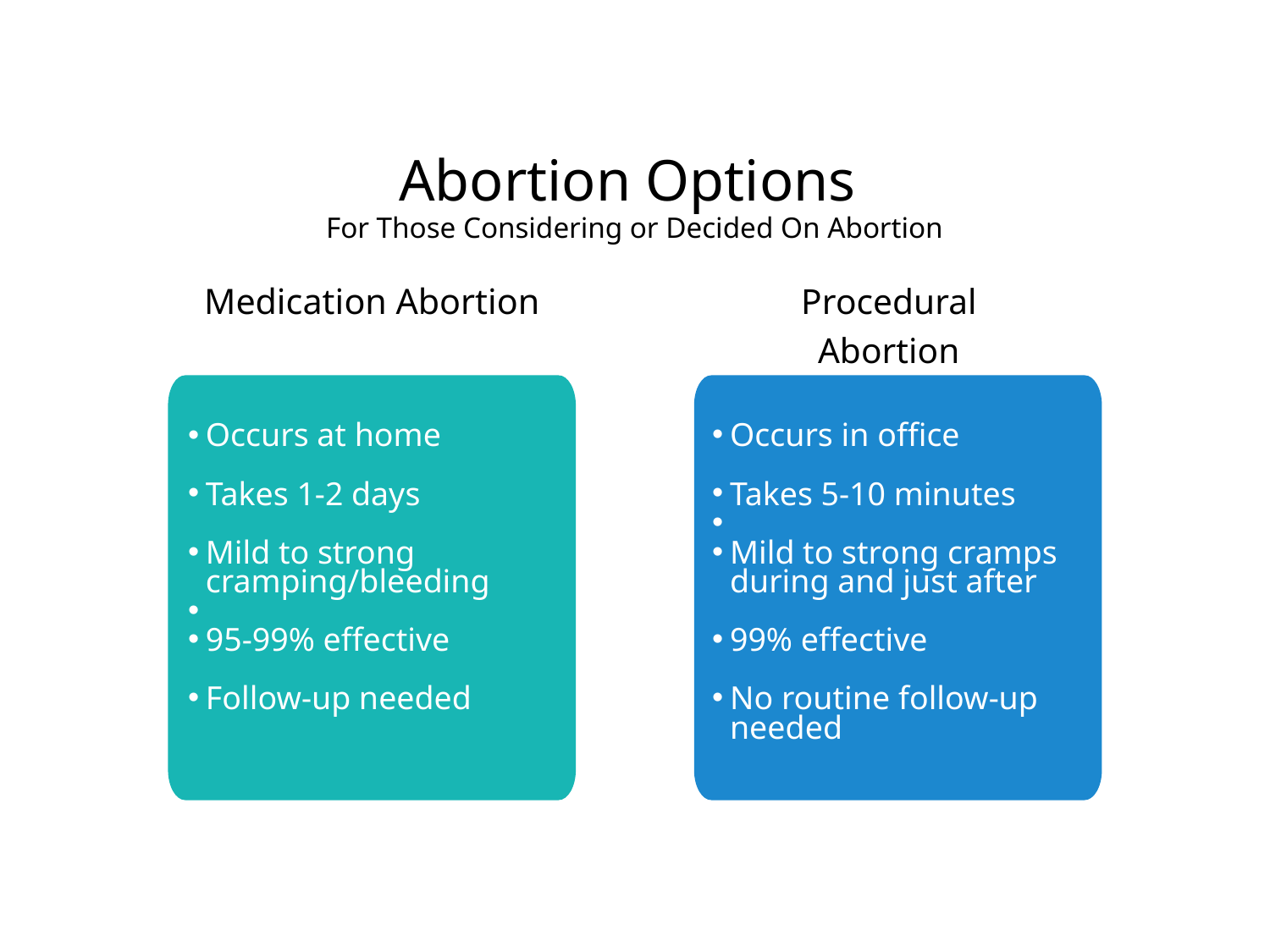

Abortion Options
For Those Considering or Decided On Abortion​
Medication Abortion
Procedural Abortion
Occurs at home ​
Takes 1-2 days​
Mild to strong cramping/bleeding
95-99% effective
Follow-up needed
Occurs in office​
Takes 5-10 minutes
Mild to strong cramps during and just after​
99% effective​
No routine follow-up needed​

## Slide 24
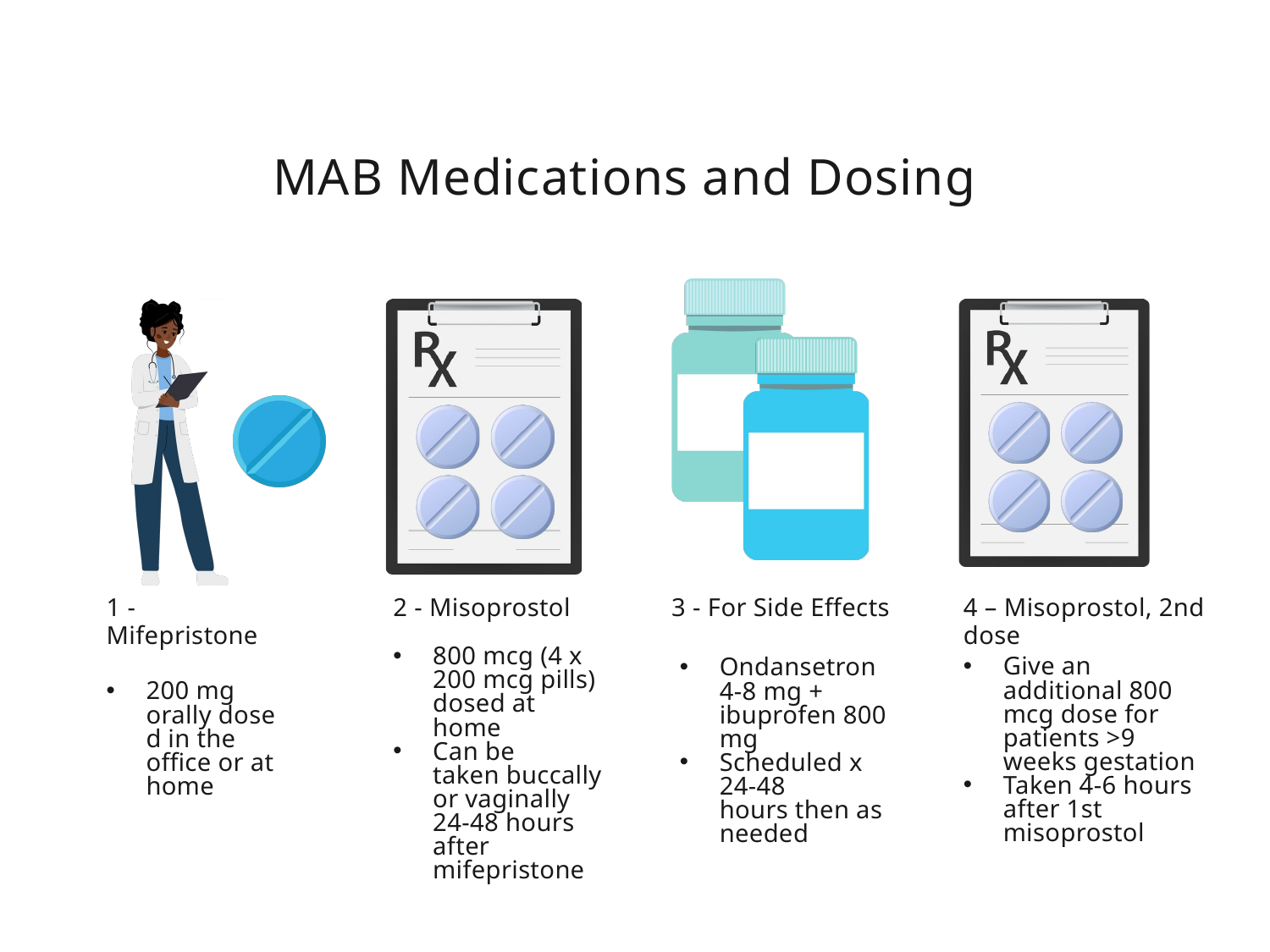

MAB Medications and Dosing
1 - Mifepristone
200 mg orally dosed in the office or at home
2 - Misoprostol
800 mcg (4 x 200 mcg pills) dosed at home
Can be taken buccally or vaginally 24-48 hours after mifepristone
4 – Misoprostol, 2nd dose
Give an additional 800 mcg dose for patients >9 weeks gestation
Taken 4-6 hours after 1st misoprostol
3 - For Side Effects
Ondansetron 4-8 mg + ibuprofen 800 mg
Scheduled x 24-48 hours then as needed

## Slide 25
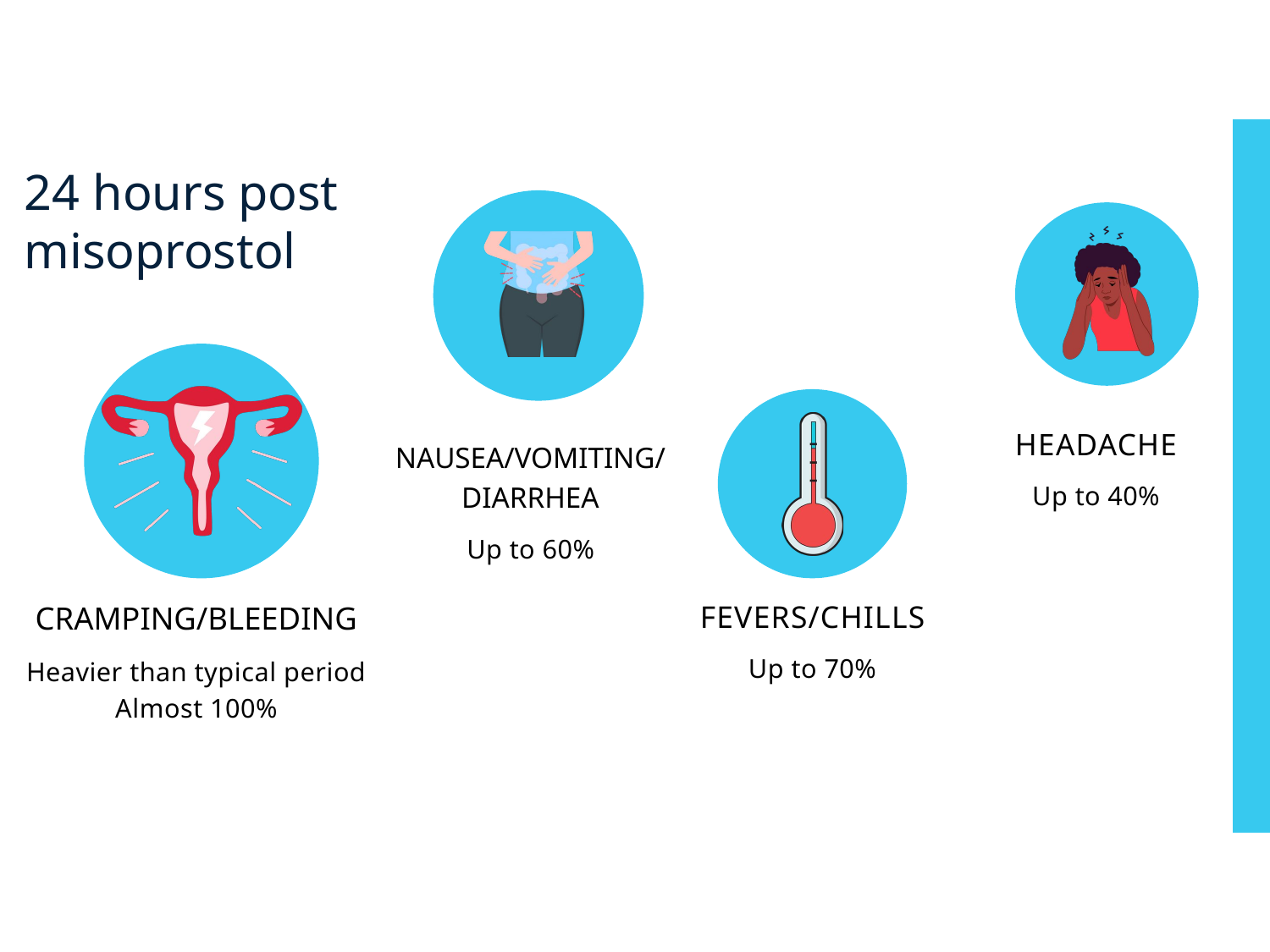

24 hours post misoprostol
HEADACHE
Up to 40%
NAUSEA/VOMITING/
DIARRHEA
Up to 60%
FEVERS/CHILLS
Up to 70%
CRAMPING/BLEEDING
Heavier than typical period Almost 100%

## Slide 26
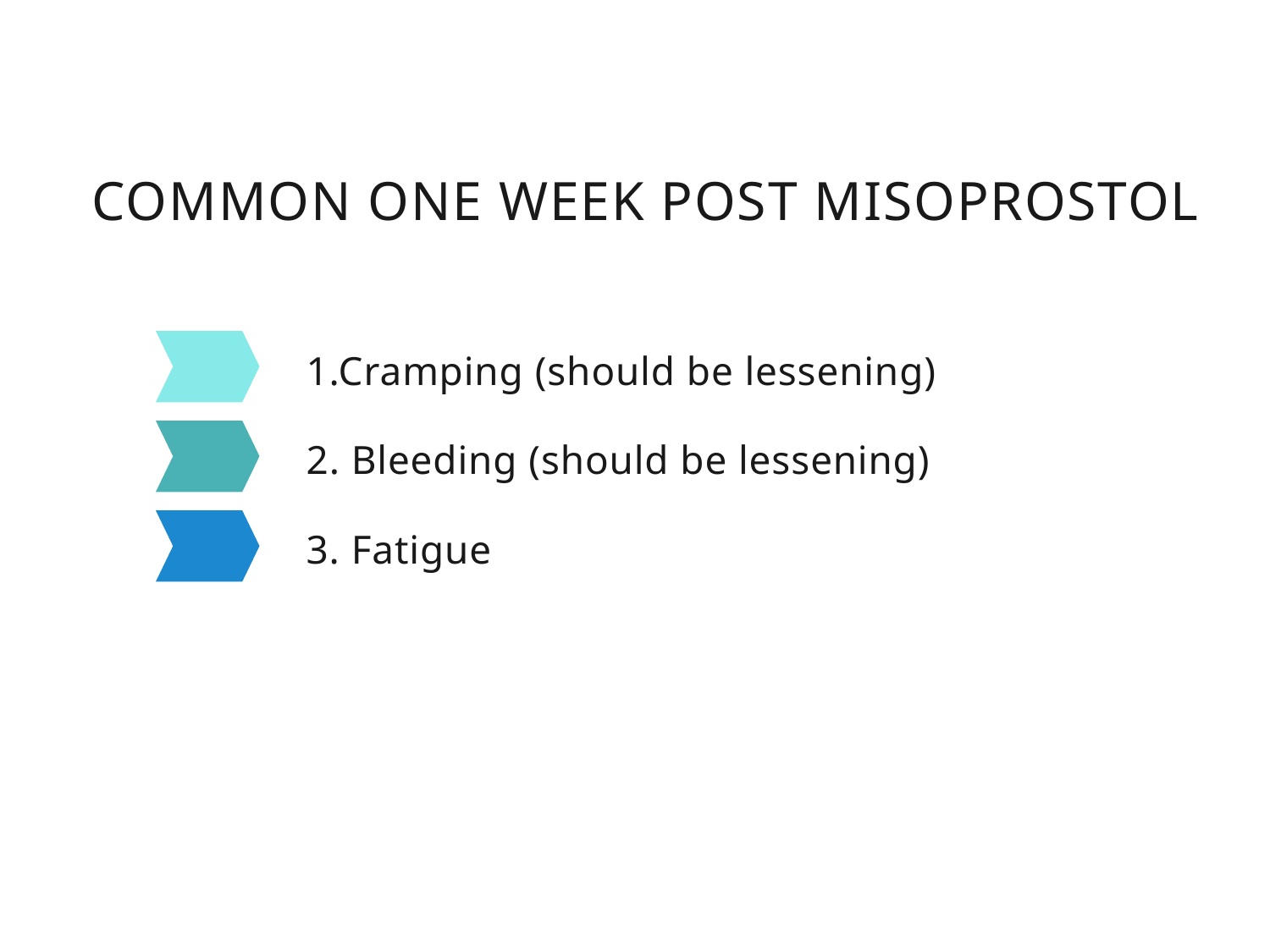

COMMON ONE WEEK POST MISOPROSTOL
1.Cramping (should be lessening)
2. Bleeding (should be lessening)
3. Fatigue

## Slide 27
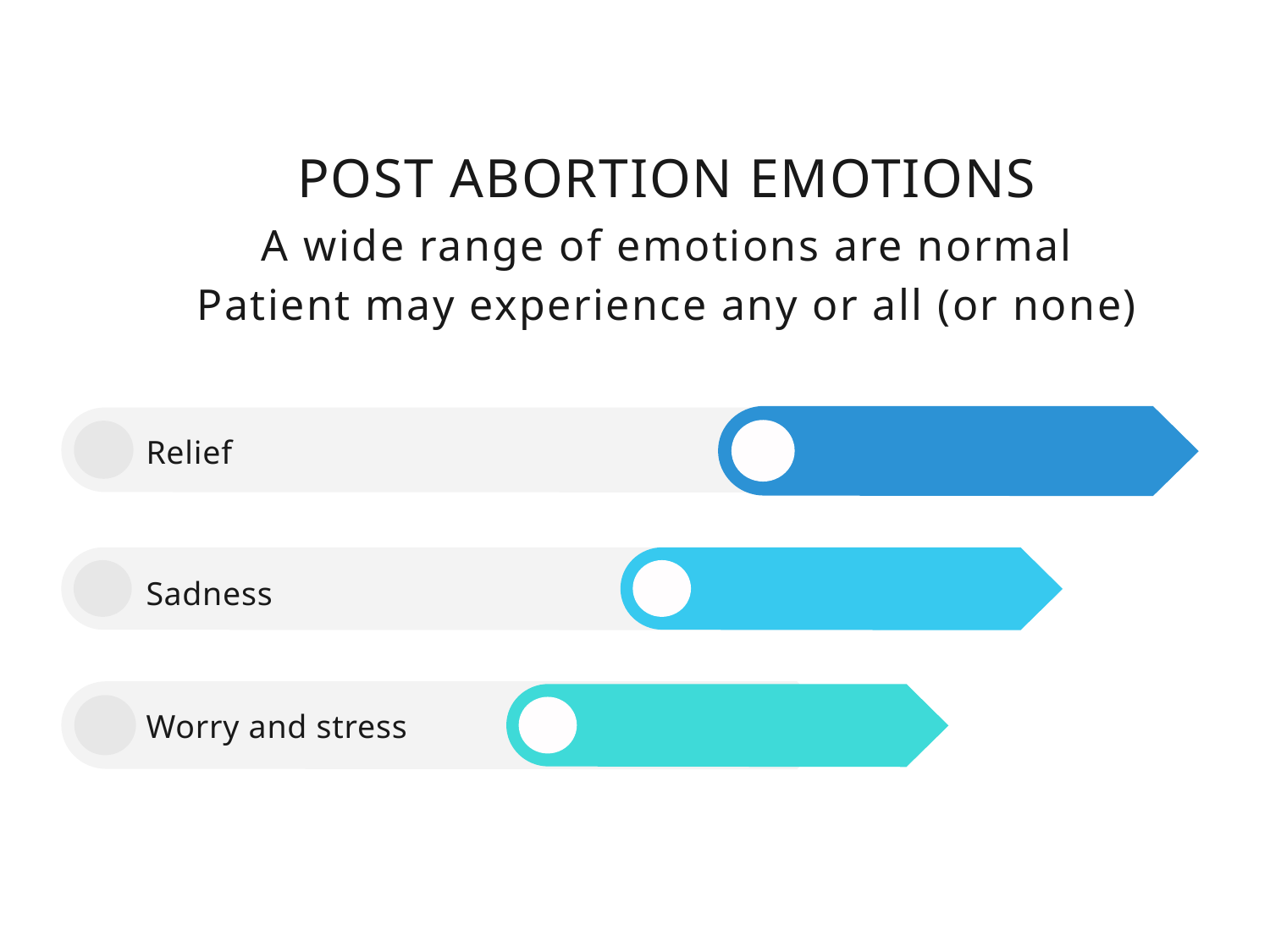

POST ABORTION EMOTIONS
A wide range of emotions are normal
Patient may experience any or all (or none)
Relief
Sadness
Worry and stress

## Slide 28
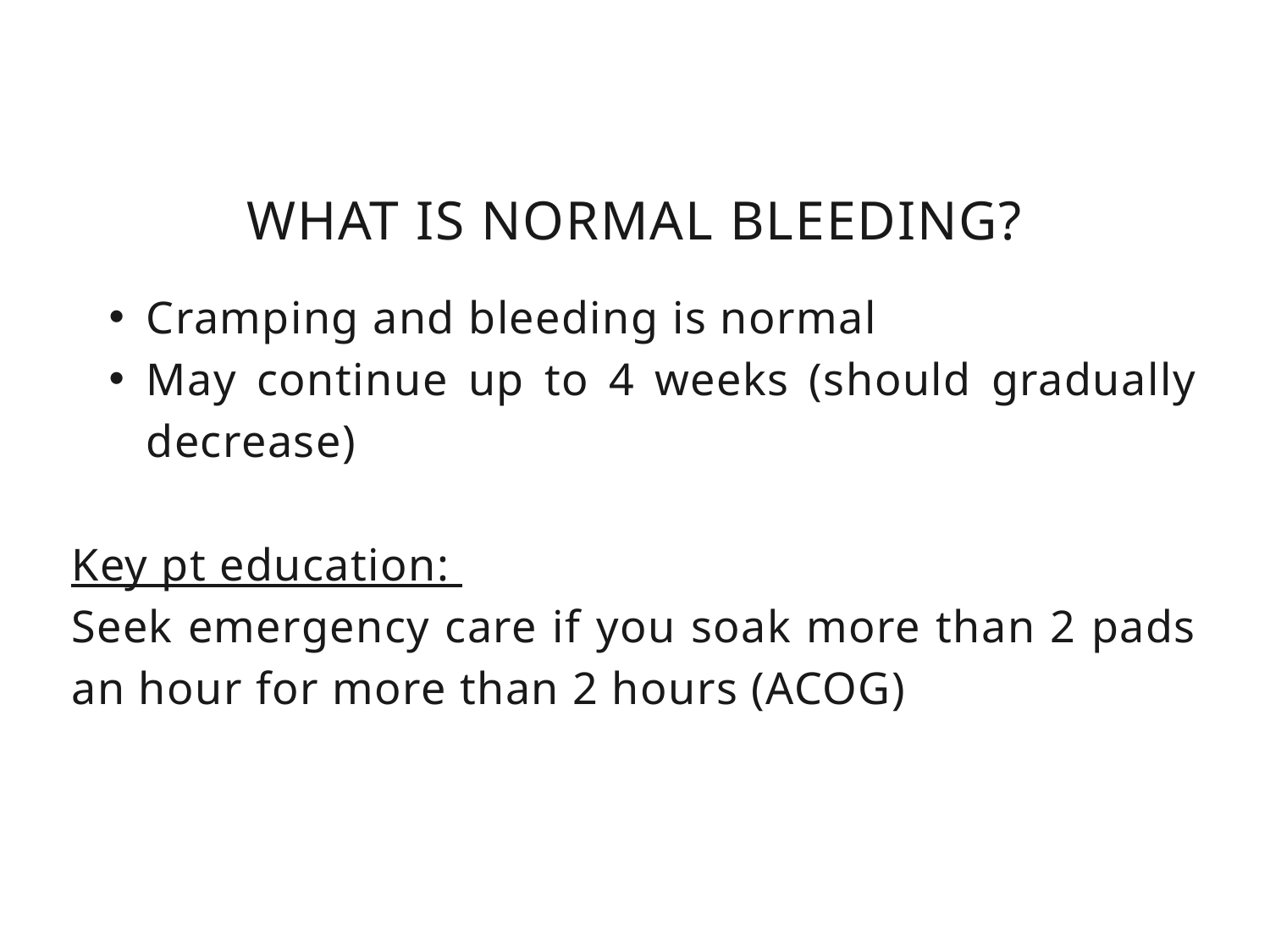

WHAT IS NORMAL BLEEDING?
Cramping and bleeding is normal
May continue up to 4 weeks (should gradually decrease)
Key pt education:
Seek emergency care if you soak more than 2 pads an hour for more than 2 hours (ACOG)

## Slide 29
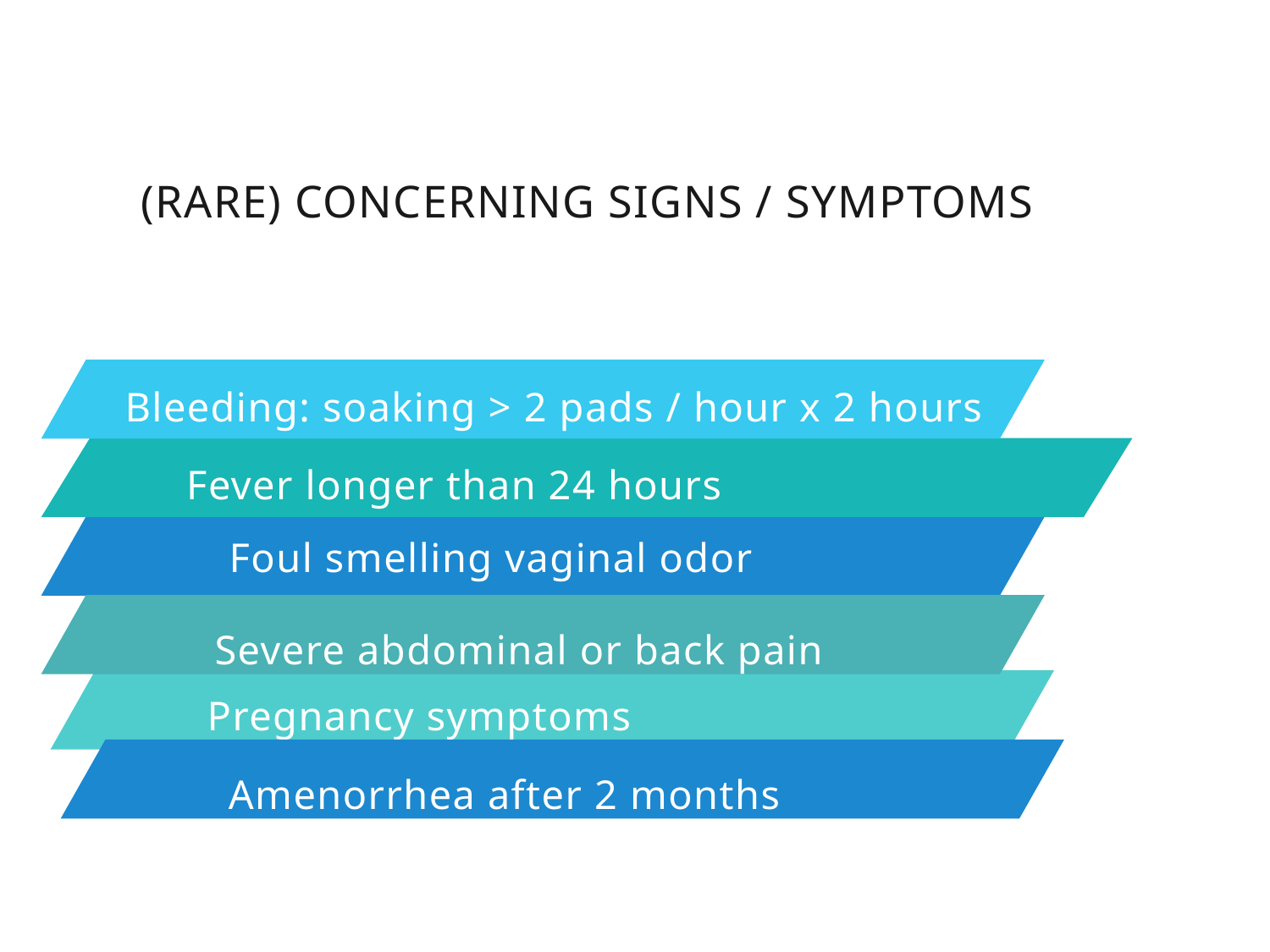

(RARE) CONCERNING SIGNS / SYMPTOMS
Bleeding: soaking > 2 pads / hour x 2 hours
Fever longer than 24 hours
Foul smelling vaginal odor
Severe abdominal or back pain
Pregnancy symptoms
Amenorrhea after 2 months
1
2
3
4
5
6

## Slide 30
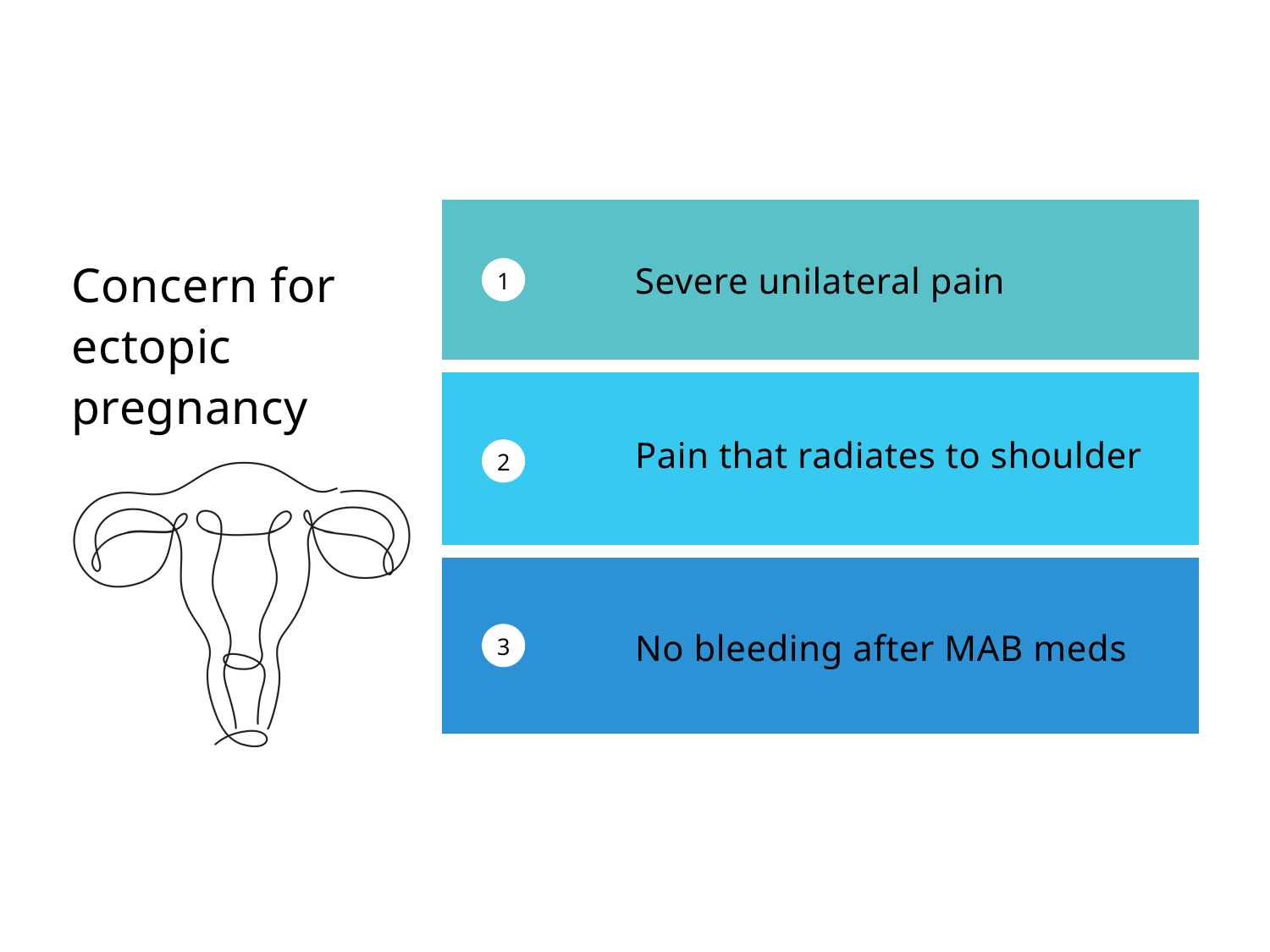

Concern for ectopic pregnancy
1
Severe unilateral pain
Pain that radiates to shoulder
2
3
No bleeding after MAB meds

## Slide 31
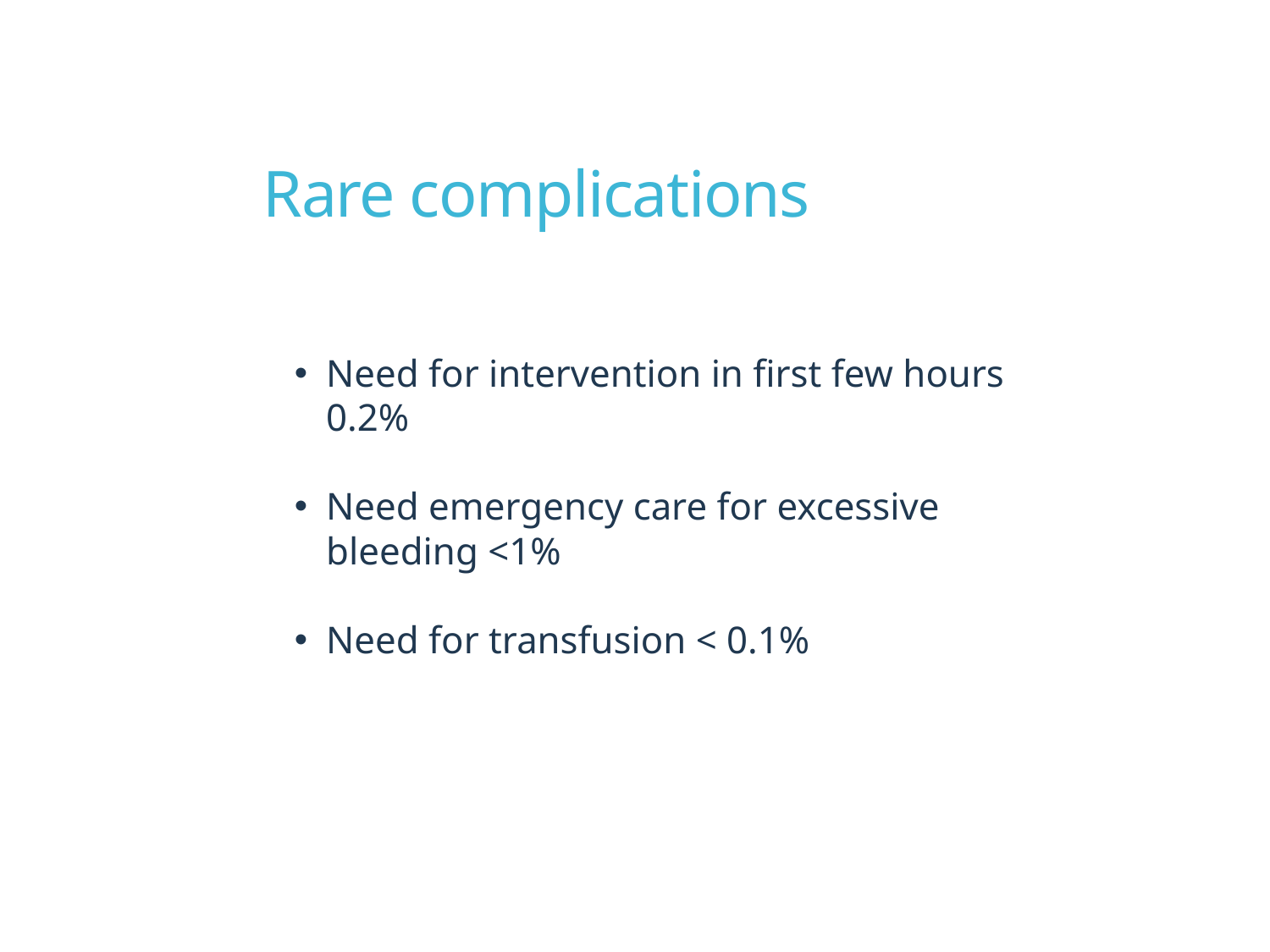

Rare complications
Need for intervention in first few hours 0.2%
Need emergency care for excessive bleeding <1%
Need for transfusion < 0.1%

## Slide 32
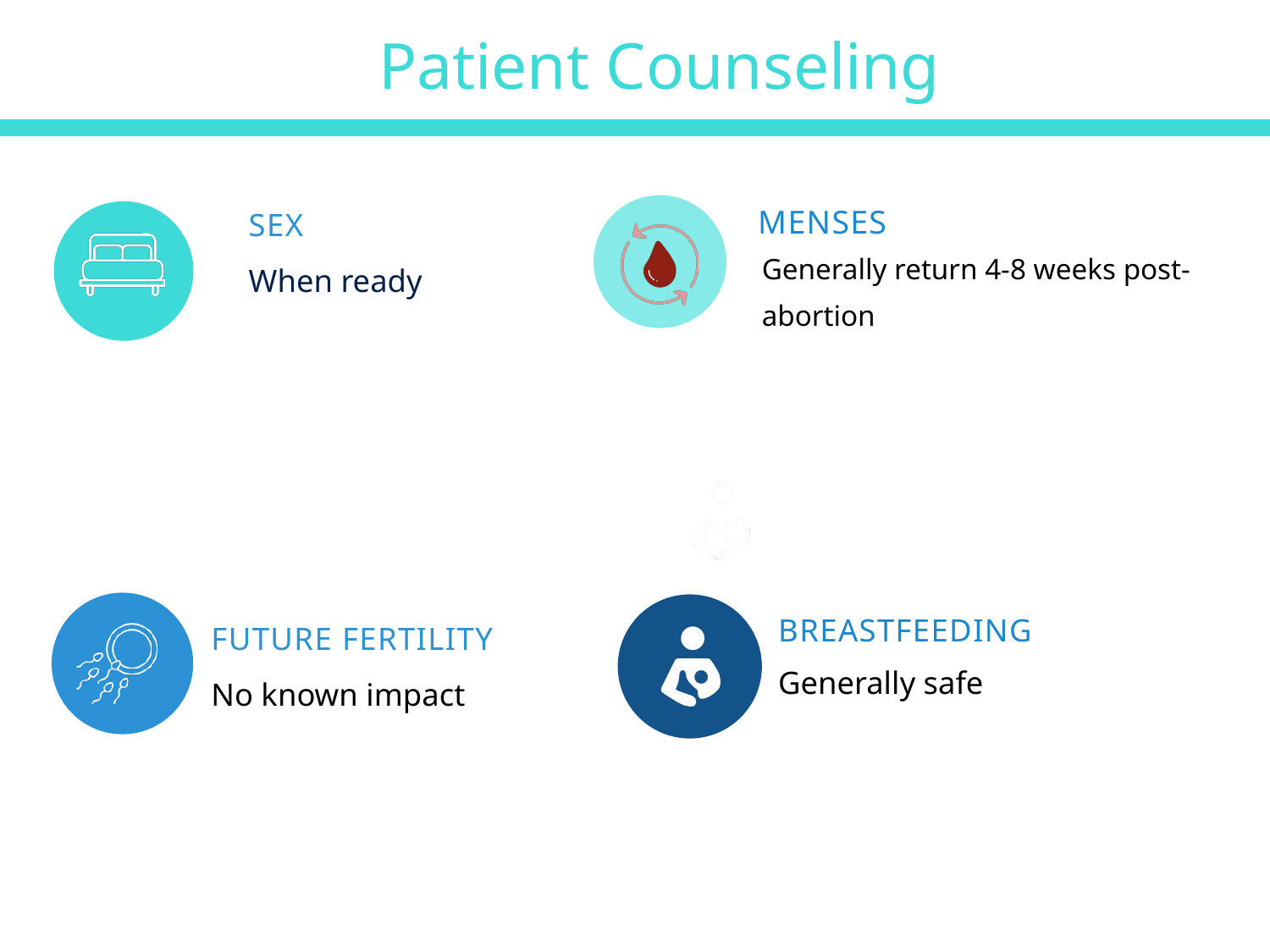

Patient Counseling
Generally return 4-8 weeks post-abortion
MENSES
SEX
When ready
BREASTFEEDING
Generally safe
FUTURE FERTILITY
No known impact

## Slide 33
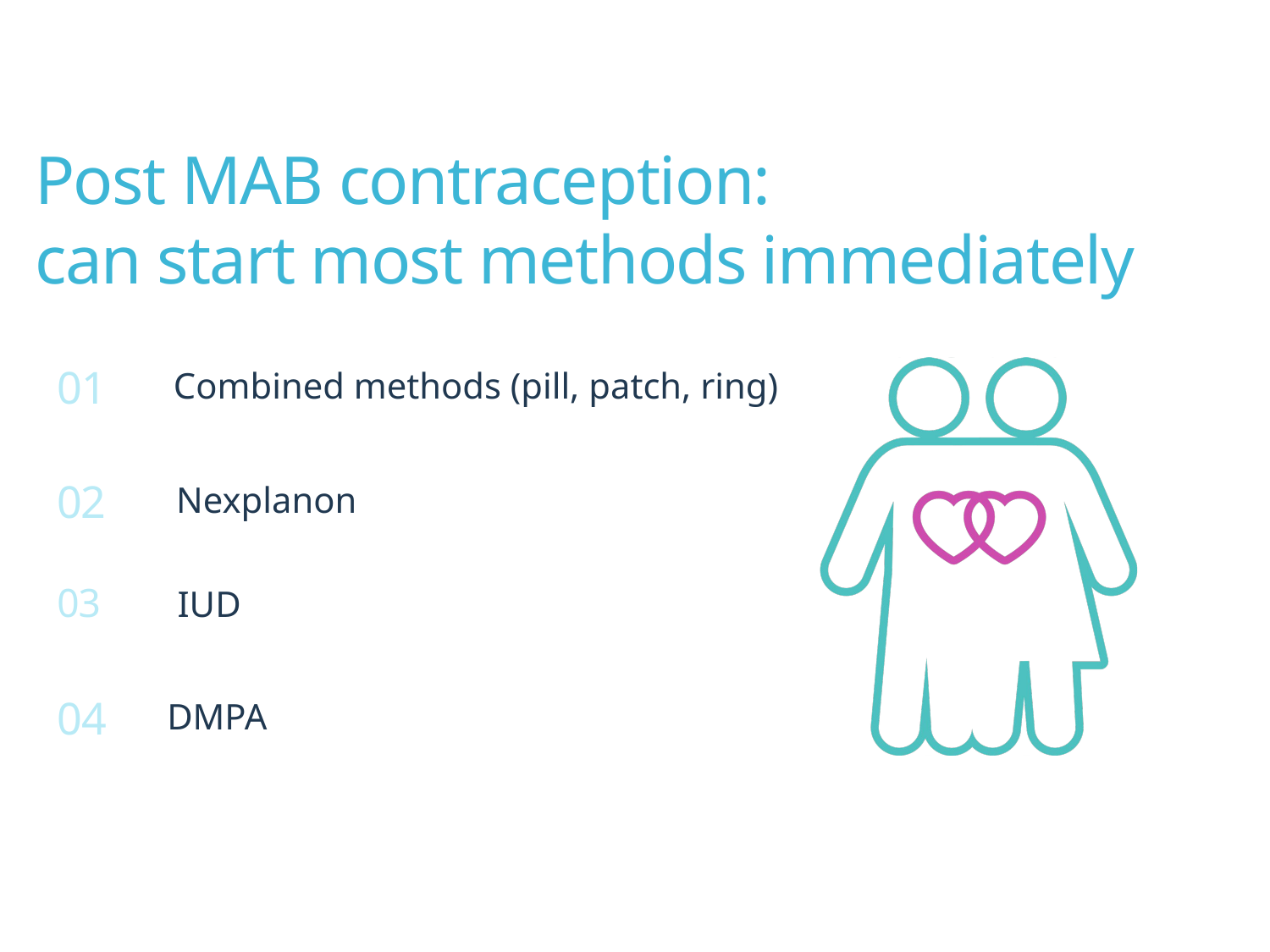

Post MAB contraception:
can start most methods immediately
Back to Agenda Page
01
Combined methods (pill, patch, ring)
02
Nexplanon
03
IUD
04
DMPA

## Slide 34
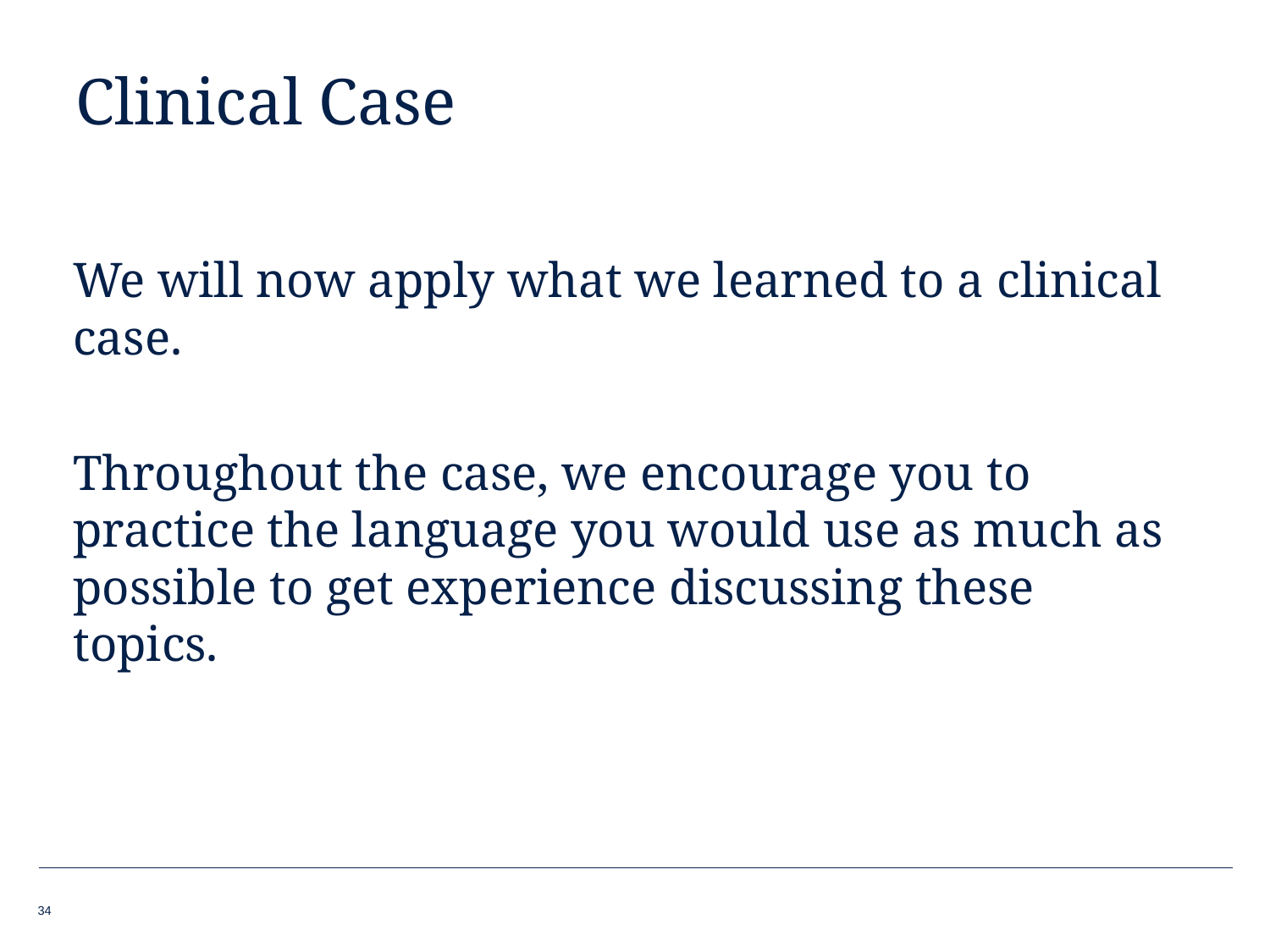

# Clinical Case
We will now apply what we learned to a clinical case.
Throughout the case, we encourage you to practice the language you would use as much as possible to get experience discussing these topics.
34

## Slide 35
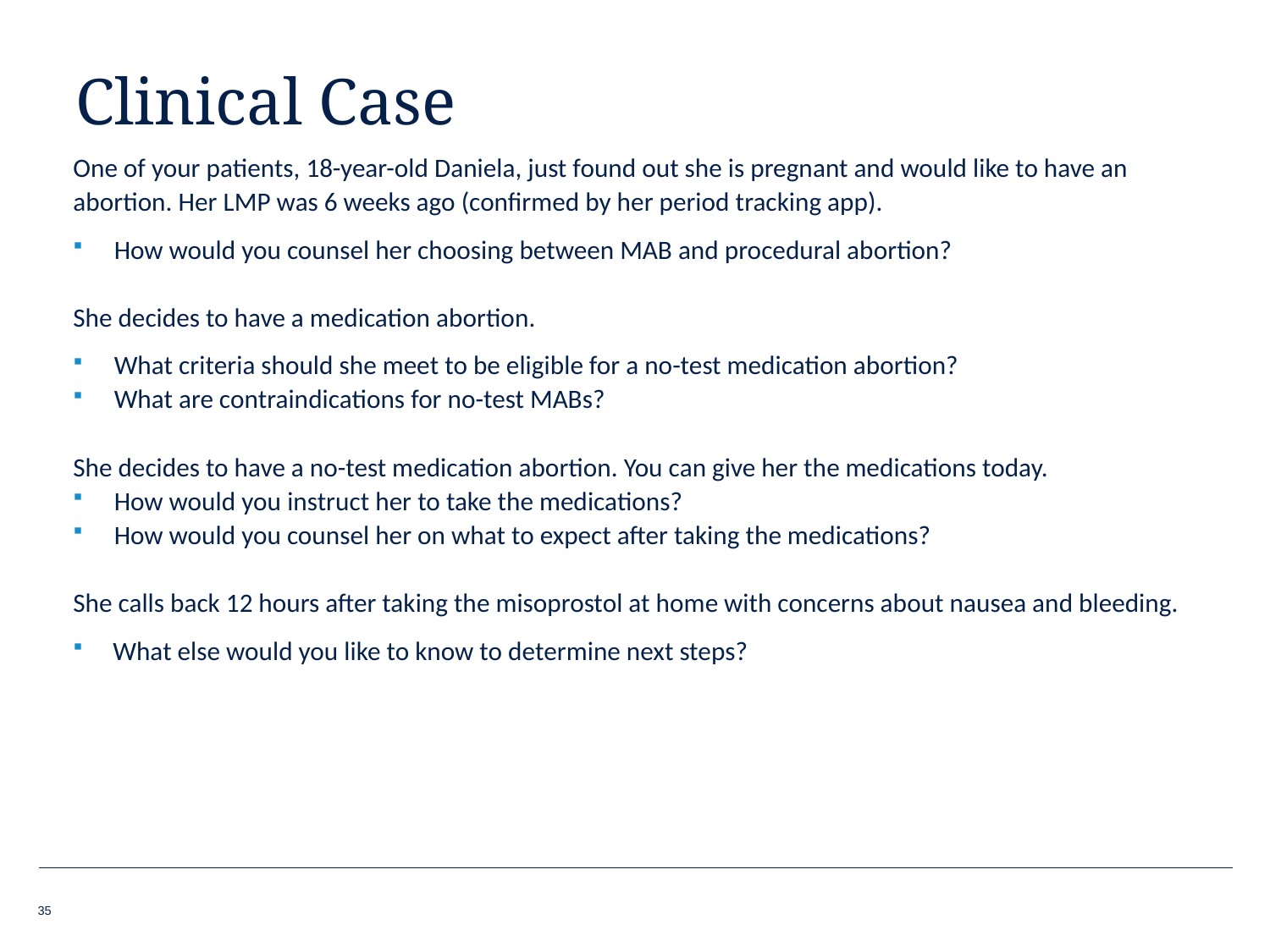

# Clinical Case
One of your patients, 18-year-old Daniela, just found out she is pregnant and would like to have an abortion. Her LMP was 6 weeks ago (confirmed by her period tracking app).
How would you counsel her choosing between MAB and procedural abortion?
She decides to have a medication abortion.
What criteria should she meet to be eligible for a no-test medication abortion?
What are contraindications for no-test MABs?
She decides to have a no-test medication abortion. You can give her the medications today.
How would you instruct her to take the medications?
How would you counsel her on what to expect after taking the medications?
She calls back 12 hours after taking the misoprostol at home with concerns about nausea and bleeding.
What else would you like to know to determine next steps?
35

## Slide 36
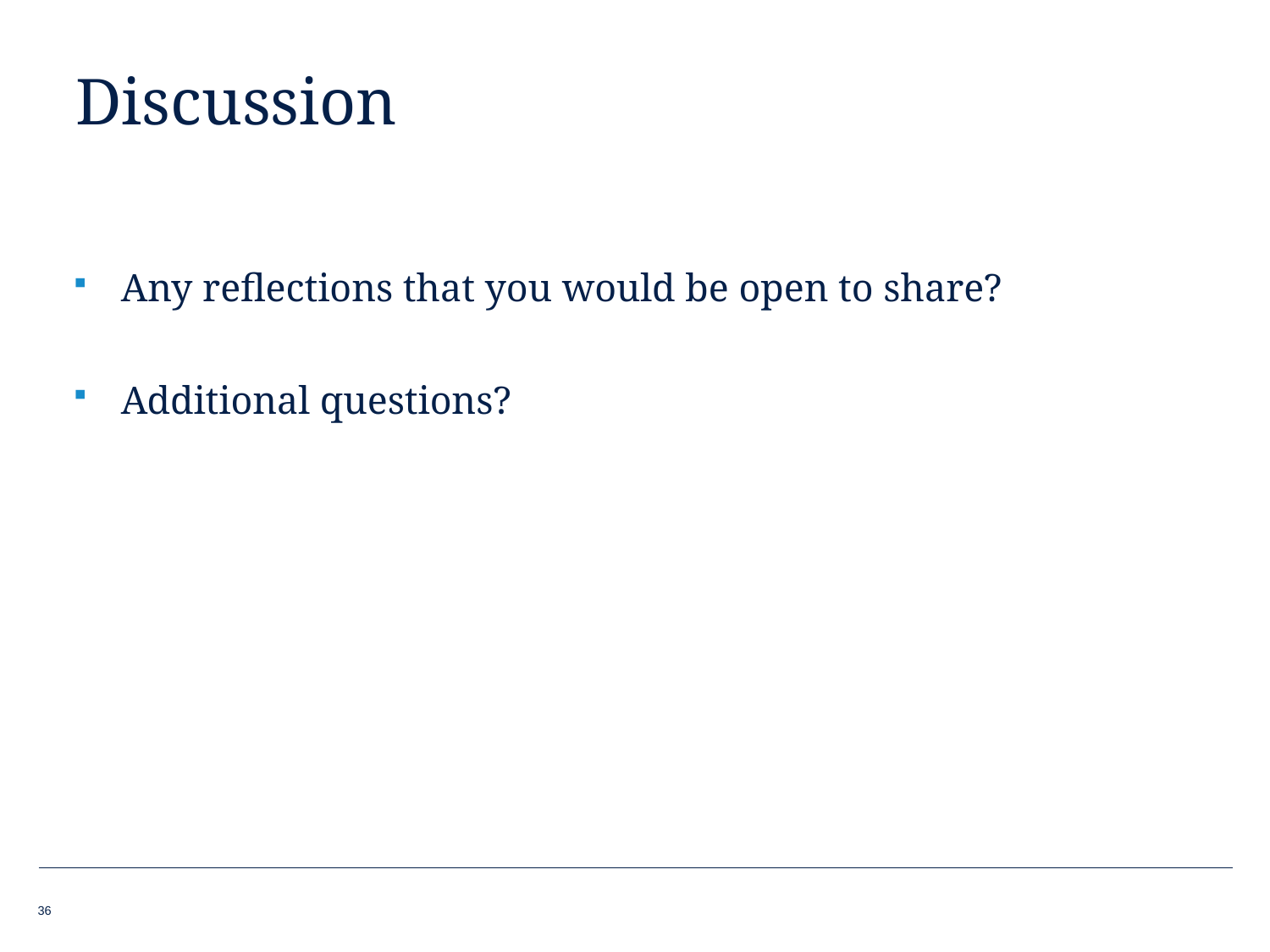

# Discussion
Any reflections that you would be open to share?
Additional questions?
36

## Slide 37
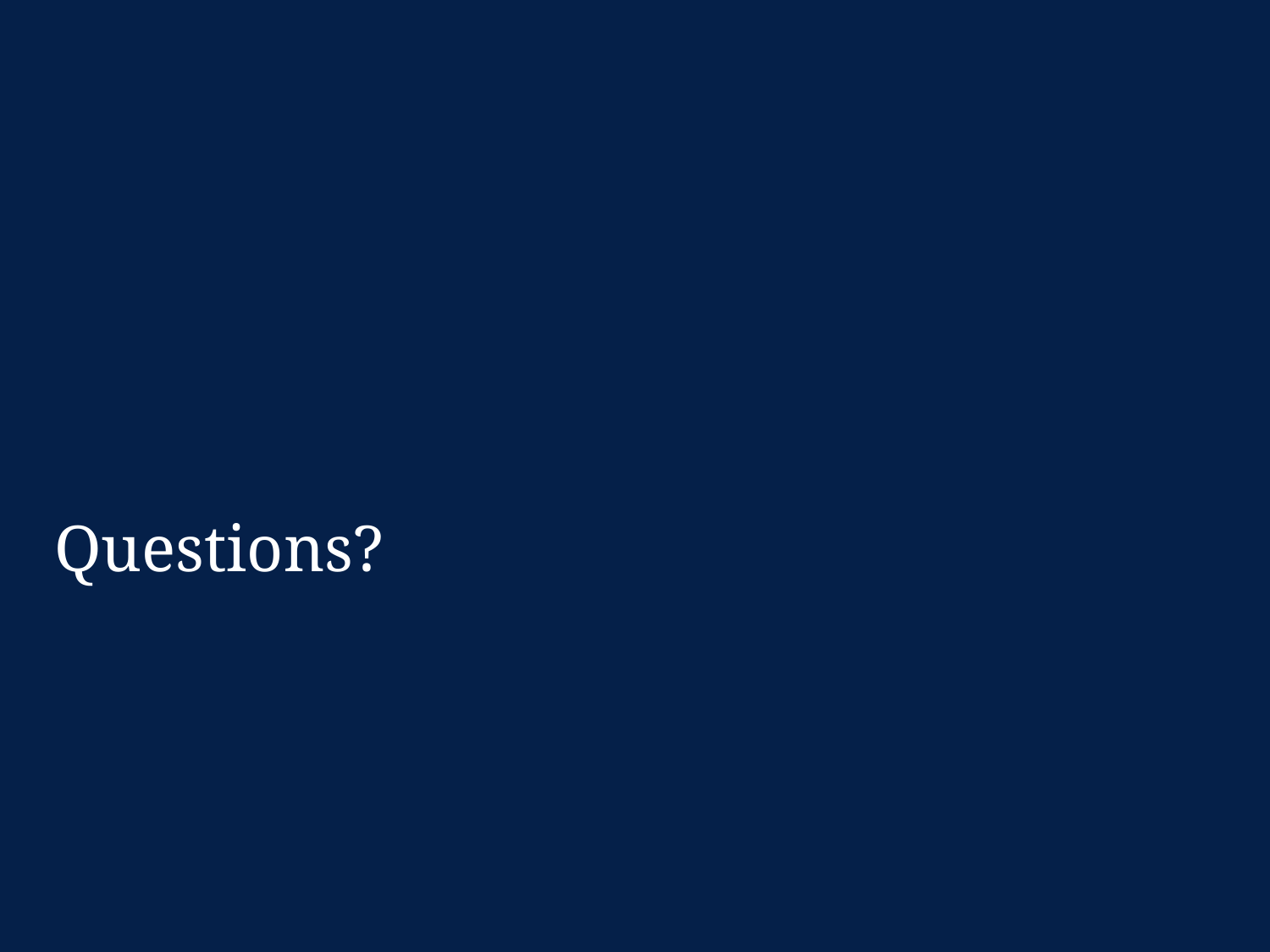

# Questions?

## Slide 38
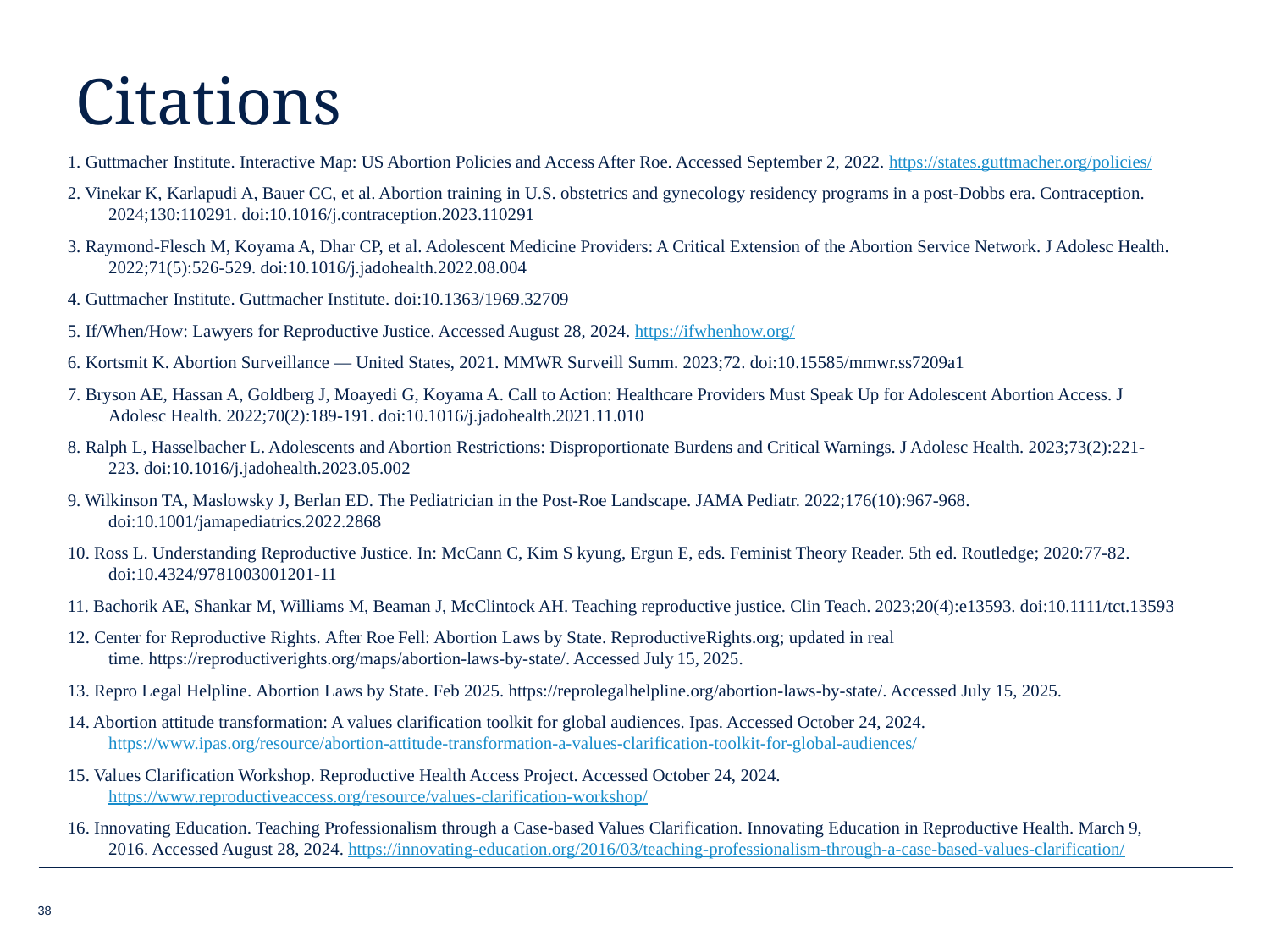

# Citations
1. Guttmacher Institute. Interactive Map: US Abortion Policies and Access After Roe. Accessed September 2, 2022. https://states.guttmacher.org/policies/
2. Vinekar K, Karlapudi A, Bauer CC, et al. Abortion training in U.S. obstetrics and gynecology residency programs in a post-Dobbs era. Contraception. 2024;130:110291. doi:10.1016/j.contraception.2023.110291
3. Raymond-Flesch M, Koyama A, Dhar CP, et al. Adolescent Medicine Providers: A Critical Extension of the Abortion Service Network. J Adolesc Health. 2022;71(5):526-529. doi:10.1016/j.jadohealth.2022.08.004
4. Guttmacher Institute. Guttmacher Institute. doi:10.1363/1969.32709
5. If/When/How: Lawyers for Reproductive Justice. Accessed August 28, 2024. https://ifwhenhow.org/
6. Kortsmit K. Abortion Surveillance — United States, 2021. MMWR Surveill Summ. 2023;72. doi:10.15585/mmwr.ss7209a1
7. Bryson AE, Hassan A, Goldberg J, Moayedi G, Koyama A. Call to Action: Healthcare Providers Must Speak Up for Adolescent Abortion Access. J Adolesc Health. 2022;70(2):189-191. doi:10.1016/j.jadohealth.2021.11.010
8. Ralph L, Hasselbacher L. Adolescents and Abortion Restrictions: Disproportionate Burdens and Critical Warnings. J Adolesc Health. 2023;73(2):221-223. doi:10.1016/j.jadohealth.2023.05.002
9. Wilkinson TA, Maslowsky J, Berlan ED. The Pediatrician in the Post-Roe Landscape. JAMA Pediatr. 2022;176(10):967-968. doi:10.1001/jamapediatrics.2022.2868
10. Ross L. Understanding Reproductive Justice. In: McCann C, Kim S kyung, Ergun E, eds. Feminist Theory Reader. 5th ed. Routledge; 2020:77-82. doi:10.4324/9781003001201-11
11. Bachorik AE, Shankar M, Williams M, Beaman J, McClintock AH. Teaching reproductive justice. Clin Teach. 2023;20(4):e13593. doi:10.1111/tct.13593
12. Center for Reproductive Rights. After Roe Fell: Abortion Laws by State. ReproductiveRights.org; updated in real time. https://reproductiverights.org/maps/abortion‑laws‑by‑state/. Accessed July 15, 2025.
13. Repro Legal Helpline. Abortion Laws by State. Feb 2025. https://reprolegalhelpline.org/abortion‑laws‐by‐state/. Accessed July 15, 2025.
14. Abortion attitude transformation: A values clarification toolkit for global audiences. Ipas. Accessed October 24, 2024. https://www.ipas.org/resource/abortion-attitude-transformation-a-values-clarification-toolkit-for-global-audiences/
15. Values Clarification Workshop. Reproductive Health Access Project. Accessed October 24, 2024. https://www.reproductiveaccess.org/resource/values-clarification-workshop/
16. Innovating Education. Teaching Professionalism through a Case-based Values Clarification. Innovating Education in Reproductive Health. March 9, 2016. Accessed August 28, 2024. https://innovating-education.org/2016/03/teaching-professionalism-through-a-case-based-values-clarification/
38

## Slide 39
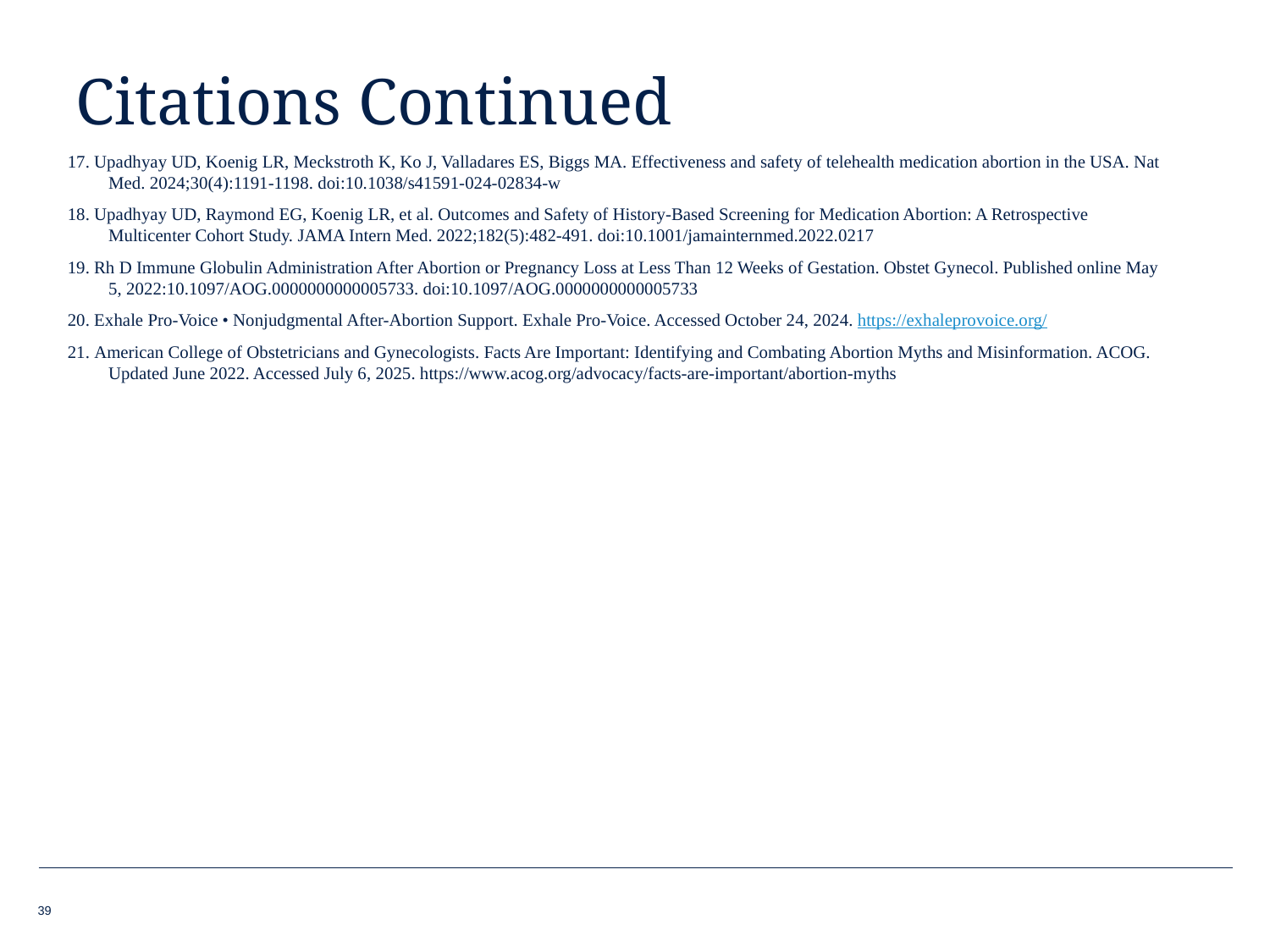

# Citations Continued
17. Upadhyay UD, Koenig LR, Meckstroth K, Ko J, Valladares ES, Biggs MA. Effectiveness and safety of telehealth medication abortion in the USA. Nat Med. 2024;30(4):1191-1198. doi:10.1038/s41591-024-02834-w
18. Upadhyay UD, Raymond EG, Koenig LR, et al. Outcomes and Safety of History-Based Screening for Medication Abortion: A Retrospective Multicenter Cohort Study. JAMA Intern Med. 2022;182(5):482-491. doi:10.1001/jamainternmed.2022.0217
19. Rh D Immune Globulin Administration After Abortion or Pregnancy Loss at Less Than 12 Weeks of Gestation. Obstet Gynecol. Published online May 5, 2022:10.1097/AOG.0000000000005733. doi:10.1097/AOG.0000000000005733
20. Exhale Pro-Voice • Nonjudgmental After-Abortion Support. Exhale Pro-Voice. Accessed October 24, 2024. https://exhaleprovoice.org/
21. American College of Obstetricians and Gynecologists. Facts Are Important: Identifying and Combating Abortion Myths and Misinformation. ACOG. Updated June 2022. Accessed July 6, 2025. https://www.acog.org/advocacy/facts-are-important/abortion-myths
39
