# Supplementary material for: A Pediatric- and Adolescent-Focused Medication Abortion Curriculum for Multidisciplinary Trainees
Source: MedEdPORTAL. 2025 Nov 13;21:11553. doi: 10.15766/mep_2374-8265.11553 (PMC12612281; doi:10.15766/mep_2374-8265.11553)
Supplement: Supplementary file 1 — Curriculum Facilitator Guide.docxModule 1 - Pregnancy Options.mp4Module 2 - Medication Abortion Management.mp4Module 3 - Postabortion Care.mp4Module 4 - Harm Reduction Strategies.mp4Workshop Slides.pptxCase.docxCase Facilitator Guide.docxPresurvey.docxPostsurvey.docxMAB Learner Resource Sheet.docx [file mep_2374-8265.11553-s001.zip › G. Case.docx]

Appendix G: Medication Abortion Case - Learner Version

*This document should be provided to learners prior to or during workshop to work through an adolescent MAB case.*

**Learning Objectives:**

1. Practice counseling on medication and procedural abortions
2. Describe key concepts in anticipatory guidance for MABs
3. Explain complications of MABs that need further evaluation and intervention.

**Clinical Case:**

One of your patients, 18 yo Daniela, just found out she is pregnant and would like to have an abortion. Her LMP was 6 weeks ago (confirmed by her period tracking app). ​

- How would you counsel her choosing between MAB and procedural abortion? ​
  ​

She decides to have a medication abortion.​

- What criteria should she meet to be eligible for a no-test medication abortion? ​
- What are contraindications for no-test MABs?​

​
She decides to have a no-test medication abortion. You can give her the medications today. ​

- How would you instruct her to take the medications?​
- How would you counsel her on what to expect after taking the medications? ​

​
She calls back 12 hours after taking the misoprostol at home with concerns about significant nausea and heavy bleeding. ​

- What else would you like to know to determine next steps?​
- When should she seek emergency care?
